# One-pot reaction of 3-vinylchromones, aromatic aldehydes, and ammonium acetate: an efficient approach to highly functionalized 1,6-dihydropyridine derivatives†

**DOI:** 10.1039/d4ra07863b

**Published:** 2025-01-27

**Authors:** Behnaz Farajpour, Marzie Kakaie, Faiq H. S. Hussain, Fataneh Rakaee, Fatemeh Moradkhani, Morteza Shiri

**Affiliations:** a Department of Organic Chemistry, Faculty of Chemistry, Alzahra University Vanak Tehran 1993893973 Iran mshiri@alzahra.ac.ir; b Medical Analysis Department, Applied Science Faculty, Tishk International University Erbil 44001 Kurdistan Region Iraq; c Department of Medicinal Chemistry, Faculty of Pharmacy and Pharmaceutical Sciences Research Center, Tehran University of Medical Sciences Tehran P94V+8MF Iran

## Abstract

In this work, we present an efficient strategy for the straightforward synthesis of functionalized 1,6-dihydropyridine derivatives *via* a three-component reaction of 3-vinylchromones, aromatic aldehydes, and ammonium acetate. A tandem procedure including *in situ* NH aldimine formation/Michael-type addition/opening of the pyrone ring/isomerization/6π-electrocyclization/[1,5]-H shift allows rapid access to a series of dihydropyridines bearing an *ortho*-hydroxybenzoyl and a benzoyl scaffold in good yields. Readily available precursors, simple heating conditions, and operational simplicity are some highlighted advantages of this transformation.

## Abstract 2

Herein, we present an efficient method for the synthesis of functionalized 1,6-dihydropyridine derivatives. Readily available precursors, operational simplicity, and synthetically useful yields are some highlighted advantages of this transformation.

## Graphical abstract

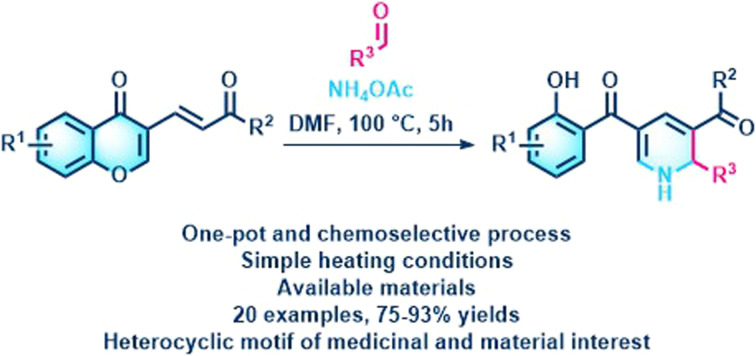

## Introduction

Dihydropyridines (DHPs) represent an interesting family of heterocycles based on the pyridine ring.^1^ Five isomeric structures are possible for this class of organic compounds.^2^ Regarding the scaffold of dihydropyridines, 1,4- and 1,2- or 1,6-DHPs constitute the most populated group.^3^ Although 1,4-DHPs have garnered much interest due to their extensive biological and pharmacological activities,^4^ 1,2-DHPs are mostly known as versatile precursors to complex organic functional materials and are frequently employed as building blocks.^5^

**Fig. 1:**
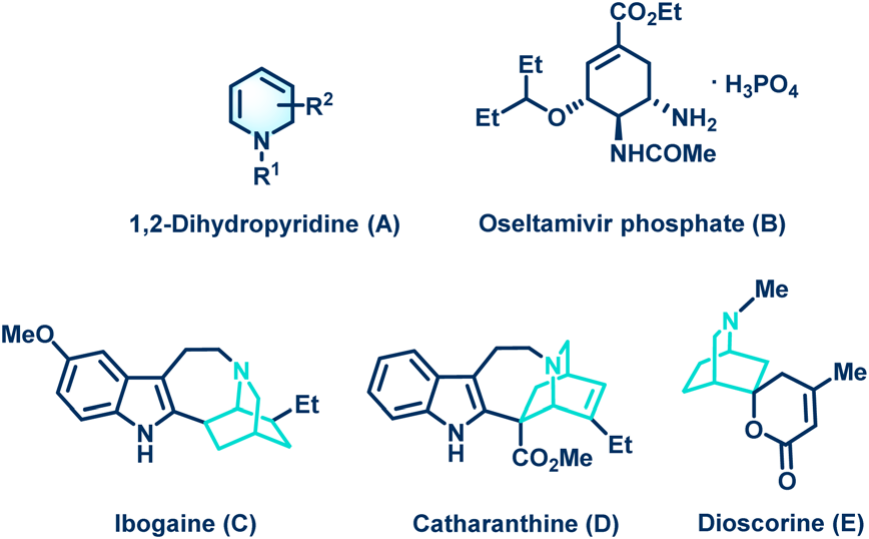
Structures of 1,2-dihydropyridines (A), oseltamivir phosphate (B), and some pharmaceutically active molecules containing the isoquinuclidine unit (C–E).

**Scheme 1:**
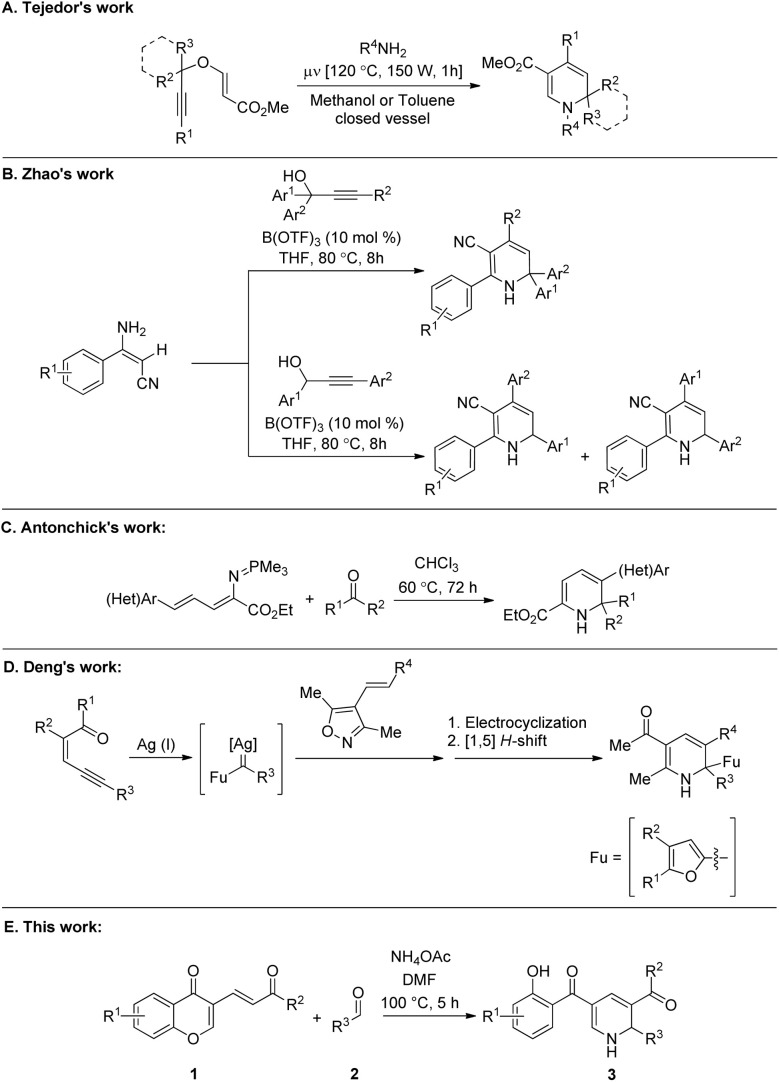
Synthesis of functionalized dihydropyridine derivatives.

For example, 1,2-DHPs can be used as cyclic aza-dienes in the Diels-Alder reaction for the synthesis of isoquinuclidines (2-azabicyclo[2.2.2]octanes).^6^ Notably, isoquinuclidines are valuable synthons for the synthesis of some privileged medicinal scaffolds such as oseltamivir phosphate.^7^ More specifically, the isoquinuclidine ring system is present in pharmacologically relevant molecules such as the alkaloids ibogaine, catharanthine, and dioscorine (Fig. 1).^8^ Consequently, the development of novel and efficient methods for synthesizing substituted 1,2-dihydropyridines is an interesting topic for organic chemists. Notably, various synthetic routes for the preparation of substituted 1,2-DHPs have been reviewed in 2013 by Silva and co-workers.^9^ Moreover, several graceful methodologies for the synthesis of 1,2-dihydropyridine derivatives have been developed in the past years.^10^ For example, Tejedor and co-workers reported the synthesis of substituted 1,2-DHPs through a microwave-assisted domino reaction of propargyl vinyl ethers and primary amines (Scheme 1A).^11^ The Lewis acid-catalyzed annulation of propargylic alcohols with (*E*)-3-amino-3-phenylacrylonitriles for the synthesis of functionalized 1,2-dihydropyridine derivatives was illustrated by Zhao's group (Scheme 1B).^12^ Antonchick *et al.* demonstrated the metal-free synthesis of substituted 1,2-dihydropyridines with quaternary stereogenic centers using a cascade aza-Wittig/6π-electrocyclization process (Scheme 1C).^13^ Very recently, Deng's group disclosed the elegant synthesis of functionalized 1,2-dihydropyridines *via* a silver(i)-catalyzed tandem reaction of enynones and 4-alkenyl isoxazoles (Scheme 1D).^14^ Although these reported methods make a considerable contribution for the construction of structurally diverse 1,2-dihydropyridine scaffolds, some of them are limited by the employment of sensitive or expensive prefunctionalized starting materials, transition-metal catalysts, and tedious multistep transformations. In this regard, the development of efficient, innovative, and simple strategies to overcome these limitations is highly desirable. Notably, chromone derivatives^15^ occupy an important position in the preparation of various organic scaffolds due to their ability to undergo ring-opening reactions.^16^ Having interest in developing novel routes for the construction of privileged organic architectures,^17^ herein we considered the use of aldehydes and ammonium acetate as reaction partners to 3-vinyl chromones^16*e*^ for the preparation of functionalized dihydropyridine derivatives (Scheme 1E).

Interestingly, this multicomponent strategy incorporates benzoyl and *ortho*-hydroxybenzoyl frameworks into a 1,2-dihydropyridine core. Furthermore, inexpensive and available materials, simple heating conditions, and satisfactory yields are some highlighted advantages of this practical route.

## Results and discussion

We started our studies by evaluating the reaction of 3-vinyl chromone 1a (1 equiv.), benzaldehyde 2a (1 equiv.), and ammonium acetate (2 equiv.) in acetonitrile under an ambient atmosphere. When the reaction was run at room temperature, the starting materials remained unreacted and small quantities of by-products were formed (Table 1, entry 1). Gratifyingly, clean conversion was observed upon increasing the reaction temperature to 80 °C and product 3a was generated in 59% yield (entry 2).

**Table 1:** Survey on the conditions for the synthesis of 3a^a^

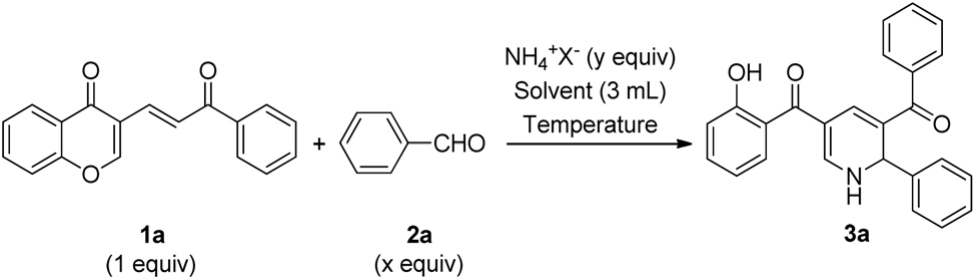						
Entry	*x*	[NH_4_]^+^ (*y*)	Solvent (3 mL)	Temperature (°C)	Time (h)	Yield (%)^b^
1	1	NH_4_OAc (2)	MeCN	25	24	0
2	1	NH_4_OAc (2)	MeCN	80	16	59
3	1	NH_4_OAc (2)	EtOH	80	15	57
4	1	NH_4_OAc (2)	DMSO	100	10	72
5	1	NH_4_OAc (2)	DMF	100	10	77
6	1	NH_4_OAc (2)	CHCl_3_	60	24	Trace
7	1	NH_4_OAc (2)	*n*-hexane	70	24	Trace
8	1	NH_4_OAc (2)	THF	65	24	Trace
9	1	NH_4_Cl (2)	DMF	100	24	14
10	1	(NH_4_)_2_SO_4_ (2)	DMF	100	24	11
11	1	NH_4_OAc (3)	DMF	100	9	81
12	1	NH_4_OAc (4)	DMF	100	7	84
13	1	NH_4_OAc (5)	DMF	100	7	79
14	1.5	NH_4_OAc (4)	DMF	100	5	89
15	2	NH_4_OAc (4)	DMF	100	5	80

a Reaction conditions: a mixture of 1a (1 equiv.), 2a (*x* equiv.), and ammonia source (*y* equiv.) in the mentioned solvent (3 mL) was magnetically stirred at the mentioned temperature in an oil bath. The target product was purified by column chromatography on silica gel using *n*-hexane/EtOAc (5 : 1 v/v) as the eluent.

b Isolated yields.

Encouraged by this initial result, different solvents were screened to optimize the reaction conditions. Compared with acetonitrile, ethanol afforded a comparative yield (entry 3).

A significant improvement in the reaction yield to 72% and the reaction time to 10 h was achieved by using dimethyl sulfoxide (DMSO) as the solvent by heating the reaction to 100 °C (entry 4). Furthermore, the reaction yield was marginally increased, from 72% to 77%, by changing the solvent from DMSO to dimethylformamide (DMF) (entry 5). Notably, some solvents, including chloroform, *n*-hexane, and tetrahydrofuran (THF), proved to be unusable for this process (entries 6–8). With the aim of further improving the reaction yield, various ammonia sources were evaluated. Results revealed that ammonium acetate is the best ammonia source for this reaction (entries 9 and 10). Further research showed that the amounts of starting materials are crucial for this transformation (entries 11–15), and the best result, 89% yield, was gained when the ratio of 3-vinyl chromone 1a, benzaldehyde 2a, and ammonium acetate was adjusted to 1 : 1.5 : 4. Therefore, the conditions of entry 14 were determined as the optimal conditions.

**Scheme 2:**
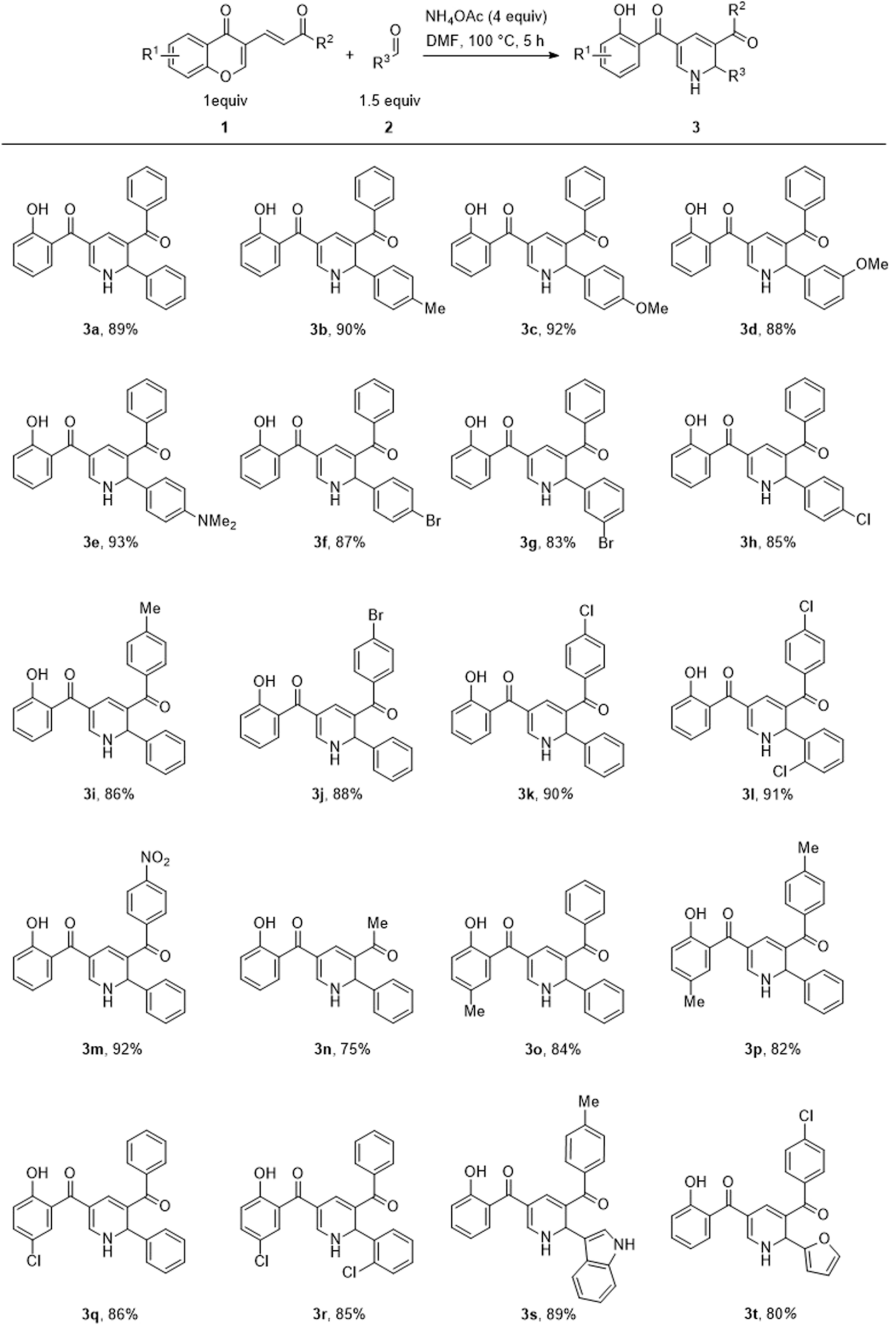
Synthesis of various substituted dihydropyridine derivatives.

**Fig. 2:**
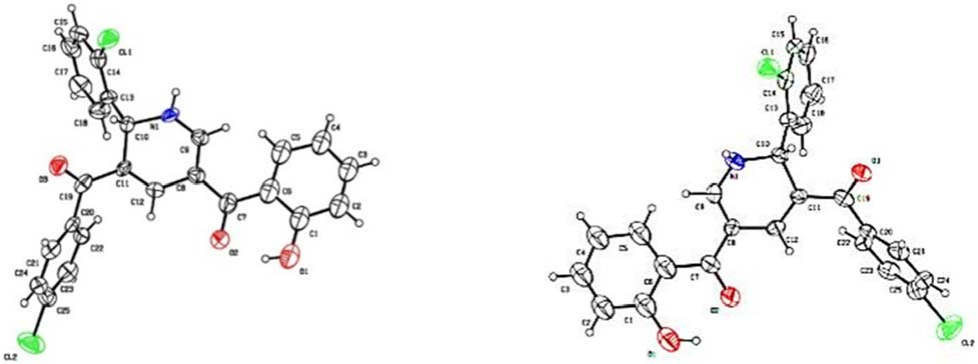
ORTEP plot for the crystal structure of 3l-*R* enantiomer (left) and 3l-*S* enantiomer (right) (CCDC 2364260 and 2364261). Thermal ellipsoids are at 30% probability level.

**Scheme 3:**
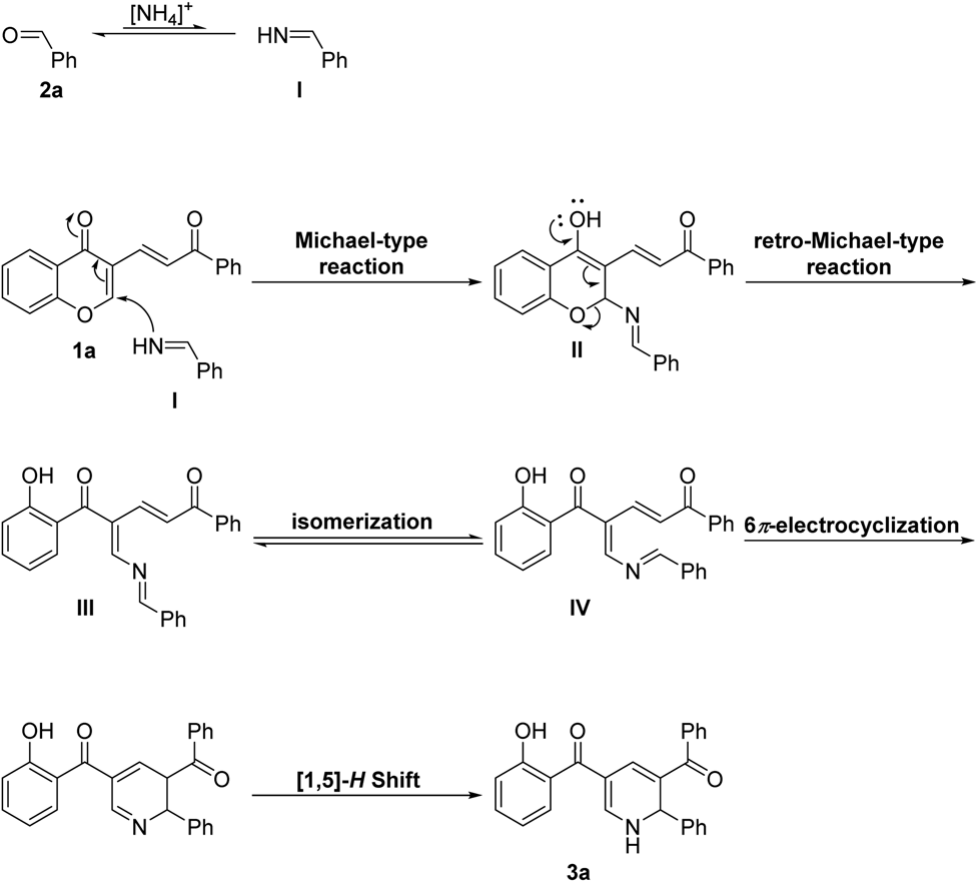
Proposed reaction mechanism for the formation of 3a.

To gain insight into the versatility of this transformation, we set out to explore the reaction scope using various aldehydes and 3-vinyl chromone derivatives under the optimized conditions (Scheme 2). We first investigated the reaction efficiency with respect to diverse benzaldehyde derivatives. The results revealed that various substituted benzaldehydes, possessing electron-donating or weak electron-withdrawing substituents, were tolerated in this process, affording the desired products in good yields (3b–3h, 83–93%). However, for benzaldehydes with strong electron-withdrawing groups (CN, NO_2_), the desired products were not formed. To further verify the generality of the reaction, aliphatic aldehydes such as butyraldehyde and decyl aldehyde were tested, but the target products were formed in trace amounts. These results could be related to the intrinsically lower stability of aliphatic aldimines due to their susceptibility to decomposition, self-condensation, and imine–enamine tautomerization.^18^ Subsequently, the scope of 3-vinylchromones was explored. To our delight, differently substituted 3-vinylchromones, possessing various *R*^1^ and *R*^2^ substituents, were compatible with this process and were successfully converted to the desired products (3i–3r, 75–92%). The developed strategy was also effective for some heterocyclic aldehydes including 3-formyl indole and 2-formyl furan delivering the products 3s and 3t in 89% and 80% yield, respectively. In the case of other heterocyclic aldehydes, including 2-formyl indole, 2-formyl pyrrole, 4-chloro-3-formyl coumarin, and 2-chloro-3-formyl quinoline, several overlapping spots were formed and the isolation process was very difficult. To investigate the synthetic utility of this method, the gram-scale synthesis of 3a was explored. In this regard, the reaction of 3-vinylchromone 1a (4 mmol, 1104 mg), benzaldehyde 2a (6 mmol, 636 mg), and ammonium acetate (16 mmol, 1233 mg) proceeded well, affording the desired product 3a in 80% yield (1219 mg) without a significant loss of efficiency compared to the small-scale experiment (89% yield). The structures of the products were characterized by high-resolution mass spectrometry (HRMS) analysis, and nuclear magnetic resonance (NMR). Moreover, the structure of 3l was undeniably elucidated by using X-ray crystallographic analysis. Interestingly, in the crystallization process, we faced with the spontaneous resolution of enantiomers. Therefore, we reported the crystal structure of both in this paper (Fig. 2). Based on the above results and related literature,^19^ a putative reaction mechanism is depicted in Scheme 3 (with 3a as the example). The reaction begins with the formation of NH aldimine intermediate I through the condensation of 2a with ammonia. The *in situ* formed intermediate I then reacts with 3-vinylchromone 1a to provide intermediate II*via* a Michael-type reaction.

The process is followed by the opening of the pyrone ring in II to deliver 2-aza-triene intermediate III. In continue, the s*-trans*, s*-cis* intermediate III should be converted to the cyclization-reactive *s-cis*, *s-cis* intermediate IV in order to undergo 6π-electrocyclization reaction. Notably, the conformer IV is thermodynamically unfavored and can be formed under thermal conditions. The subsequent thermal 6π-electrocyclization of intermediate IV followed by a [1,5]-H Shift^13^ process affords final product 3a.

In summary, an unprecedented catalyst-free reaction of 3-vinyl-4-chromones, aromatic aldehydes, and ammonium acetate has been developed.

This one-pot transformation, carried out under simple heating conditions, provides access to substituted dihydropyridine derivatives in synthetically useful yields. The overall process (formation of one C–C bond and two C–N bonds) may include the following steps: NH aldimines formation/Michael-type addition/opening of the pyrone ring/isomerization/6π-electrocyclization/[1,5]-H shift.

Furthermore, the presence of various substituents around the dihydropyridine core could furnish attractive points of postfunctionalization to afford a series of useful compounds that are difficult to prepare with other methods.

Further studies probing the usefulness of this strategy and synthetic applications of the products are ongoing in our laboratory.

## Data availability

The data supporting the findings of this study are available within the article and ESI.†

## Conflicts of interest

There are no conflicts to declare.

## Supplementary Material

RA-015-D4RA07863B-s001

RA-015-D4RA07863B-s002
