# Supplementary material for: One-pot reaction of 3-vinylchromones, aromatic aldehydes, and ammonium acetate: an efficient approach to highly functionalized 1,6-dihydropyridine derivatives
Source: RSC Adv. 2025 Jan 27;15(4):2602–7. doi: 10.1039/d4ra07863b (PMC11770593; doi:10.1039/d4ra07863b)
Supplement: RA-015-D4RA07863B-s001 [file RA-015-D4RA07863B-s001.pdf]

Supplementary Information (SI) for RSC Advances.
This journal is © The Royal Society of Chemistry 2025

Supporting information

One-Pot Reaction of 3-Vinylchromones, Aromatic Aldehydes, and Ammonium Acetate: An Efficient Approach to Highly Functionalized 1,6-Dihydropyridine Derivatives

Behnaz Farajpour,[a, †] Marzie Kakaie,[a, †] Faiq H. S. Hussain,[b] Fataneh Rakaee,[a] Fatemeh Moradkhani,[c]

Morteza Shiri[a,*]

[a]Department of Organic Chemistry, Faculty of Chemistry, Alzahra University, Vanak, Tehran 1993893973, Iran.

[b]Medical Analysis Department, Applied Science Faculty, Tishk International University, Erbil 44001 Kurdistan Region, Iraq.

[c]Department of Medicinal Chemistry, Faculty of Pharmacy and Pharmaceutical Sciences Research Center, Tehran University of Medical Sciences, Tehran P94V+8MF, Iran.

[†]These authors contributed equally.

The Table of Contents

Title, author's names, address, and table of contents	S1
General Information	S2
General procedure for the synthesis of **3**	S2
Characterization data for all compounds	S3-S12
Copies of NMR spectra	S13-S52
The formation method of **3l** single crystals	S53
Crystal structure description of compound **3l**	S53-S54
References	S54

General Information

All reactions were monitored by thin-layer chromatography (TLC) on Merck silica gel 60 F254 plates. The temperatures were monitored using a mercury laboratory thermometer. Column chromatography purification was carried out on silica gel (63−200 mesh ASTM). Melting points were measured on an Electrothermal 9100 apparatus. ^{1}H NMR (300, 500, and 600 MHz) and ^{13}C{^{1}H} NMR (125 and 150 MHz) spectra were obtained using a Bruker spectrometer. NMR spectra were recorded at r.t. in DMSO-d_6. Chemical shifts are reported in parts per million (δ) downfield from an internal TMS reference. Standard abbreviations were used to indicate spin multiplicities (s = singlet, d = doublet, t = triplet, br = broad, m = multiplet, dd = doublet of doublets, td = triplet of doublets). Coupling constants (J values) are reported in hertz (Hz). High-resolution mass spectra (HRMS) were obtained on an Agilent HRMS-ESI/QTOF instrument. Purchased from Merck or Aldrich, all chemicals and solvents were used without further purification. 3-vinyl-4-chromones were synthesized according to the procedures reported in the literature.[1] Single crystals of compound **3l** were formed in the mixture of CH_2Cl_2 and *n*-hexane (1:1 v/v).

General procedure for the synthesis of 3.

To a solution of 3-vinyl-4-chromone **1** (1 mmol) and aromatic aldehyde **2** (1.5 mmol) in DMF (3.0 mL), ammonium acetate (4 mmol, 308 mg) was added. The reaction mixture was magnetically stirred at 100 °C in an oil bath for 5 h (monitored by TLC). Then, the resulting solution was concentrated under reduced pressure, and the residue was purified by column chromatography on silica gel using *n*-hexane/EtOAc (5:1 v/v) as the eluent.

Characterization data for all compounds.

(5-Benzoyl-6-phenyl-1,6-dihydropyridin-3-yl)(2-hydroxyphenyl)methanone (3a).

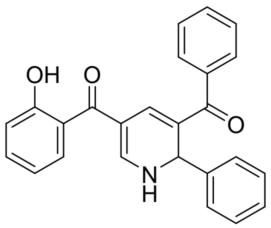

The reaction mixture was purified by column chromatography on silica gel using *n*-hexane/EtOAc (5:1 v/v) as the eluent to afford the product as a pale Yellow solid, (339 mg, 89% yield), mp 120-125 °C. ^{1}H NMR (600 MHz, DMSO-d_6) δ 10.21 (s, 1H, OH), 9.44 (brs, 1H, NH), 7.62 (d, 1H, J = 6.5 Hz), 7.55-7.47 (m, 7H), 7.43 (s, 1H), 7.40 (t, 2H, J = 8.0 Hz), 7.31 (t, 1H, J = 7.5 Hz), 7.27 (t, 1H, J = 7.6 Hz), 7.20 (d, 1H, J = 7.5 Hz), 6.89 (d, 1H, J = 8.2 Hz), 6.85 (t, 1H, J = 7.6 Hz), 5.96 (s, 1H). $^{13}C\{^{1}H\}$ NMR (150 MHz, DMSO-d_6) δ 193.9, 189.1, 155.9, 152.2, 143.6, 139.3, 138.1, 131.7, 131.3,129.7, 129.1, 128.7,128.5, 128.4, 127.2, 126.2, 121.0, 119.3, 116.7, 106.6, 54.7. HRMS (ESI-TOF) *m/z*: $[M + H]^+$ Calcd for $C_{25}H_{20}NO_3$ 382.1438; Found 382.1433.

(5-Benzoyl-6-(*p*-tolyl)-1,6-dihydropyridin-3-yl)(2-hydroxyphenyl)methanone (3b).

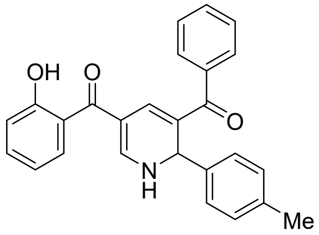

The reaction mixture was purified by column chromatography on silica gel using *n*-hexane/EtOAc (5:1 v/v) as the eluent to afford the product as a pale Yellow solid, (355 mg, 90% yield), mp 135-139 °C. ^{1}H NMR (600 MHz, DMSO-d_6) δ 10.19 (s, 1H, OH), 9.40 (brs, 1H, NH), 7.59 (d, 1H, J = 7.2 Hz), 7.54 (t, 1H, J = 7.1 Hz), 7.51-7.46 (m, 4H), 7.41 (s, 1H), 7.35 (d, 2H, J = 7.7 Hz), 7.26 (t ,1H, J = 7.7 Hz),7.20-7.17 (m, 3H), 6.88 (d, 1H, J = 8.2 Hz), 6.84 (t, 1H, J = 7.4 Hz), 5.91 (d, 1H, J = 2.5 Hz), 2.28 (s, 3H). $^{13}C\{^{1}H\}$ NMR (150 MHz, DMSO-d_6) δ 193.9, 189.1, 155.9, 152.1, 140.8, 139.3, 137.9, 137.6, 131.7, 131.3, 129.7, 129.6, 128.7, 128.5, 127.1, 126.2, 121.1, 119.3, 116.7, 106.5, 54.4, 21.1. HRMS (ESI-TOF) *m/z*: $[M + H]^+$ Calcd for $C_{26}H_{22}NO_3$ 396.1594; Found 396.1601.

(5-Benzoyl-6-(4-methoxyphenyl)-1,6-dihydropyridin-3-yl)(2-hydroxyphenyl)methanone (3c).

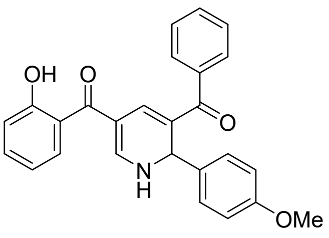

The reaction mixture was purified by column chromatography on silica gel using *n*-hexane/EtOAc (5:1 v/v) as the eluent to afford the product as a pale Yellow solid, (378 mg, 92% yield), mp 135-137 °C. ^{1}H NMR (600 MHz, DMSO-d_6) δ 10.20 (s, 1H, OH), 9.40 (d, 1H, J = 4.0 Hz, NH), 7.58 (d, 1H, J = 6.8 Hz), 7.54 (t, 1H, J = 7.0 Hz), 7.51-7.46 (m, 4H), 7.41-7.38 (m, 3H), 7.26 (t, 1H, J = 8.5 Hz), 7.19 (dd, 1H, J = 7.5 Hz, J = 1.3 Hz), 6.94 (d, 2H, J = 8.6 Hz), 6.88 (d, 1H, J = 8.1 Hz), 6.84 (t, 1H, J = 7.4 Hz), 5.89 (d, 1H, J = 2.9 Hz), 3.74 (s, 3H). ^{13}C{^{1}H} NMR (150 MHz, DMSO-d_6) δ 193.9, 189.1, 159.4, 155.9, 151.9, 139.3, 137.8, 136.0, 131.7, 131.3, 129.7, 128.7, 128.5, 126.2, 121.2, 119.3, 116.7, 114.4, 106.4, 55.5, 54.0. HRMS (ESI-TOF) *m/z*: $[M + H]^+$ Calcd for $C_{26}H_{22}NO_4$ 412.1543; Found 412.1549.

(5-Benzoyl-6-(3-methoxyphenyl)-1,6-dihydropyridin-3-yl)(2-hydroxyphenyl)methanone (3d).

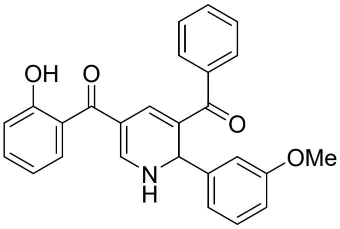

The reaction mixture was purified by column chromatography on silica gel using *n*-hexane/EtOAc (5:1 v/v) as the eluent to afford the product as a pale Yellow solid, (361 mg, 88% yield), mp 205-208 °C. ^{1}H NMR (600 MHz, DMSO-d_6) δ 10.18 (s, 1H, OH), 9.42 (dd, 1H, J = 6.7 Hz, J = 3.0 Hz, NH), 7.61 (d, 1H, J = 7.0 Hz), 7.56-7.47 (m, 5H), 7.42 (s, 1H), 7.31 (t, 1H, J = 7.9 Hz), 7.26 (td, 1H, J = 7.2 Hz, J = 1.6 Hz), 7.18 (dd, 1H, J = 7.6 Hz, J = 1.6 Hz), 7.04 (d, 1H, J = 7.7 Hz), 7.01-7.00 (m, 1H), 6.89 (d, 2H, J = 8.1 Hz), 6.84 (t, 1H, J = 7.5 Hz), 5.94 (d, 1H, J = 3.3 Hz), 3.75 (s, 3H). ^{13}C{^{1}H} NMR (150 MHz, DMSO-d_6) δ 193.9, 189.1, 159.9, 155.8, 152.1, 145.1, 139.2, 138.2, 131.7, 131.4, 130.3, 129.7, 128.7, 128.5, 126.3, 120.8, 119.3, 116.7, 113.6, 112.9, 106.7, 55.5, 54.5. HRMS (ESI-TOF) *m/z*: $[M + H]^+$ Calcd for $C_{26}H_{22}NO_4$ 412.1543; Found 412.1548.

(5-Benzoyl-6-(4-(dimethylamino)phenyl)-1,6-dihydropyridin-3-yl)(2-hydroxyphenyl)methanone (3e).

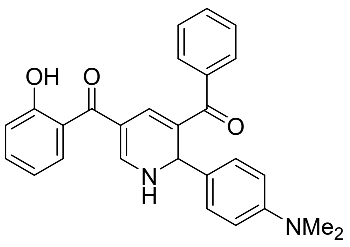

The reaction mixture was purified by column chromatography on silica gel using *n*-hexane/EtOAc (5:1 v/v) as the eluent to afford the product as a pale Yellow solid, (394 mg, 93% yield), mp 125-127 °C. ^{1}H NMR (600 MHz, DMSO-d_6) δ 10.23 (s, 1H, OH), 9.38 (d, 1H, *J* = 4.1 Hz, NH), 7.56-7.46 (m, 6H), 7.38 (s, 1H), 7.28-7.25 (m, 3H), 7.19 (dd, 1H, *J* = 7.5 Hz, *J* = 1.4 Hz), 6.88 (d, 1H, *J* = 8.0 Hz), 6.84 (t, 1H, *J* = 7.3 Hz), 6.71 (d, 2H, *J* = 8.7 Hz), 5.81 (d, 1H, *J* = 3.0 Hz), 2.87 (s, 6H). ^{13}C{^{1}H} NMR (150 MHz, DMSO-d_6) δ 194.0, 189.0, 156.0, 151.7, 150.6, 139.4, 137.4, 131.7, 131.6, 131.3, 129.7, 128.7, 128.5, 128.0, 126.2, 121.5, 119.2, 116.7, 112.7, 106.3, 54.2, 40.5. HRMS (ESI-TOF) *m/z*: [M + H]$^+$ Calcd for $C_{27}H_{25}N_2O_3$ 425.1860; Found 425.1866.

(5-Benzoyl-6-(4-bromophenyl)-1,6-dihydropyridin-3-yl)(2-hydroxyphenyl)methanone (3f).

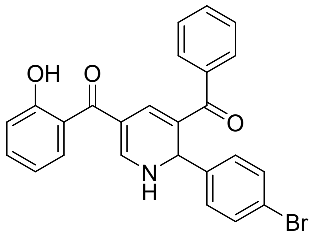

The reaction mixture was purified by column chromatography on silica gel using *n*-hexane/EtOAc (5:1 v/v) as the eluent to afford the product as a pale Yellow solid, (399 mg, 87% yield), mp 132-134 °C. ^{1}H NMR (500 MHz, DMSO-d_6) δ 10.16 (s, 1H, OH), 9.40 (dd, 1H, *J* = 6.6 Hz, *J* = 2.9 Hz, NH), 7.61 (d, 1H, *J* = 7.0 Hz), 7.57 (d, 2H, *J* = 8.4 Hz), 7.53 (t, 1H, *J* = 7.0 Hz), 7.49-7.39 (m, 7H), 7.25 (t, 1H, *J* = 8.2 Hz), 7.18 (d, 1H, *J* = 7.6 Hz), 6.87 (d, 1H, *J* = 8.2 Hz), 6.83 (t, 1H, *J* = 8.1 Hz), 5.93 (d, 1H, *J* = 3.0 Hz), ^{13}C{^{1}H} NMR (125 MHz, DMSO-d_6) δ 193.3, 188.7, 155.5, 151.8, 142.3, 138.7, 137.8, 131.5, 131.3, 130.8, 129.3, 128.9, 128.2, 128.0, 125.6, 121.0, 120.1, 118.8, 116.2, 106.3, 53.6. HRMS (ESI-TOF) *m/z*: [M + H]$^+$ Calcd for $C_{25}H_{19}BrNO_3$ 460.0543; Found 460.0540.

(5-Benzoyl-6-(3-romophenyl)-1,6-dihydropyridin-3-yl)(2-hydroxyphenyl)methanone (3g).

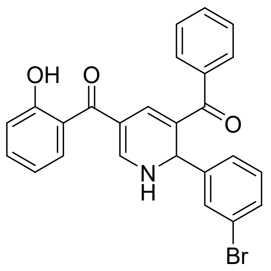

The reaction mixture was purified by column chromatography on silica gel using *n*-hexane/EtOAc (5:1 v/v) as the eluent to afford the product as a pale Yellow solid, (380 mg, 83% yield), mp 128-130 °C. 1H NMR (500 MHz, DMSO-d_6) δ 10.13 (s, 1H, OH), 9.39 (brs, 1H, NH), 7.63-7.61 (m, 2H), 7.54-7.45 (m, 8H), 7.35 (t, 1H, J = 7.8 Hz), 7.25 (t, 1H, J = 7.4 Hz), 7.18 (d, 1H, J = 7.5 Hz), 6.88 (d, 1H, J = 8.2 Hz), 6.83 (t, 1H, J = 7.4 Hz), 5.96 (s, 1H). $^{13}C\{^1H\}$ NMR (125 MHz, DMSO-d_6) δ 193.2, 188.7, 155.3, 151.8, 145.5, 138.6, 138.2, 131.3, 131.0, 130.9, 130.7, 129.5, 129.2, 128.2, 128.0, 125.8, 125.7, 121.8, 119.8, 118.8, 116.2, 106.3, 53.7. HRMS (ESI-TOF) *m/z*: $[M + H]^+$ Calcd for $C_{25}H_{19}BrNO_3$ 460.0543; Found 460.0538.

(5-benzoyl-6-(4-chlorophenyl)-1,6-dihydropyridin-3-yl)(2-hydroxyphenyl)methanone (3h).

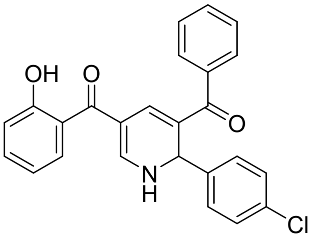

The reaction mixture was purified by column chromatography on silica gel using *n*-hexane/EtOAc (5:1 v/v) as the eluent to afford the product as a pale Yellow solid, (352 mg, 85% yield), mp 117-120 °C. ^1H NMR (600 MHz, DMSO-d_6) δ 10.17 (s, 1H, OH), 9.40 (d, 1H, J = 2.9 Hz, NH), 7.63 (d, 1H, J = 6.3 Hz), 7.54 (t, 1H, J = 7.0 Hz), 7.51-7.45 (m, 9H), 7.27 (t, 1H, J = 8.3 Hz), 7.20 (d, 1H, J = 7.4 Hz), 6.88 (d, 1H, J = 8.1 Hz), 6.85 (t, 1H, J = 7.4 Hz), 5.97 (d, 1H, J = 1.7 Hz). $^{13}C\{^1H\}$ NMR (150 MHz, DMSO-d_6) δ 193.8, 189.2, 155.9, 152.3, 142.5, 139.2, 138.4, 133.0, 131.8, 131.4, 129.8, 129.1, 129.1, 128.7, 128.5, 126.2, 120.6, 119.3, 116.7, 106.8, 54.0. HRMS (ESI-TOF) *m/z*: $[M + H]^+$ Calcd for $C_{25}H_{19}ClNO_3$ 416.1048; Found 416.1053.

(5-(2-Hydroxybenzoyl)-2-phenyl-1,2-dihydropyridin-3-yl)(*p*-tolyl)methanone (3i).

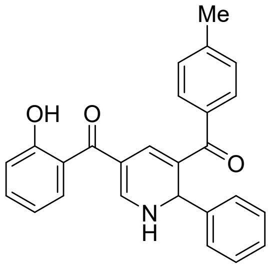

The reaction mixture was purified by column chromatography on silica gel using *n*-hexane/EtOAc (5:1 v/v) as the eluent to afford the product as a pale Yellow solid, (339 mg, 86% yield), mp 107-110 °C. ^{1}H NMR (600 MHz, DMSO-d_6) δ 10.21 (s, 1H, OH), 9.38 (d, 1H, J = 3.6 Hz, NH), 7.58 (d, 1H, J = 6.8 Hz), 7.46 (d, 2H, J = 7.5 Hz), 7.43 (s, 1H), 7.42 (d, 2H, J = 7.9 Hz), 7.39 (t, 2H, J = 7.7 Hz), 7.32-7.26 (m, 4H), 7.19 (d, 1H, J = 7.5 Hz), 6.88 (d, 1H, J = 8.1 Hz), 6.85 (t, 1H, J = 7.4 Hz), 5.95 (d, 1H, J = 2.9 Hz), 2.36 (s, 3H). ^{13}C{^{1}H} NMR (150 MHz, DMSO-d_6) δ 193.8, 189.1, 155.9, 152.2, 143.6, 141.4, 137.4, 136.5, 131.7, 129.7, 129.3, 129.1, 128.7, 128.4, 127.2, 126.2, 121.2, 119.3, 116.7, 106.6, 54.7, 21.4. HRMS (ESI-TOF) *m/z*: [M + H]$^+$ Calcd for $C_{26}H_{22}NO_3$ 396.1594; Found 396.1600.

(5-(4-Bromobenzoyl)-6-phenyl-1,6-dihydropyridin-3-yl)(2-hydroxyphenyl)methanone (3j).

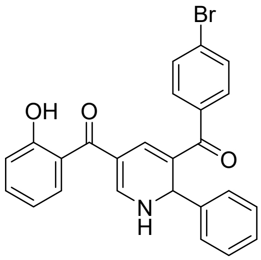

The reaction mixture was purified by column chromatography on silica gel using *n*-hexane/EtOAc (5:1 v/v) as the eluent to afford the product as a pale Yellow solid, (403 mg, 88% yield), mp 103-106 °C. ^{1}H NMR (300 MHz, DMSO-d_6) δ 10.19 (s, 1H, OH), 9.46 (brs, 1H, NH), 7.68 (d, 2H, J = 8.3 Hz), 7.62 (d, 1H, J = 7.0 Hz), 7.46-7.25 (m, 9H), 7.19 (d, 1H, J = 7.3 Hz), 6.89-6.82 (m, 2H), 5.93 (d, 1H, J = 2.9 Hz). ^{13}C{^{1}H} NMR (150 MHz, DMSO-d_6) δ 192.7, 189.1, 155.8, 152.4, 143.5, 138.5, 138.3, 131.8, 130.6, 129.8, 129.2, 128.4, 127.2, 126.2, 124.9, 120.6, 119.4, 116.7, 106.7, 54.6. HRMS (ESI-TOF) *m/z*: [M + H]$^+$ Calcd for $C_{25}H_{19}BrNO_3$ 460.0543; Found 460.0542.

(5-(4-Chlorobenzoyl)-6-phenyl-1,6-dihydropyridin-3-yl)(2-hydroxyphenyl)methanone (3k).

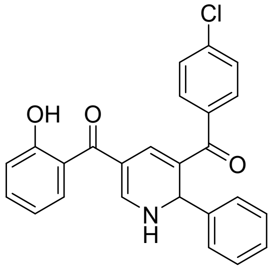

The reaction mixture was purified by column chromatography on silica gel using *n*-hexane/EtOAc (5:1 v/v) as the eluent to afford the product as a pale Yellow solid, (373 mg, 90% yield), mp 117-120 °C. ^{1}H NMR (600 MHz, DMSO-d_6) δ 10.21 (s, 1H, OH), 9.48 (d, 1H, J = 3.9 Hz, NH), 7.63 (d, 1H, J = 6.9 Hz), 7.55-7.52 (m, 4H), 7.47 (d, 2H, J = 7.3 Hz), 7.41 (s, 1H), 7.39 (t, 2H, J = 7.5 Hz), 7.31 (t, 1H, J = 7.3 Hz), 7.28 (t, 1H, J = 8.4 Hz), 7.20 (dd, 1H, J = 7.5 Hz, J = 1.3 Hz), 6.89 (d, 1H, J = 8.1 Hz), 6.85 (t, 1H, J = 7.3 Hz), 5.95 (d, 1H, J = 3.1 Hz). ^{13}C{^{1}H} NMR (150 MHz, DMSO-d_6) δ 192.6, 189.1, 155.8, 152.3, 143.5, 138.4, 138.0, 136.1, 131.8, 130.4, 129.8, 129.2, 128.9, 128.4, 127.2, 126.2, 120.7, 119.4, 116.7, 106.6, 54.7. HRMS (ESI-TOF) *m/z*: [M + H]$^+$ Calcd for $C_{25}H_{19}ClNO_3$ 416.1048; Found 416.1057.

(5-(4-Chlorobenzoyl)-6-(2-chlorophenyl)-1,6-dihydropyridin-3-yl)(2-hydroxyphenyl)methanone (3l).

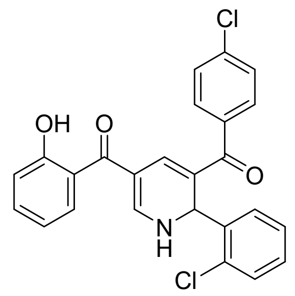

The reaction mixture was purified by column chromatography on silica gel using *n*-hexane/EtOAc (5:1 v/v) as the eluent to afford the product as a pale Yellow solid, (408 mg, 91% yield), mp 223-226 °C. ^{1}H NMR (600 MHz, DMSO-d_6) δ 10.17 (s, 1H, OH), 9.48 (s, 1H, NH), 7.62 (dd, 1H, J = 7.7 Hz, J = 1.7 Hz), 7.60-7.55 (m, 5H), 7.51 (dd, 1H, J = 7.8 Hz, J = 0.8 Hz), 7.45 (s, 1H), 7.41 (t, 1H, J = 7.4 Hz), 7.36 (td, 1H, J = 7.7 Hz, J = 1.5 Hz), 7.28 (td, 1H, J = 8.3 Hz, J = 1.7 Hz), 7.20 (dd, 1H, J = 7.6 Hz, J = 1.7 Hz), 6.89 (d, 1H, J = 8.2 Hz), 6.85 (t, 1H, J = 7.5 Hz), 6.34 (s, 1H). ^{13}C{^{1}H} NMR (150 MHz, DMSO-d_6) δ 192.1, 189.0, 155.7, 151.9, 139.8, 139.7, 137.7, 136.2, 131.8, 131.7, 130.5, 130.4, 130.1, 130.0, 129.7, 128.9, 128.6, 126.3, 119.6, 119.4, 116.6, 106.6, 52.0. HRMS (ESI-TOF) *m/z*: [M + H]$^+$ Calcd for $C_{25}H_{18}Cl_2NO_3$ 450.0658; Found 450.0671.

(5-(2-Hydroxybenzoyl)-2-phenyl-1,2-dihydropyridin-3-yl)(4-nitrophenyl)methanone (3m).

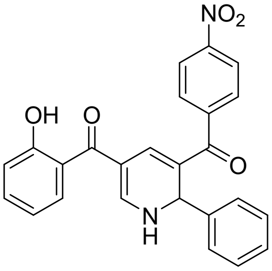

The reaction mixture was purified by column chromatography on silica gel using *n*-hexane/EtOAc (5:1 v/v) as the eluent to afford the product as a pale Yellow solid, (392 mg, 92% yield), mp 126-129 °C. ^{1}H NMR (600 MHz, DMSO-d_6) δ 10.18 (s, 1H, OH), 9.58 (dd, 1H, J = 6.8 Hz, J = 2.8 Hz, NH), 8.31 (d, 2H, J = 8.7 Hz), 7.73 (d, 2H, J = 8.7 Hz), 7.66 (d, 1H, J = 7.1 Hz), 7.48 (d, 2H, J = 7.3 Hz), 7.42-7.39 (m, 3H), 7.33 (t, 1H, J = 7.3 Hz), 7.27 (td, 1H, J = 7.8 Hz, J = 1.7 Hz), 7.18 (dd, 1H, J = 8.0 Hz, J = 1.6 Hz), 6.88 (d, 1H, J = 8.1 Hz), 6.85 (t, 1H, J = 7.5 Hz), 5.97 (d, 1H, J = 3.3 Hz). ^{13}C{^{1}H} NMR (150 MHz, DMSO-d_6) δ 192.0 189.0, 155.7, 152.8, 148.9, 145.2, 143.3, 139.7, 131.8, 129.8, 129.7, 129.2, 128.5, 127.2, 126.3, 124.0, 120.4, 119.4, 116.6, 106.9, 54.5. HRMS (ESI-TOF) *m/z*: [M + H]$^+$ Calcd for $C_{25}H_{19}N_2O_5$ 427.1288; Found 427.1298.

1-(5-(2-hydroxybenzoyl)-2-phenyl-1,2-dihydropyridin-3-yl)ethan-1-one (3n).

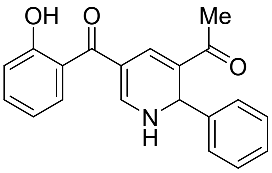

The reaction mixture was purified by column chromatography on silica gel using *n*-hexane/EtOAc (5:1 v/v) as the eluent to afford the product as a pale Yellow solid, (239 mg, 75% yield), mp 126-128 °C. ^{1}H NMR (600 MHz, DMSO-d_6) δ 10.22 (s, 1H, OH), 9.22 (dd, 1H, J = 6.5 Hz, J = 2.8 Hz, NH), 7.80 (d, 1H, J = 1.0 Hz), 7.51 (d, 1H, J = 7.0 Hz), 7.36-7.27 (m, 6H), 7.20 (dd, 1H, J = 7.6 Hz, J = 1.5 Hz), 6.89 (d, 1H, J = 8.2 Hz), 6.87 (td, 1H, J = 7.5 Hz, J = 0.8 Hz), 5.72 (d, 1H, J = 3.3 Hz), 2.26 (s, 3H). ^{13}C{^{1}H} NMR (150 MHz, DMSO-d_6) δ 195.2, 189.1, 156.0, 152.6, 143.7, 134.1, 131.7, 129.7, 128.9, 128.1, 127.1, 126.3, 122.1, 119.3, 116.7, 106.5, 53.9, 25.2. HRMS (ESI-TOF) *m/z*: [M + H]$^+$ Calcd for $C_{20}H_{18}NO_3$ 320.1281; Found 320.1278.

(5-Benzoyl-6-phenyl-1,6-dihydropyridin-3-yl)(2-hydroxy-5-methylphenyl)methanone (3o).

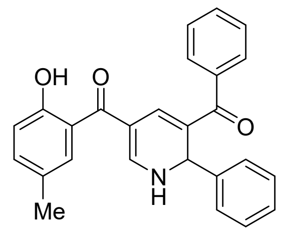

The reaction mixture was purified by column chromatography on silica gel using *n*-hexane/EtOAc (5:1 v/v) as the eluent to afford the product as a pale Yellow solid, (331 mg, 84% yield), mp 135-138 °C. ^{1}H NMR (600 MHz, $CDCl_3$) δ 11.24 (s, 1H, OH), 7.76 (d, 1H, J = 7.2 Hz), 7.62 (d, 1H, J = 1.2 Hz), 7.58 (d, 2H, J = 7.8 Hz), 7.52 (d, 2H, J = 7.3 Hz), 7.49 (t, 1H, J = 7.4 Hz), 7.43-7.39 (m, 4H), 7.35 (t, 1H, J = 7.3 Hz), 7.23-7.21 (m, 2H), 6.91 (d, 1H, J = 8.2 Hz), 6.59 (brs, 1H, NH), 6.15 (d, 1H, J = 3.0 Hz), 2.30 (s, 3H). $^{13}C\{^{1}H\}$ NMR (150 MHz, $CDCl_3$) δ 194.7, 192.3, 159.0, 150.1, 142.5, 138.4, 136.9, 135.1, 131.4, 130.1, 129.1, 128.6, 128.5, 128.3, 127.3, 126.7, 123.0, 119.7, 117.9, 106.4, 55.7, 20.70. HRMS (ESI-TOF) *m/z*: $[M + H]^+$ Calcd for $C_{26}H_{22}NO_3$ 396.1594; Found 396.1598.

(5-(2-Hydroxy-5-methylbenzoyl)-2-phenyl-1,2-dihydropyridin-3-yl)(*p*-tolyl)methanone (3p).

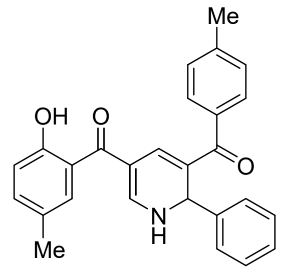

The reaction mixture was purified by column chromatography on silica gel using *n*-hexane/EtOAc (5:1 v/v) as the eluent to afford the product as a pale Yellow solid, (335 mg, 82% yield), mp 168-172 °C. ^{1}H NMR (600 MHz, $CDCl_3$) δ 11.25 (s, 1H, OH), 7.71 (d, 1H, J = 7.0 Hz), 7.61 (s, 1H), 7.57 (d, 2H, J = 7.2 Hz), 7.48 (t, 1H, J = 7.4 Hz), 7.41-7.37 (m, 4H), 7.21-7.18 (m, 4H), 6.90 (d, 1H, J = 8.9 Hz), 6.87 (d, 1H, J = 3.6 Hz, NH), 6.07 (d, 1H, J = 2.8 Hz), 2.35 (s, 3H), 2.29 (s, 3H). $^{13}C\{^{1}H\}$ NMR (150 MHz, $CDCl_3$) δ 194.9, 192.2, 158.9, 150.3, 139.7, 138.5, 137.1, 135.1, 131.4, 130.1, 129.8, 128.5, 128.3, 127.3, 126.7, 122.9, 119.7, 117.9, 106.2, 55.4, 21.1, 20.6. HRMS (ESI-TOF) *m/z*: $[M + H]^+$ Calcd for $C_{27}H_{24}NO_3$ 410.1751; Found 410.1745.

(5-Benzoyl-6-phenyl-1,6-dihydropyridin-3-yl)(5-chloro-2-hydroxyphenyl)methanone (3q).

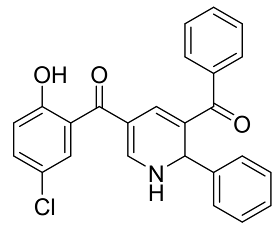

The reaction mixture was purified by column chromatography on silica gel using *n*-hexane/EtOAc (5:1 v/v) as the eluent to afford the product as a pale Yellow solid, (356 mg, 86% yield), mp 121-123 °C. ^{1}H NMR (600 MHz, DMSO-d_6) δ 10.23 (s, 1H, OH), 9.45 (brs, 1H, NH), 7.59 (brs, 1H), 7.54 (t, 1H, J = 6.6 Hz), 7.49-7.46 (m, 6H), 7.40-7.37 (m, 3H), 7.31 (t, 1H, J = 7.2 Hz), 7.28 (dd, 1H, J = 8.7 Hz, J = 2.5 Hz), 7.15 (d, 1H, J = 2.5 Hz), 6.89 (d, 1H, J = 8.7 Hz), 5.95 (d, 1H, J = 1.2 Hz). ^{13}C{^{1}H} NMR (150 MHz, DMSO-d_6) δ 193.9, 186.9, 153.9, 152.4, 143.6, 139.2, 131.4, 130.8, 129.5, 129.3, 129.1, 128.9, 128.8, 128.7, 128.5, 128.4, 127.2, 122.9, 118.3, 106.5, 54.8. HRMS (ESI-TOF) *m/z*: [M + H]$^+$ Calcd for $C_{25}H_{19}ClNO_3$ 416.1048; Found 416.1048.

(5-Benzoyl-6-(2-chlorophenyl)-1,6-dihydropyridin-3-yl)(5-chloro-2-hydroxyphenyl)methanone (3r).

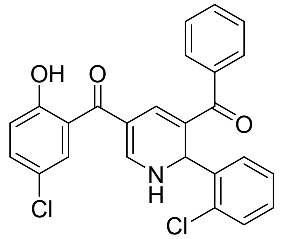

The reaction mixture was purified by column chromatography on silica gel using *n*-hexane/EtOAc (5:1 v/v) as the eluent to afford the product as a pale Yellow solid, (381 mg, 85% yield), mp 170-172 °C. ^{1}H NMR (600 MHz, DMSO-d_6) δ 10.27 (brs, 1H, OH), 9.49 (brs, 1H, NH), 7.61 (d, 1H, J = 7.6 Hz), 7.57-7.48 (m, 7H), 7.41 (t, 2H, J = 7.3 Hz), 7.36 (t, 1H, J = 7.4 Hz), 7.28 (dd, 1H, J = 8.7 Hz, J = 2.2 Hz), 7.15 (d, 1H, J = 2.0 Hz), 6.90 (d, 1H, J = 8.7 Hz), 6.33 (s, 1H). ^{13}C{^{1}H} NMR (150 MHz, DMSO-d_6) δ 193.4, 186.9, 153.9, 139.9, 139.0, 134.9, 133.8, 131.7, 131.5, 130.9, 130.3, 130.1, 130.0, 128.8, 128.8, 128.6, 128.5, 122.9, 118.3, 110.9, 106.4, 52.1. HRMS (ESI-TOF) *m/z*: [M + H]$^+$ Calcd for $C_{25}H_{18}Cl_2NO_3$ 450.0658; Found 450.0662.

(6-(1*H*-indol-3-yl)-5-(4-methylbenzoyl)-1,6-dihydropyridin-3-yl)(2-hydroxyphenyl)methanone (3s).

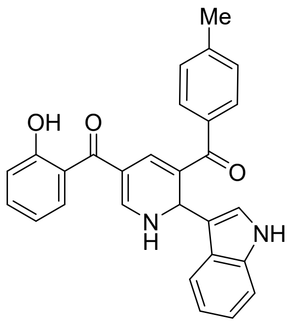

The reaction mixture was purified by column chromatography on silica gel using *n*-hexane/EtOAc (5:1 v/v) as the eluent to afford the product as a pale Yellow solid, (386 mg, 89% yield), mp 118-120 °C. ^{1}H NMR (600 MHz, DMSO-d_6) δ 11.07 (d, 1H, J = 1.8 Hz, NH), 10.28 (s, 1H, OH), 9.32 (d, 1H, J = 4.4 Hz, NH), 7.83 (d, 1H, J = 7.9 Hz), 7.50 (d, 1H, J = 6.9 Hz), 7.42-7.37 (m, 4H), 7.34 (d, 1H, J = 2.5 Hz), 7.28-7.25 (m, 3H), 7.21 (dd, 1H, J = 7.6 Hz, J = 1.5 Hz), 7.11 (td, 1H, J = 7.5 Hz, J = 1.0 Hz), 7.06 (td, 1H, J = 7.5 Hz, J = 0.8 Hz), 6.89 (d, 1H, J = 7.8 Hz), 6.84 (td, 1H, J = 7.1 Hz, J = 0.7 Hz), 6.26 (d, 1H, J = 3.0 Hz), 2.35 (s, 3H). ^{13}C{^{1}H} NMR (150 MHz, DMSO-d_6) δ 193.9, 189.2, 156.1, 151.6, 141.4, 137.0, 136.6, 136.3, 131.7, 129.7, 129.2, 128.8, 126.2, 125.5, 124.5, 121.7, 121.0, 119.7, 119.4, 119.2, 117.7, 116.7, 112.1, 106.2, 48.0, 21.4. HRMS (ESI-TOF) *m/z*: [M + H]$^+$ Calcd for $C_{28}H_{23}N_2O_3$ 435.1703; Found 435.1706.

(5-(4-Chlorobenzoyl)-6-(furan-2-yl)-1,6-dihydropyridin-3-yl)(2-hydroxyphenyl)methanone (3t).

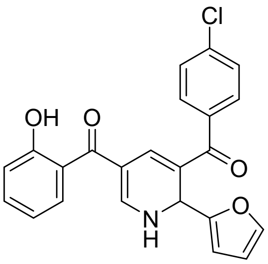

The reaction mixture was purified by column chromatography on silica gel using *n*-hexane/EtOAc (5:1 v/v) as the eluent to afford the product as a pale Yellow solid, (324 mg, 80% yield), mp 156-158 °C. ^{1}H NMR (500 MHz, DMSO-d_6) δ 10.19 (s, 1H, OH), 9.51 (dd, 1H, J = 6.1 Hz, J = 3.0 Hz, NH), 7.64-7.63 (m, 1H), 7.60-7.54 (m, 5H), 7.41 (s, 1H), 7.26 (td, 1H, J = 7.8 Hz, J = 1.5 Hz), 7.16 (dd, 1H, J = 7.5 Hz, J = 1.3 Hz), 6.87 (d, 1H, J = 8.2 Hz), 6.83 (t, 1H, J = 7.6 Hz), 6.41-6.40 (m, 1H), 6.34 (d, 1H, J = 3.2 Hz), 6.01 (d, 1H, J = 3.0 Hz). ^{13}C{^{1}H} NMR (125 MHz, DMSO-d_6) δ 191.7, 189.0, 155.6, 154.3, 151.6, 142.8, 138.4, 137.3, 135.7, 131.4, 130.0, 129.2, 128.4, 125.4, 118.8, 117.2, 116.3, 110.7, 107.1, 106.8, 47.5. HRMS (ESI-TOF) *m/z*: [M + H]$^+$ Calcd for $C_{23}H_{17}ClNO_4$ 406.0841; Found 406.0833.

Copies of NMR spectra

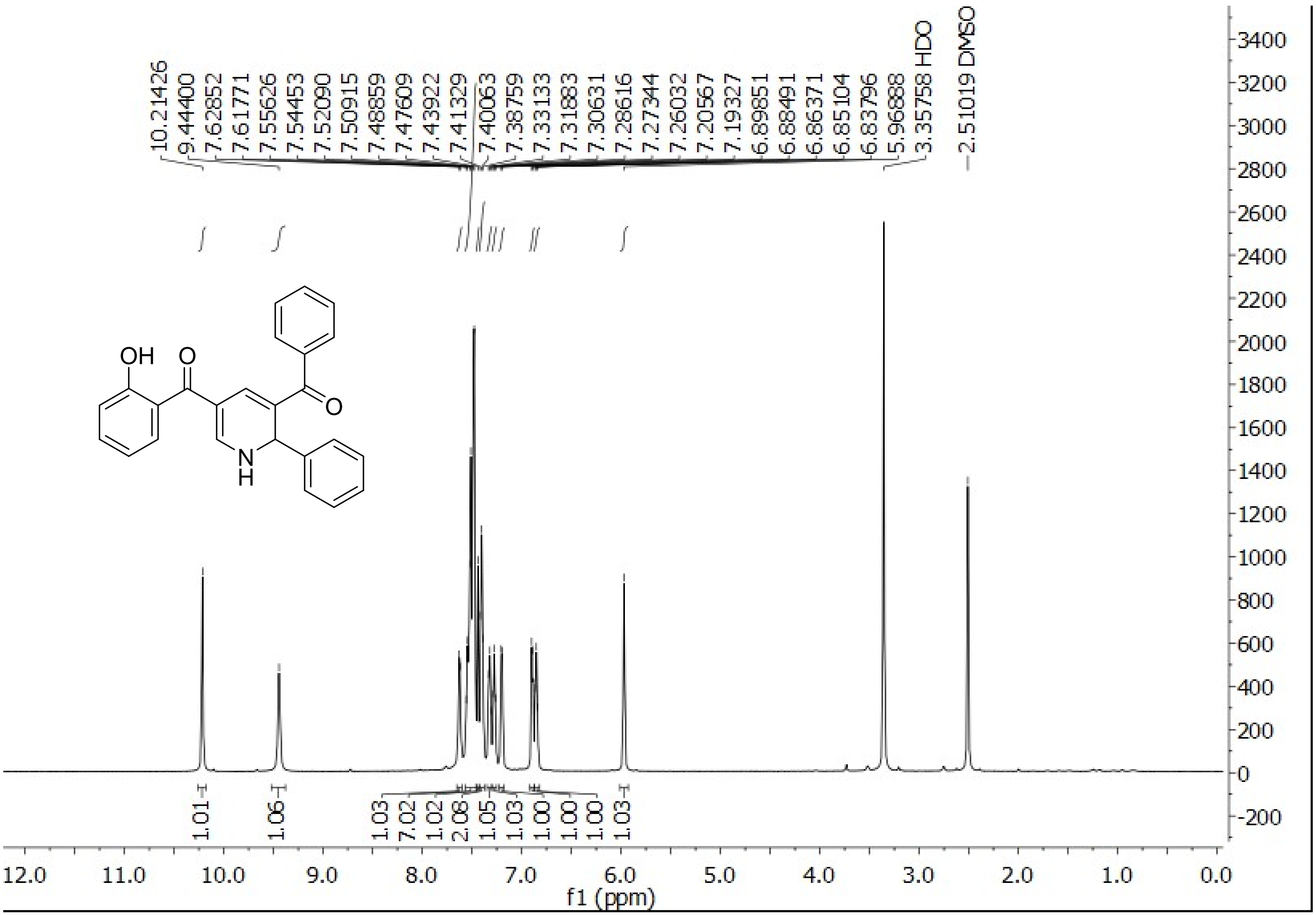

^{1}H NMR spectrum of **3a** (600 MHz, DMSO-d_6)

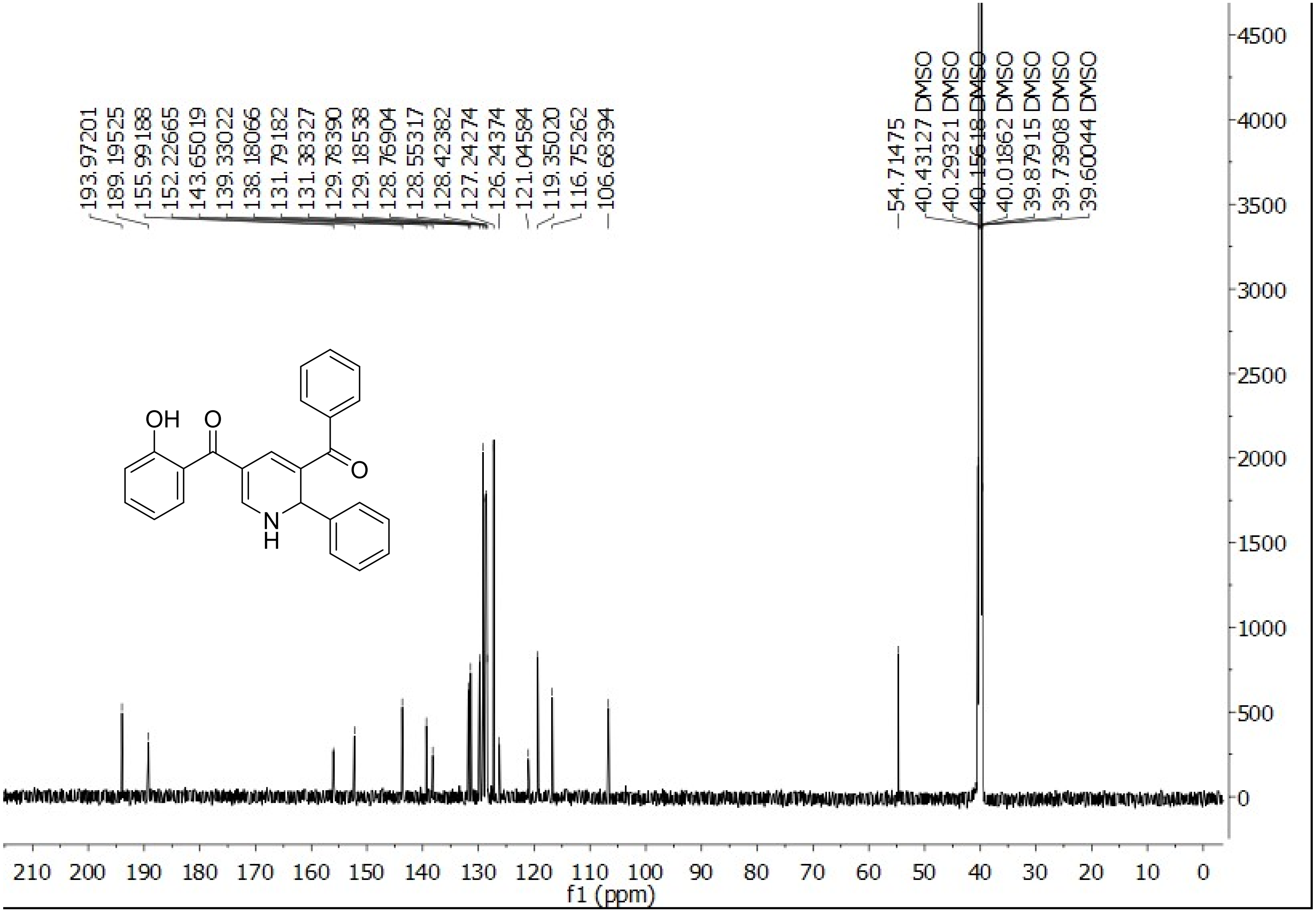

$^{13}C\{^{1}H\}$ NMR spectrum of **3a** (150 MHz, DMSO-d_6)

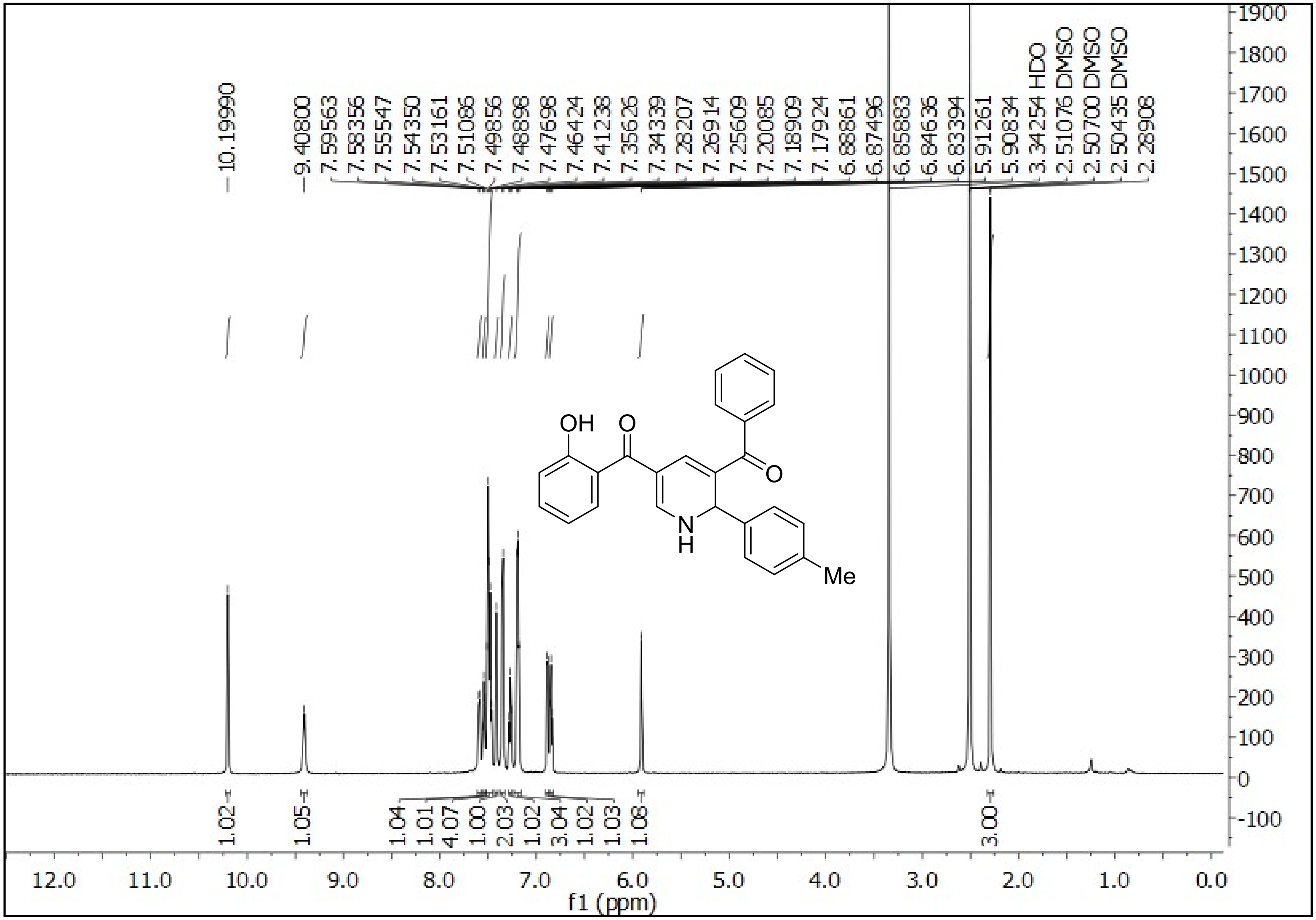

^{1}H NMR spectrum of **3b** (600 MHz, DMSO-d_6)

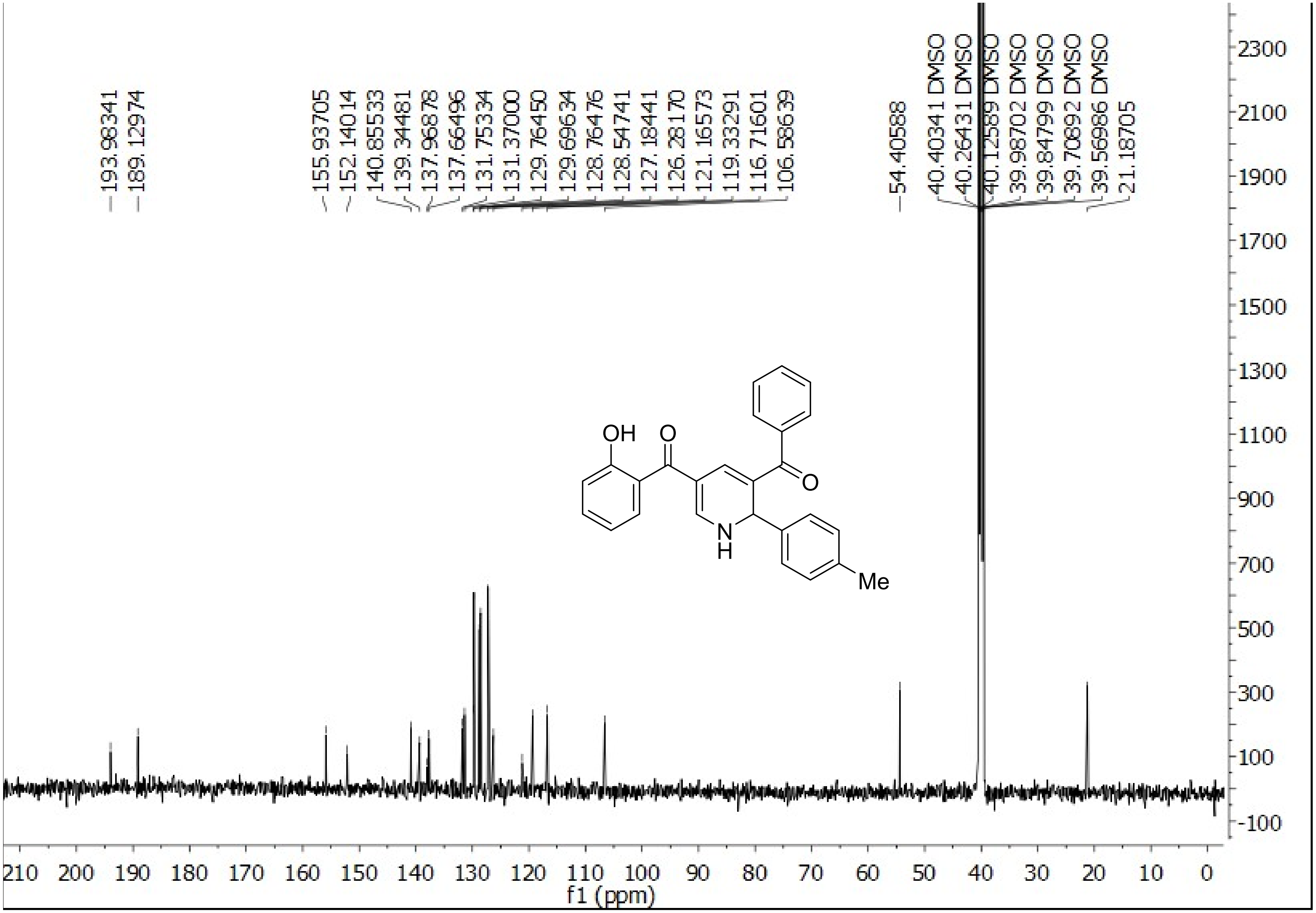

$^{13}C\{^1H\}$ NMR spectrum of **3b** (150 MHz, DMSO-d_6)

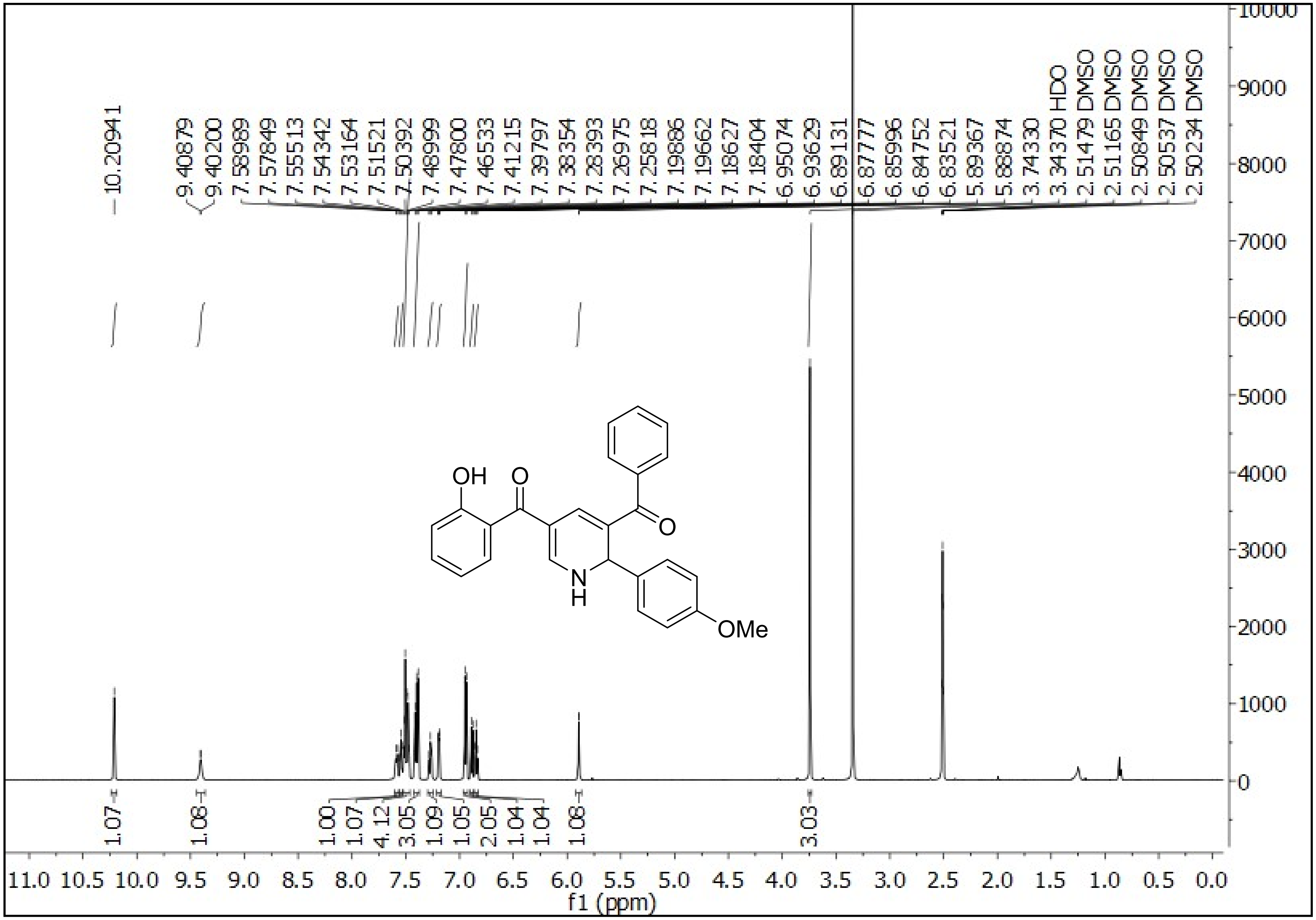

^{1}H NMR spectrum of **3c** (600 MHz, DMSO-d_6)

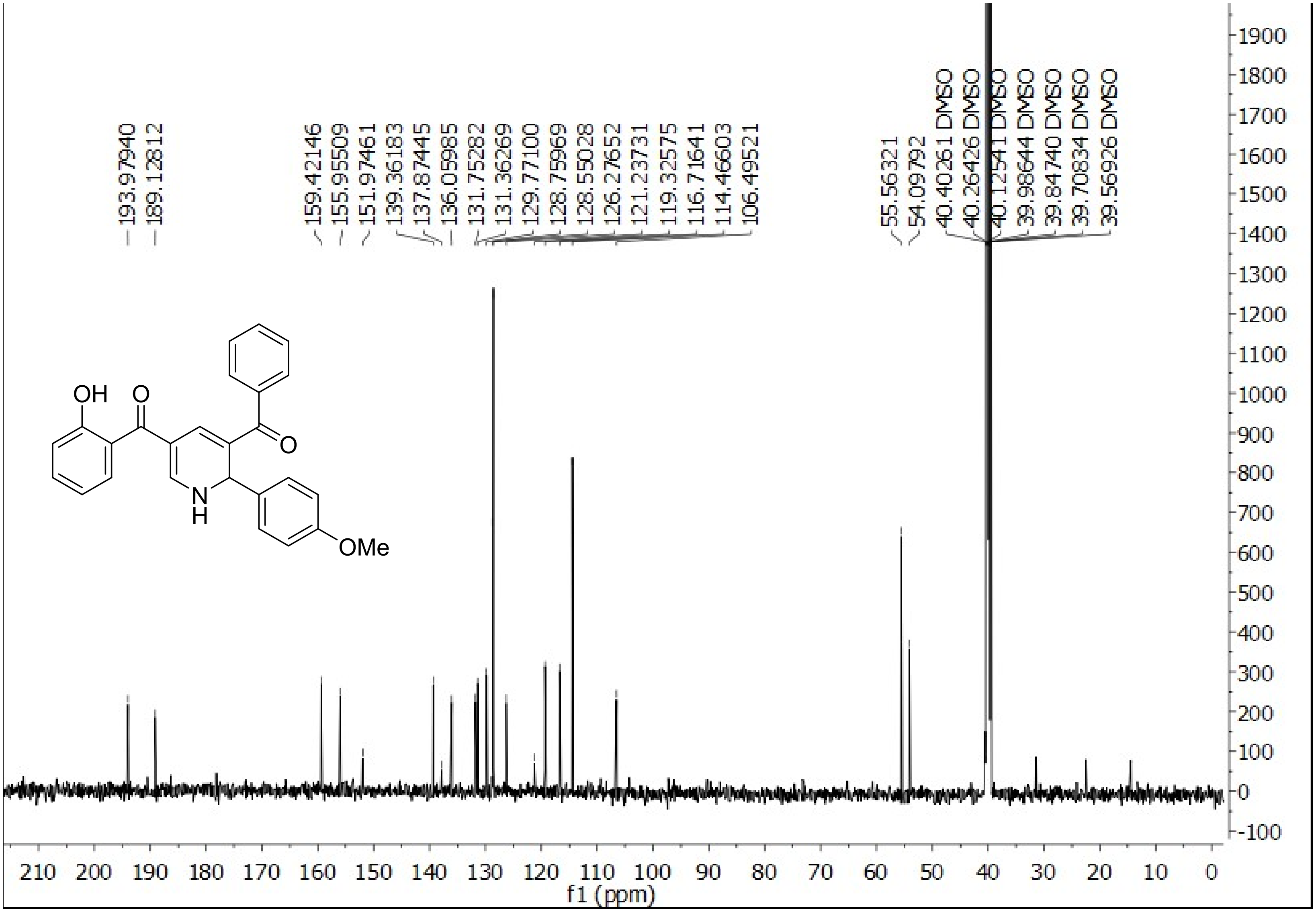

$^{13}C\{^1H\}$ NMR spectrum of **3c** (150 MHz, DMSO-d_6)

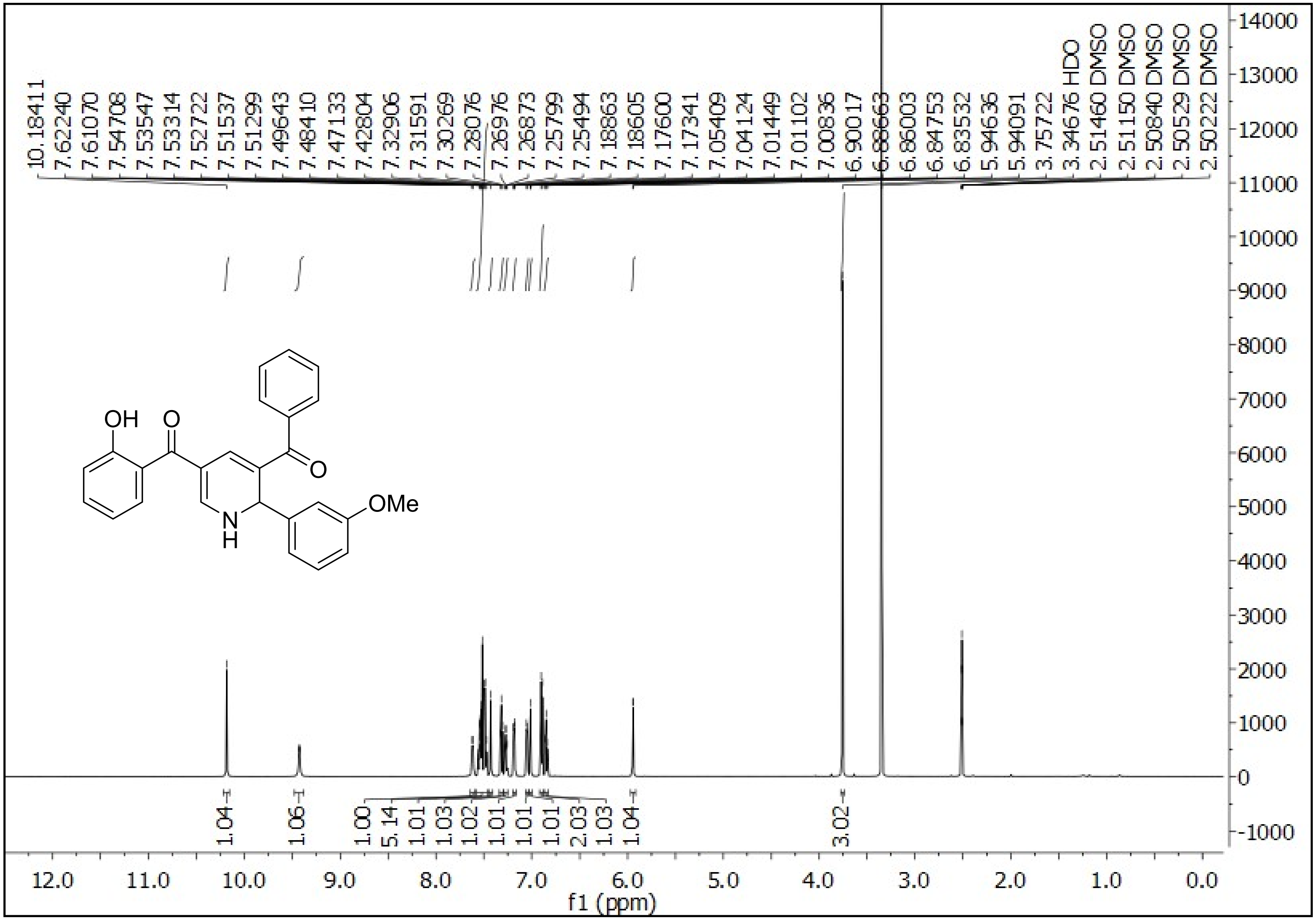

^{1}H NMR spectrum of **3d** (600 MHz, DMSO-d_6)

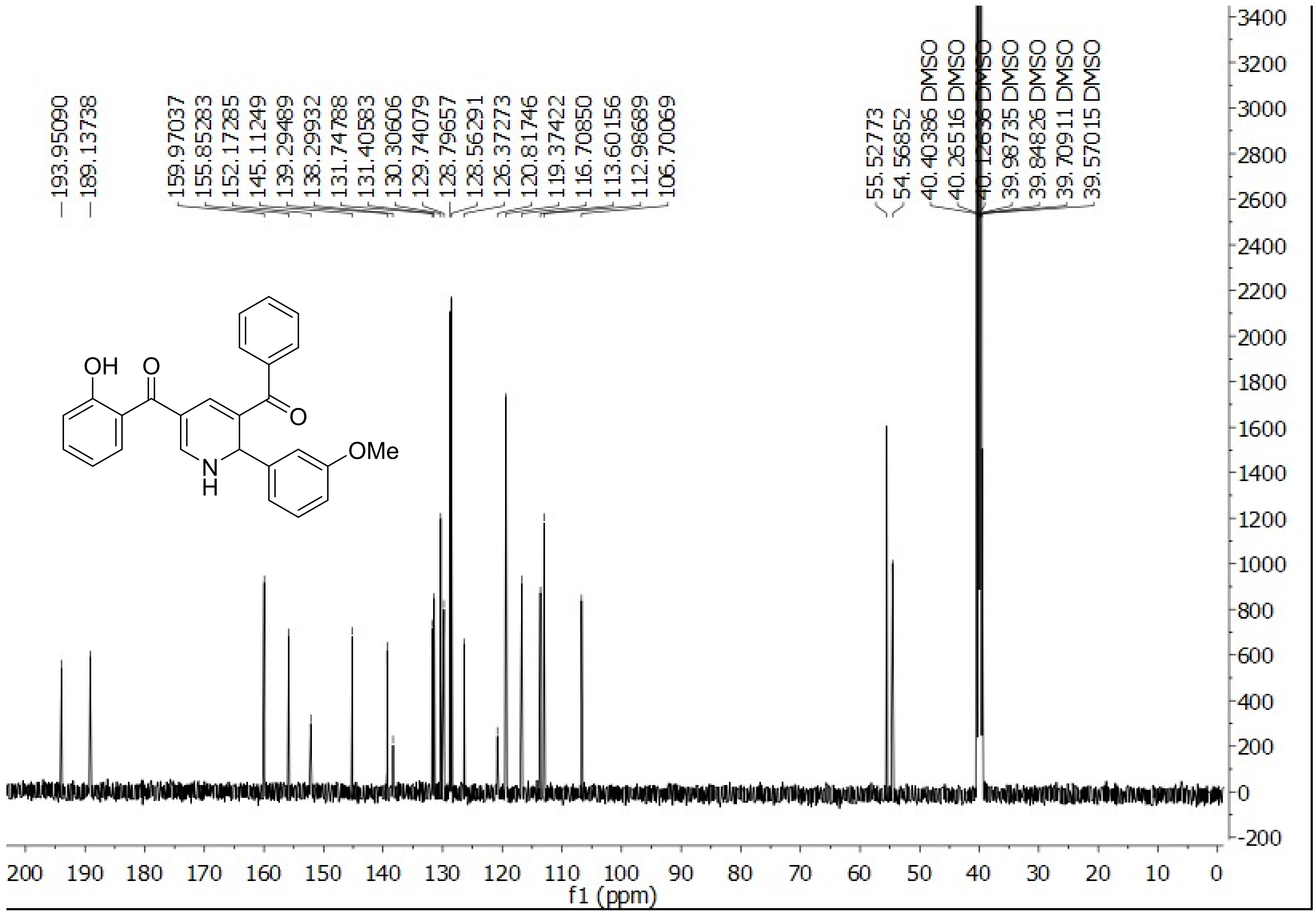

$^{13}C\{^{1}H\}$ NMR spectrum of **3d** (150 MHz, DMSO-d_6)

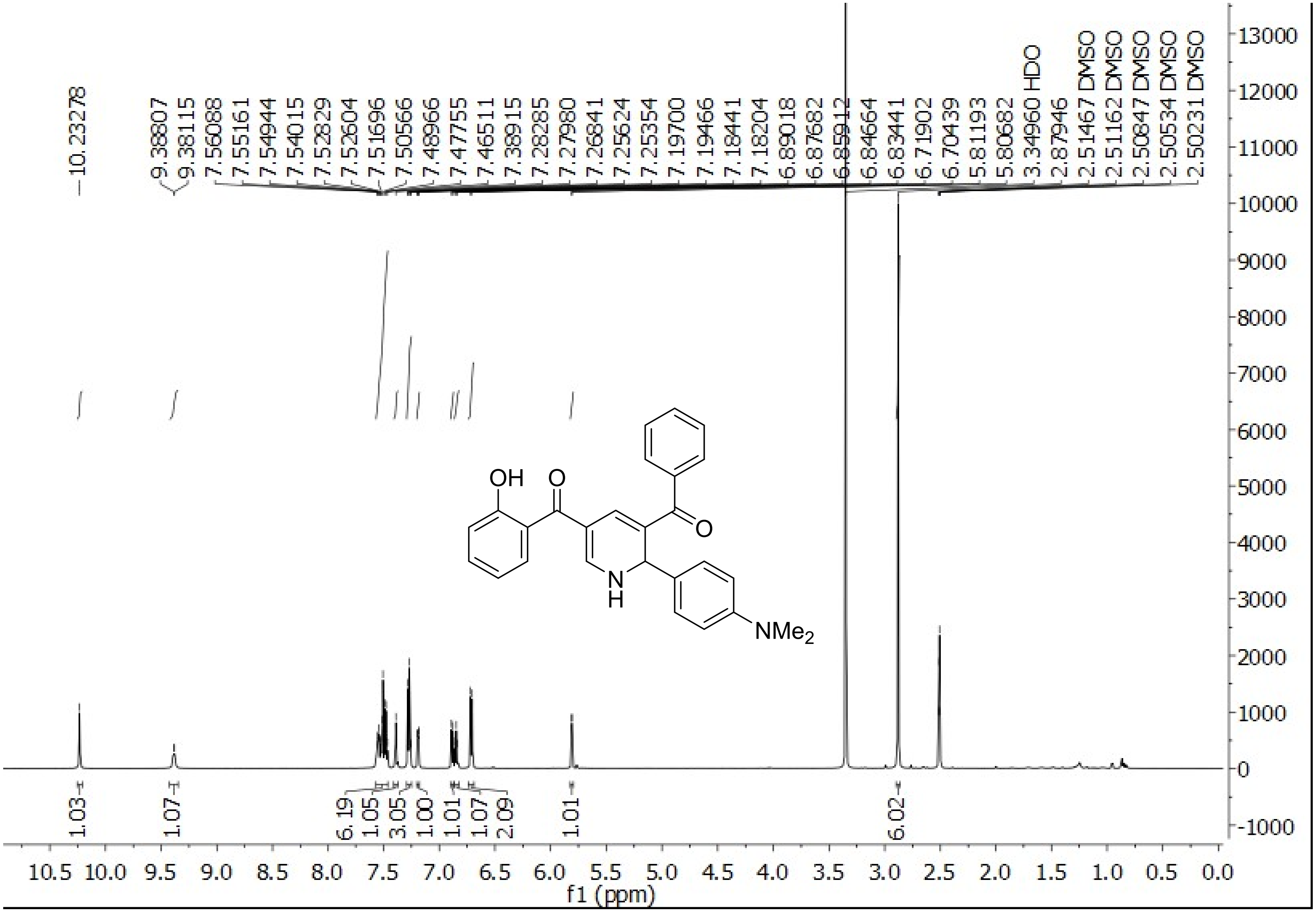

^{1}H NMR spectrum of **3e** (600 MHz, DMSO-d_6)

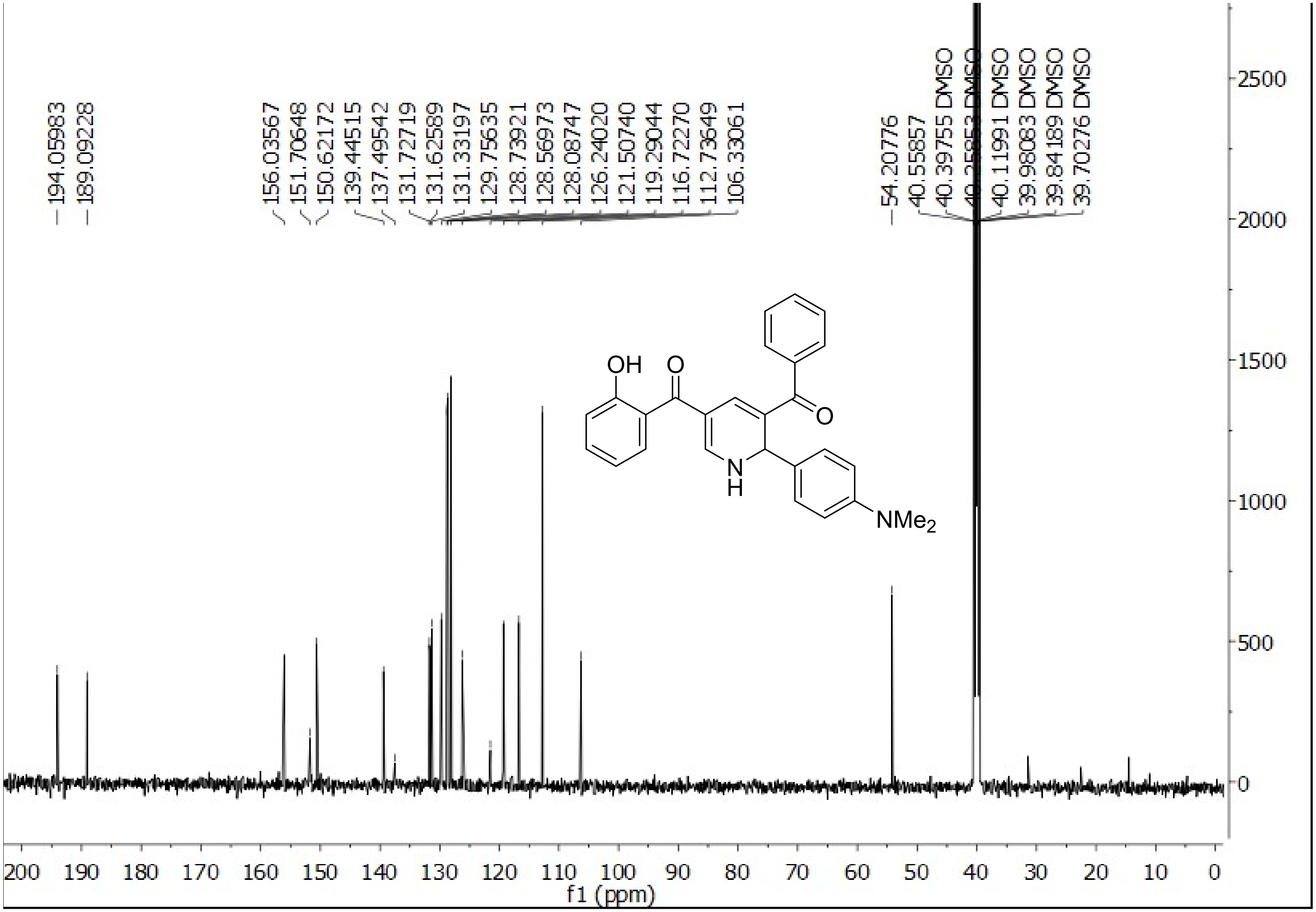

$^{13}C\{^1H\}$ NMR spectrum of **3e** (150 MHz, DMSO-d_6)

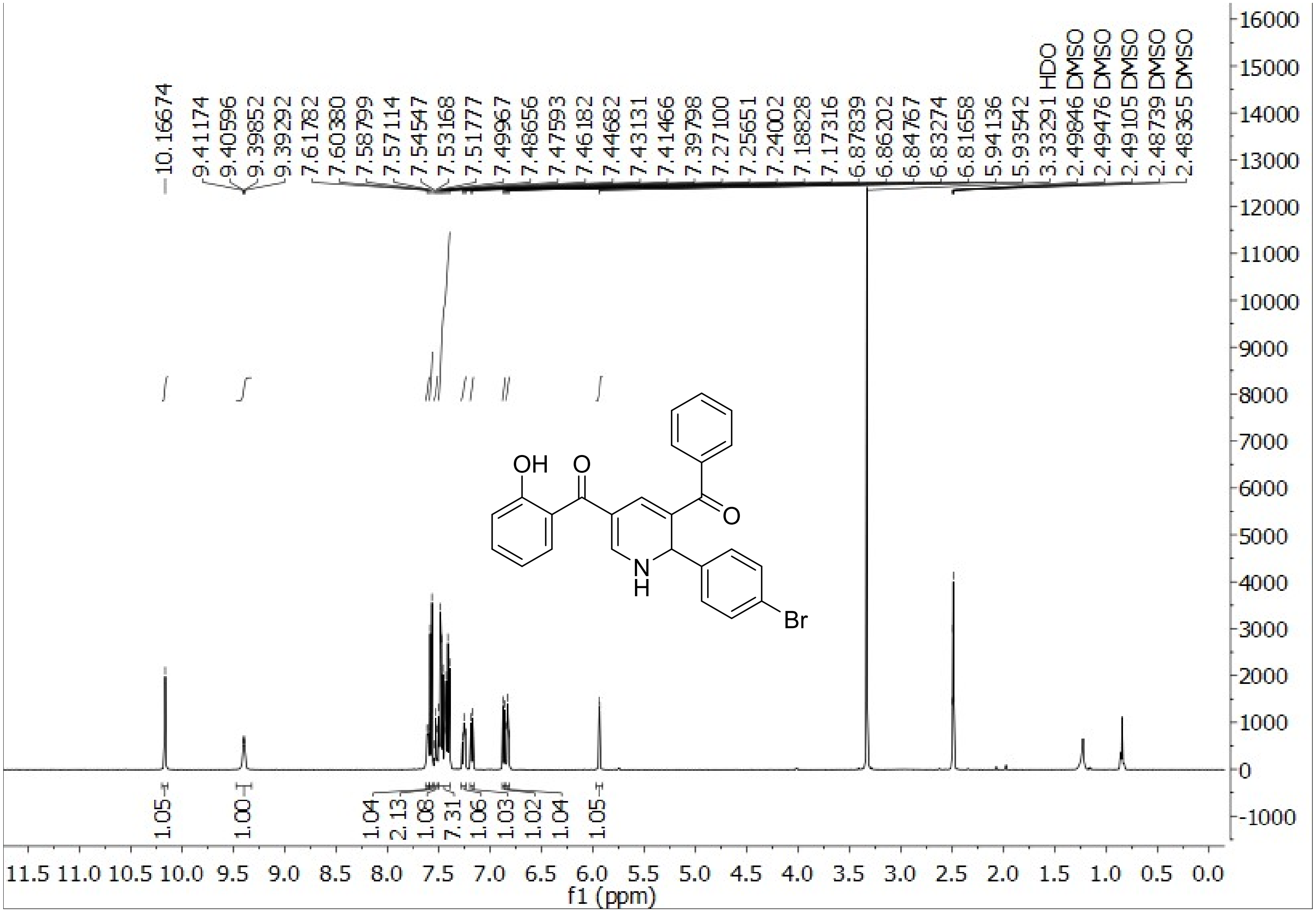

^{1}H NMR spectrum of **3f** (500 MHz, DMSO-d_6)

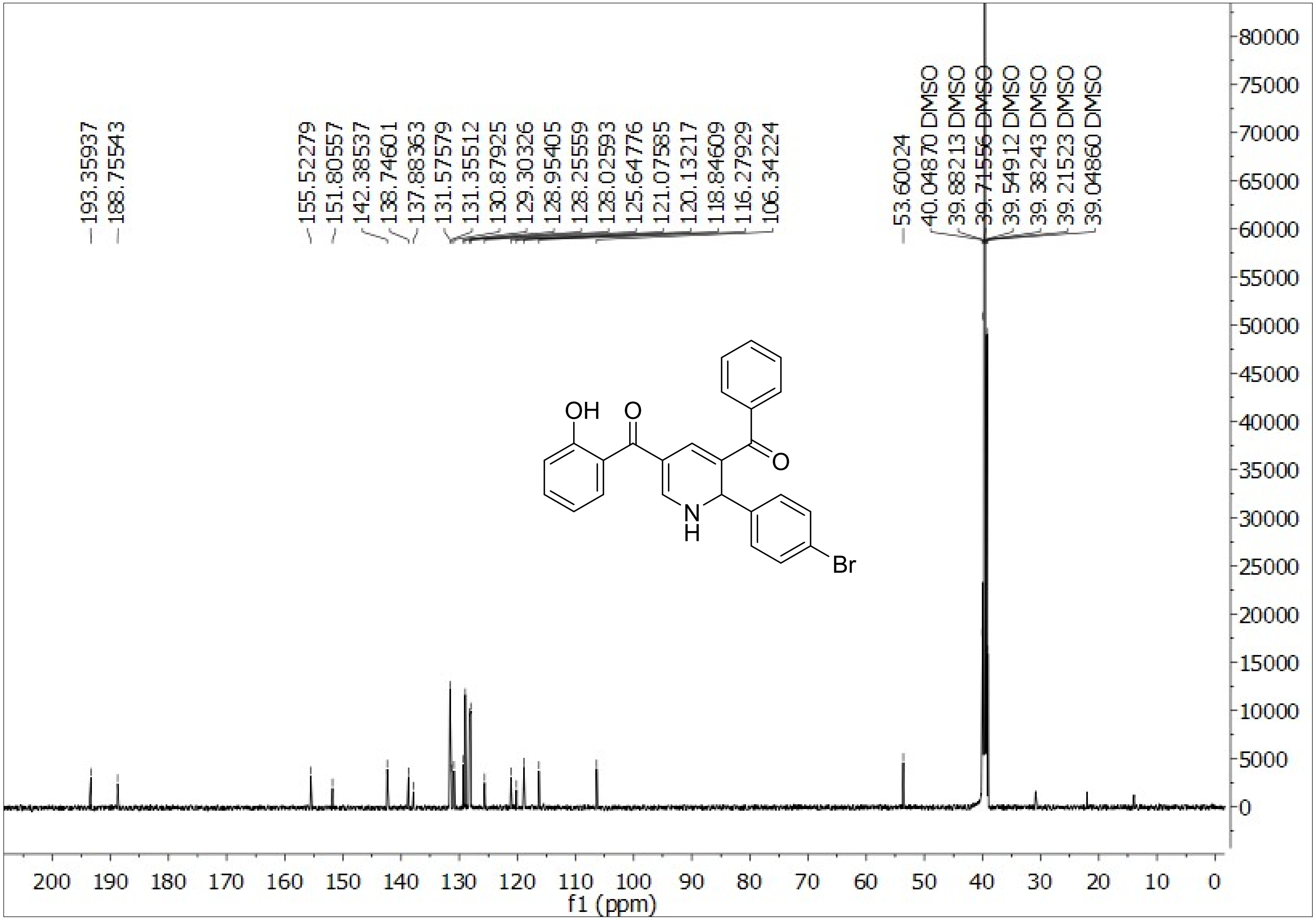

$^{13}C\{^{1}H\}$ NMR spectrum of **3f** (125 MHz, DMSO-d_6)

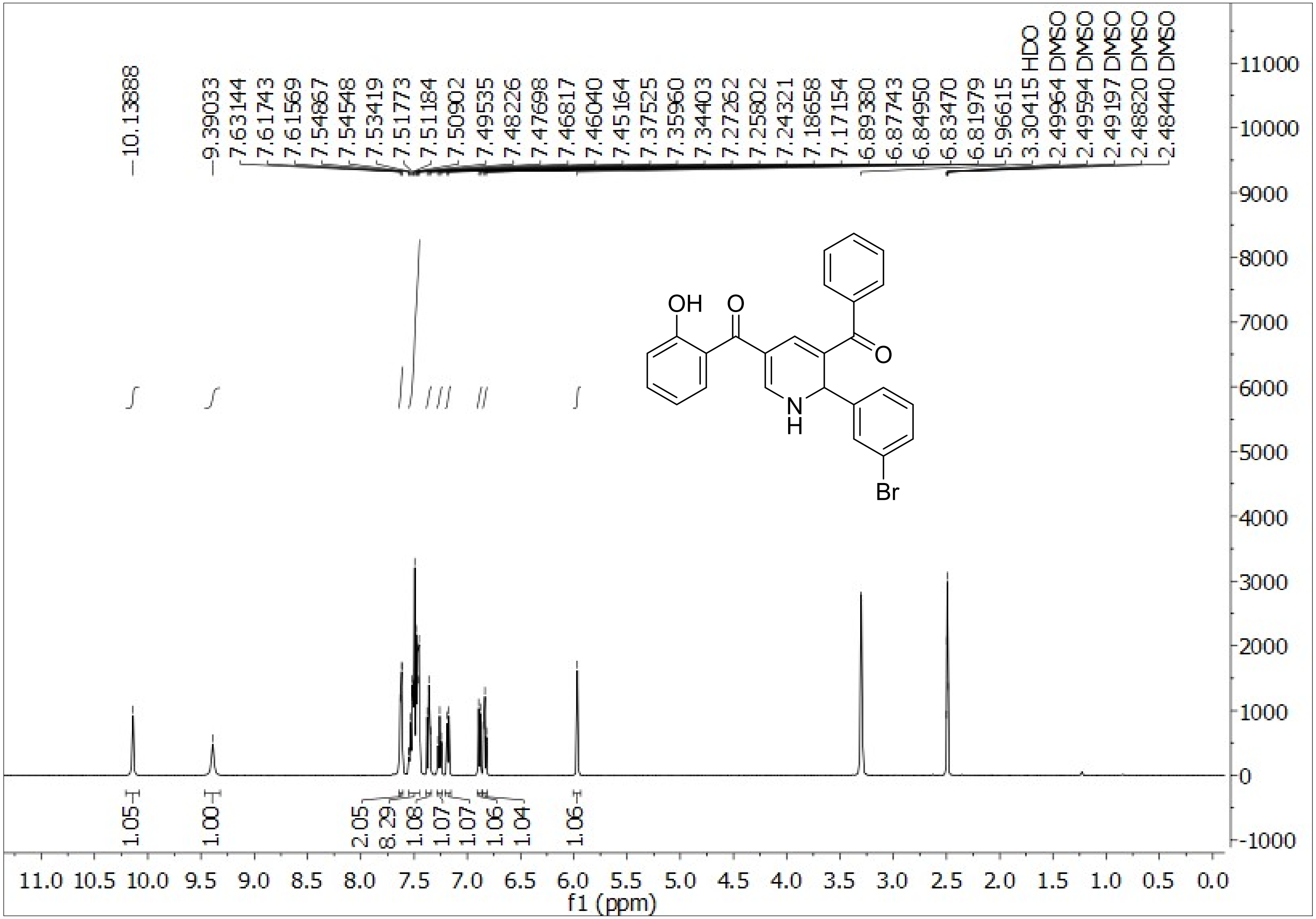

^{1}H NMR spectrum of **3g** (500 MHz, DMSO-d_6)

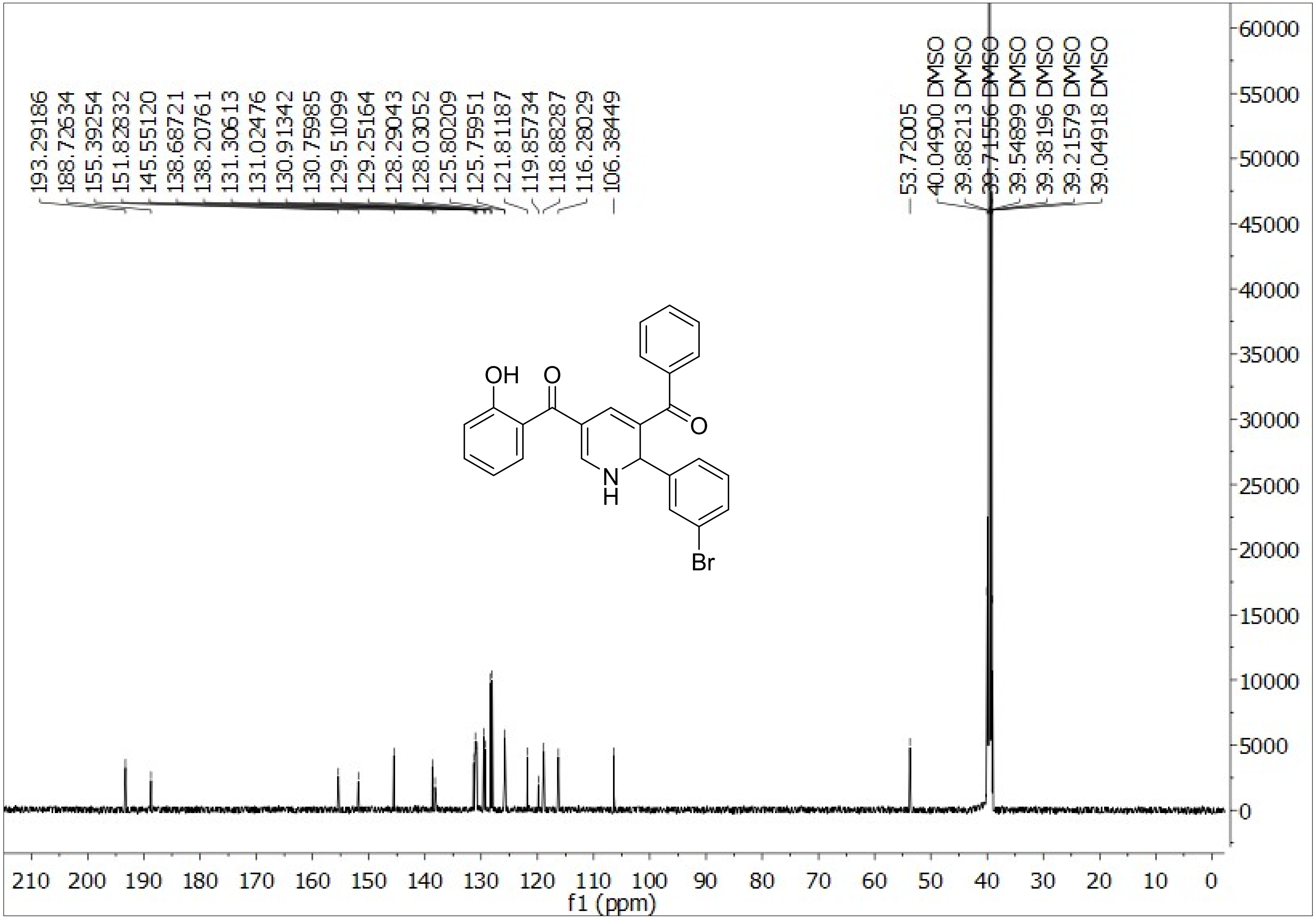

$^{13}C\{^1H\}$ NMR spectrum of **3g** (125 MHz, DMSO-d_6)

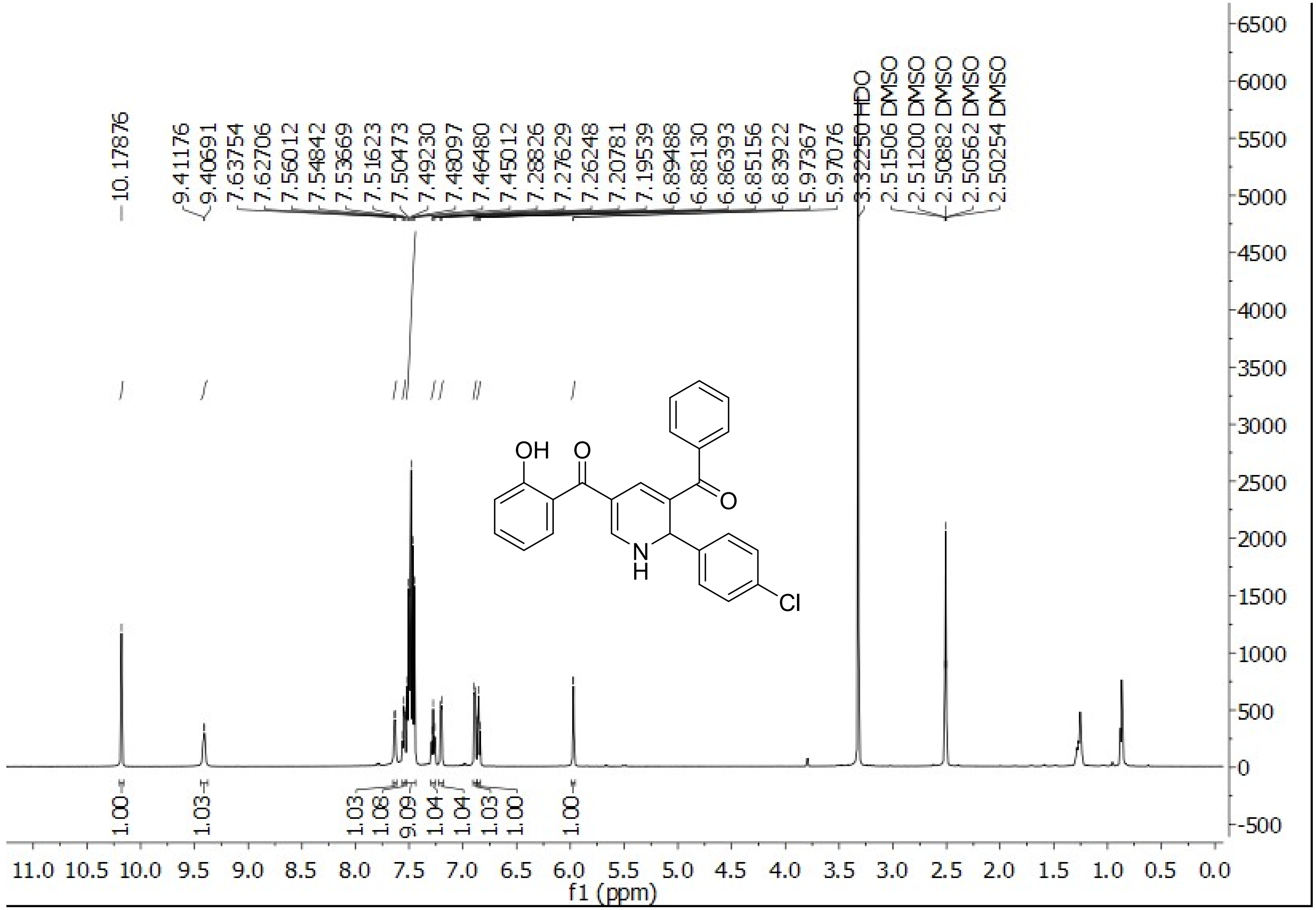

^{1}H NMR spectrum of **3h** (600 MHz, DMSO-d_6)

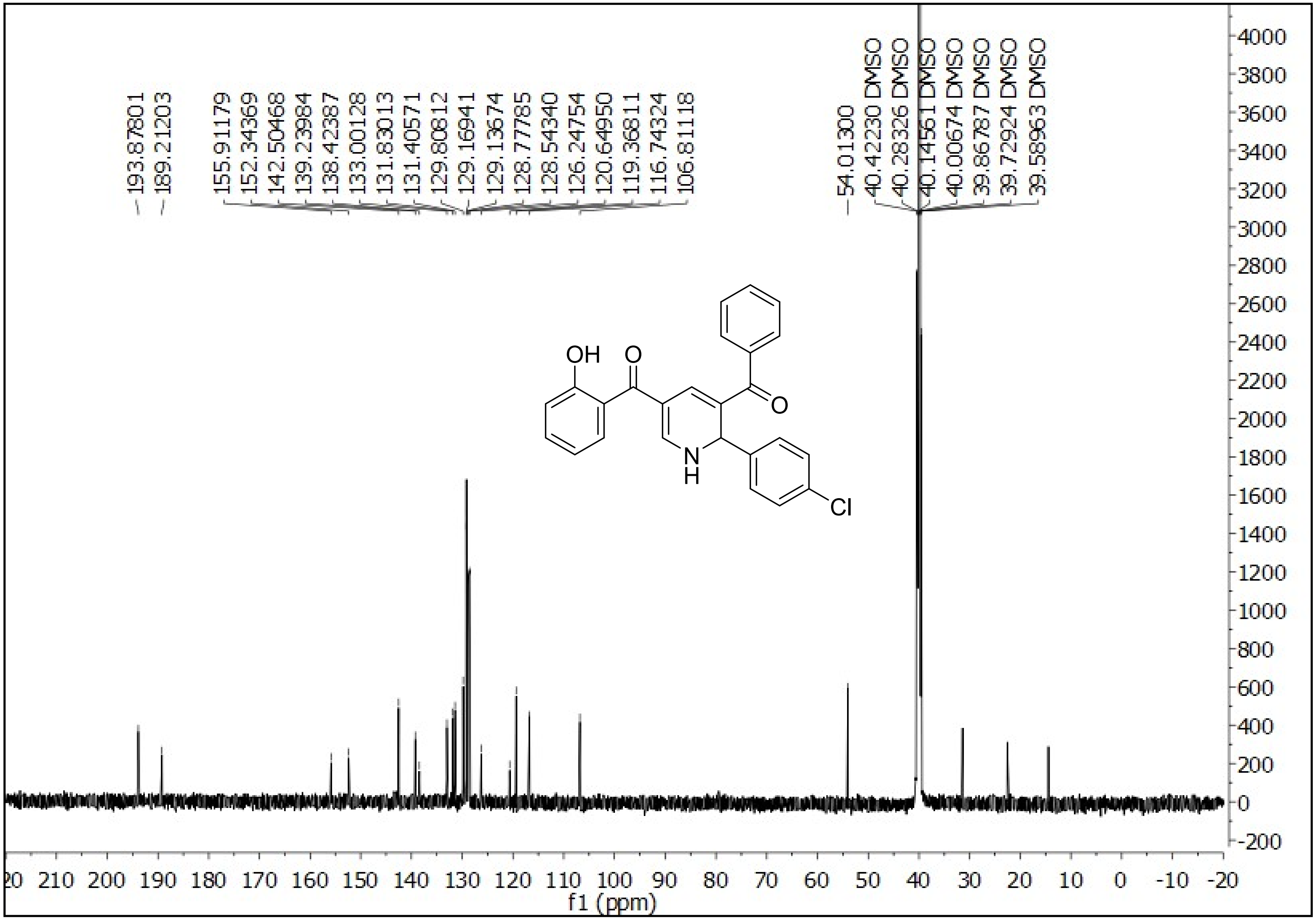

$^{13}C\{^{1}H\}$ NMR spectrum of **3h** (150 MHz, DMSO-d_6)

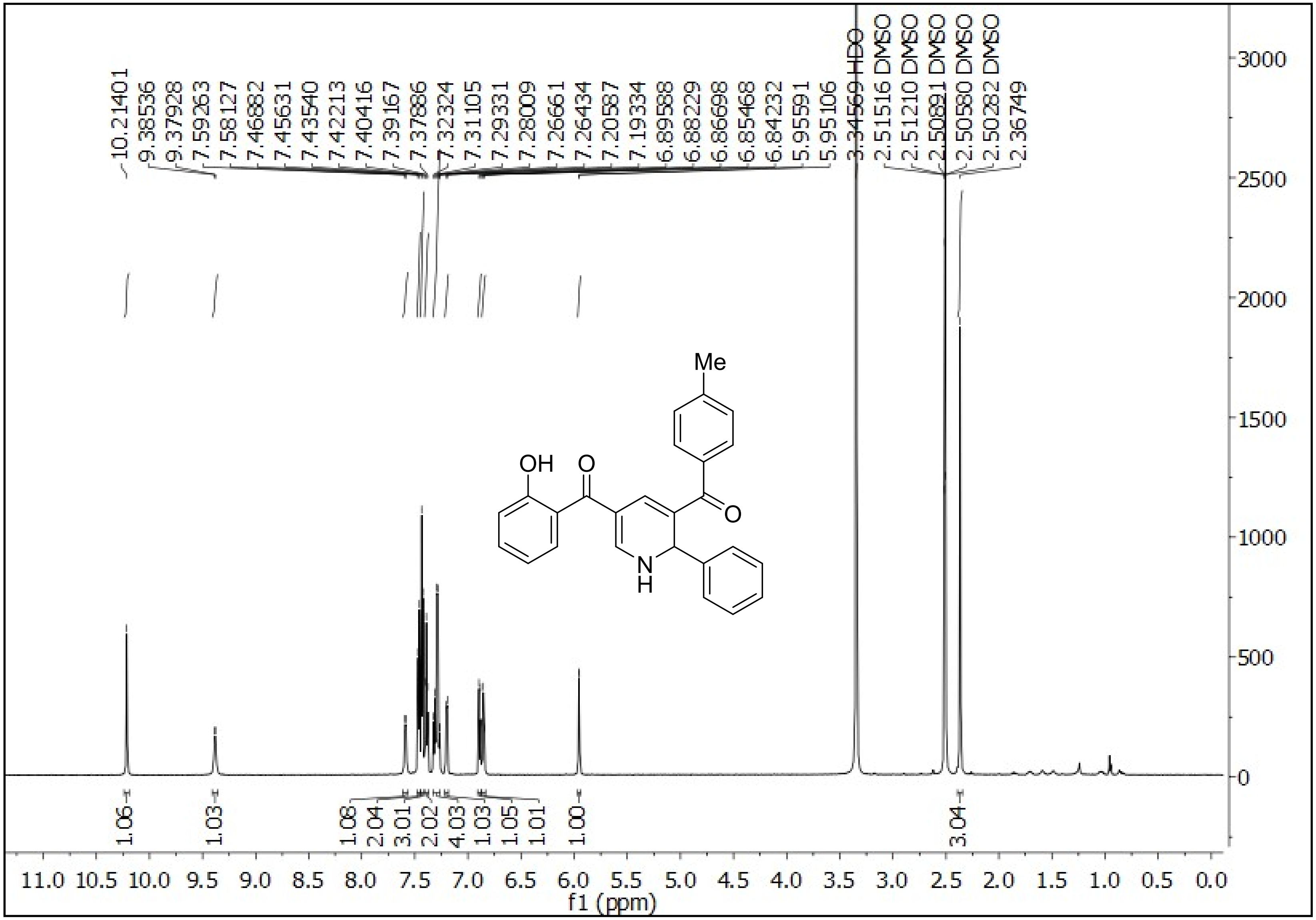

^{1}H NMR spectrum of **3i** (600 MHz, DMSO-d_6)

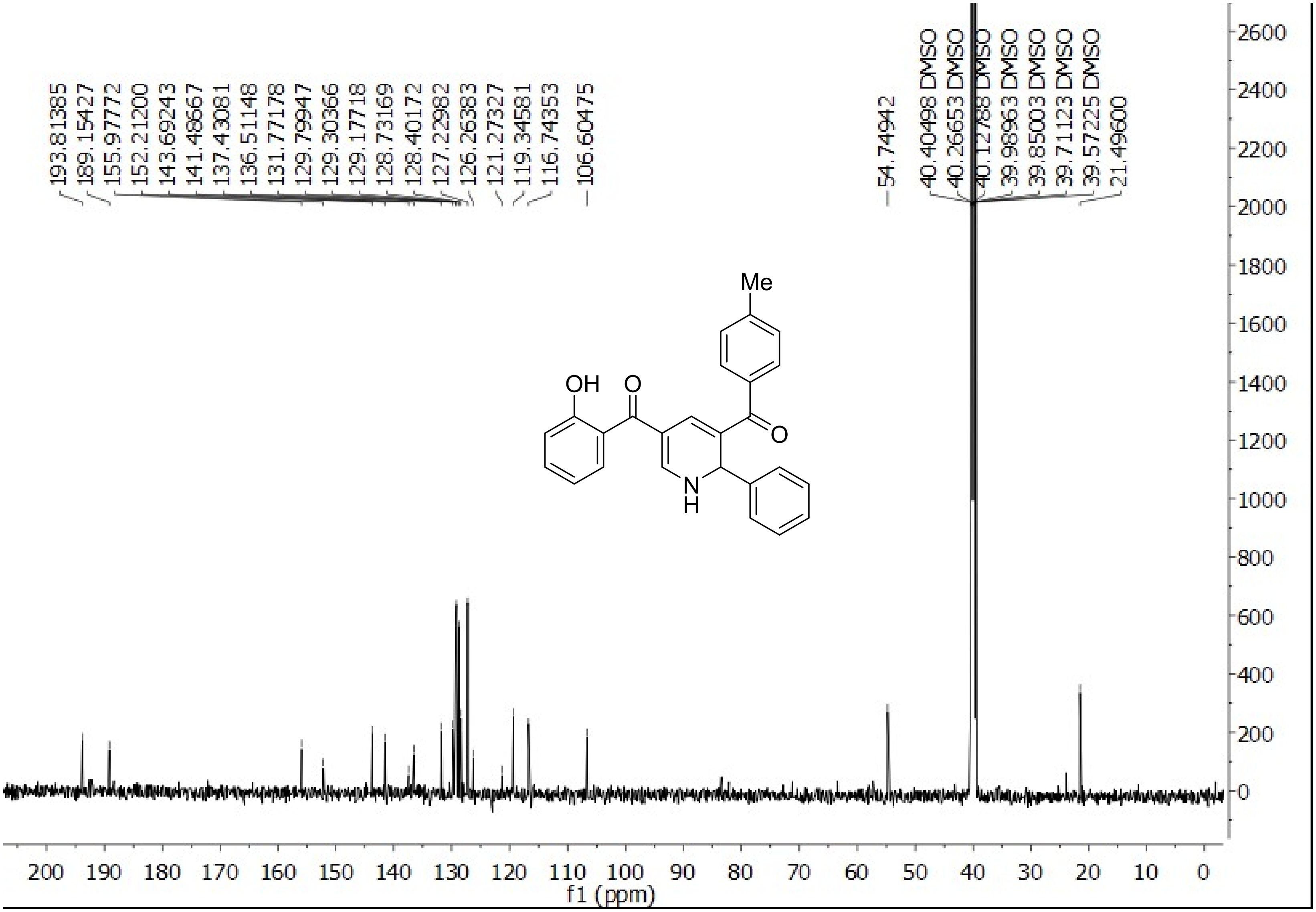

$^{13}C\{^{1}H\}$ NMR spectrum of **3i** (150 MHz, DMSO-d_6)

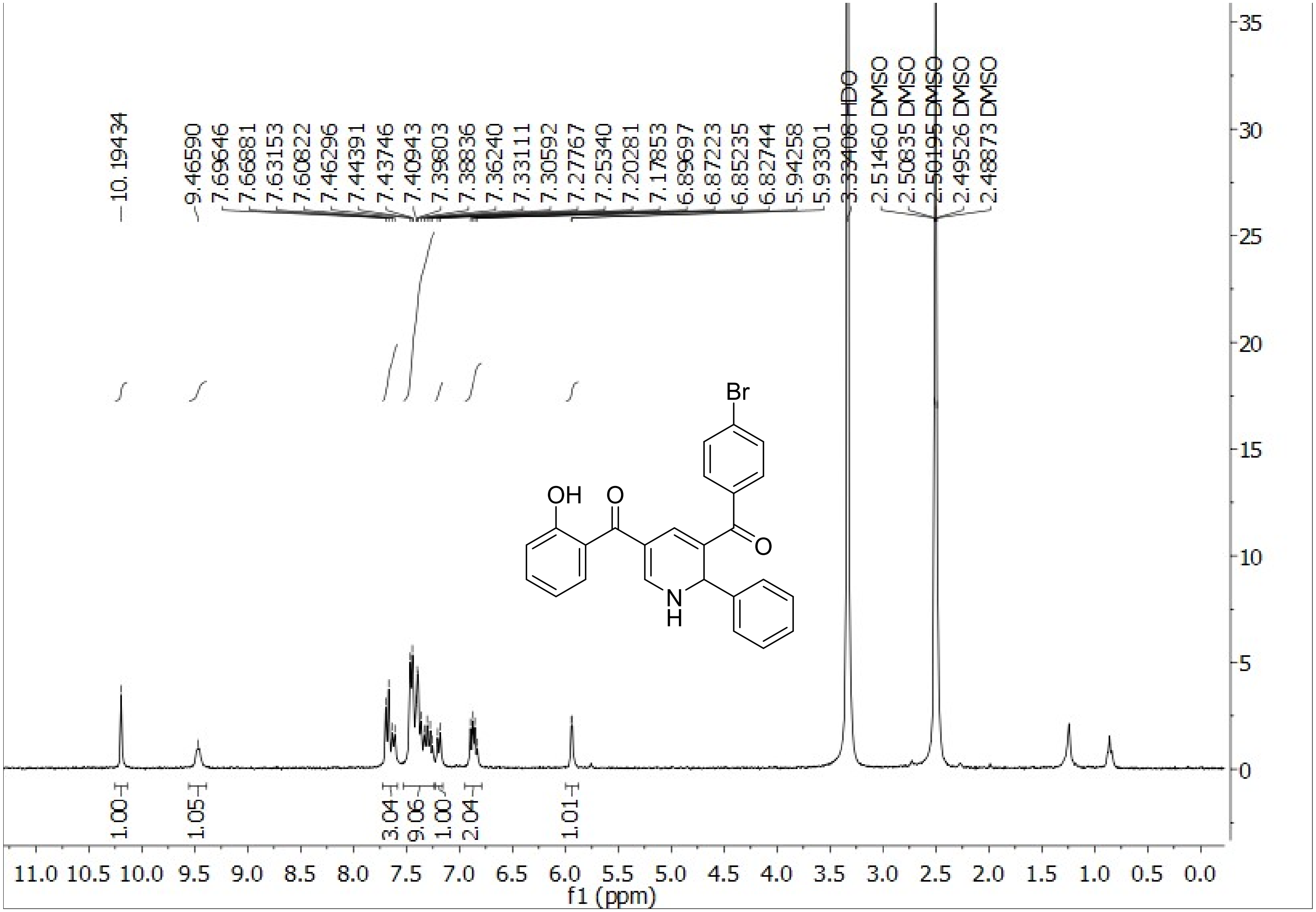

^{1}H NMR spectrum of **3j** (300 MHz, DMSO-d_6)

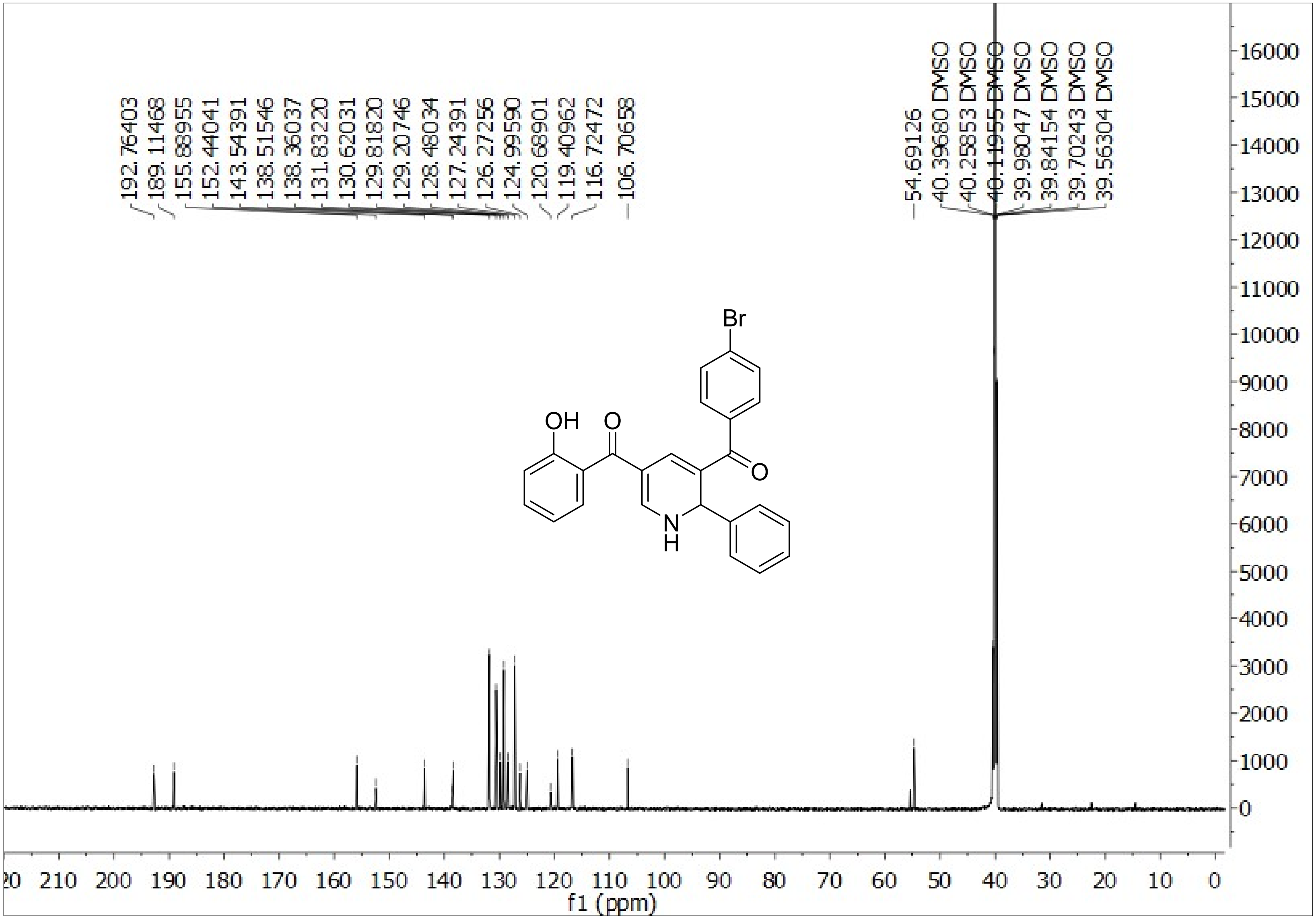

$^{13}C\{^1H\}$ NMR spectrum of **3j** (150 MHz, DMSO-d_6)

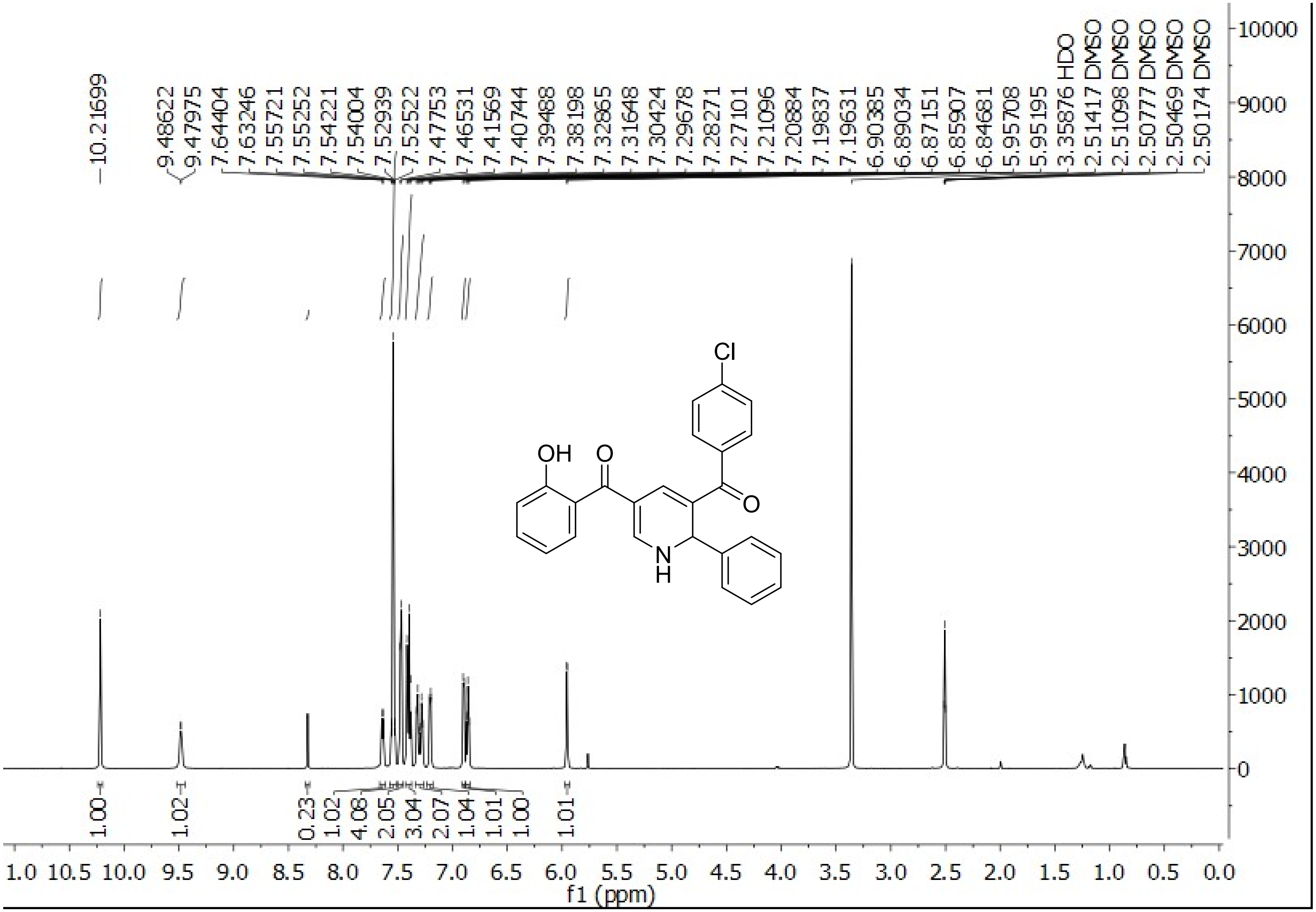

^{1}H NMR spectrum of **3k** (600 MHz, DMSO-d_6)

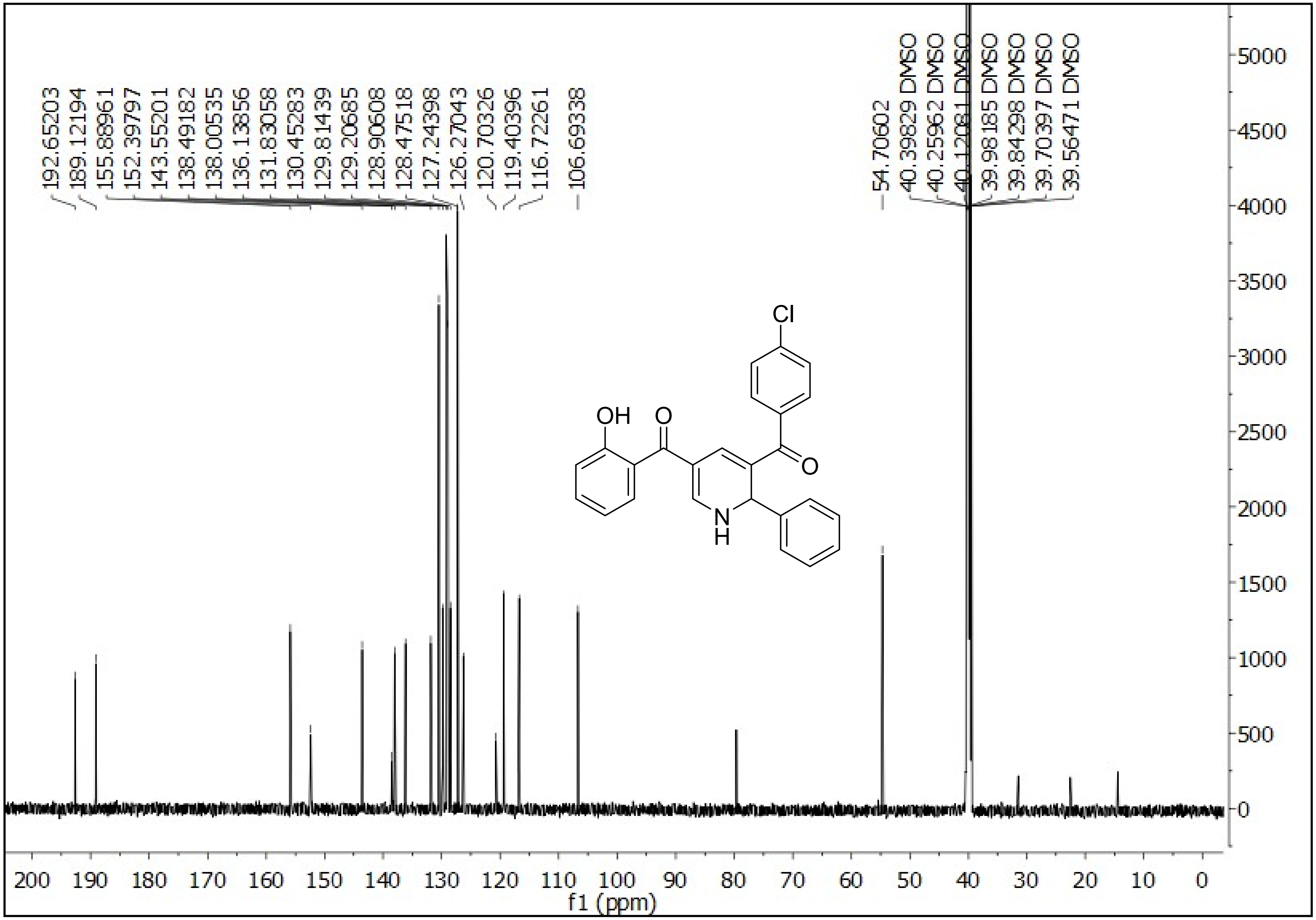

$^{13}C\{^1H\}$ NMR spectrum of **3k** (150 MHz, DMSO-d_6)

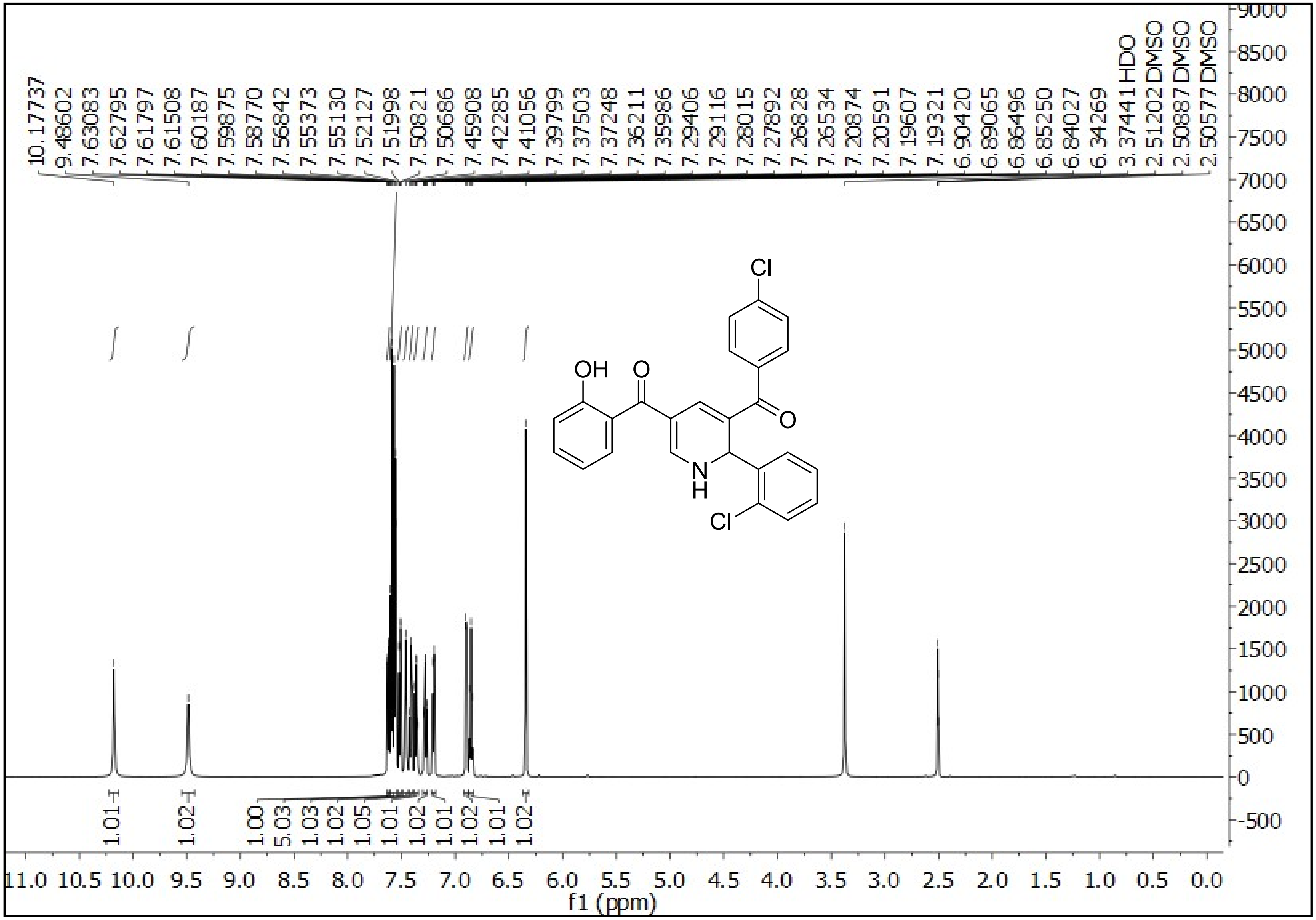

^{1}H NMR spectrum of **3l** (600 MHz, DMSO-d_6)

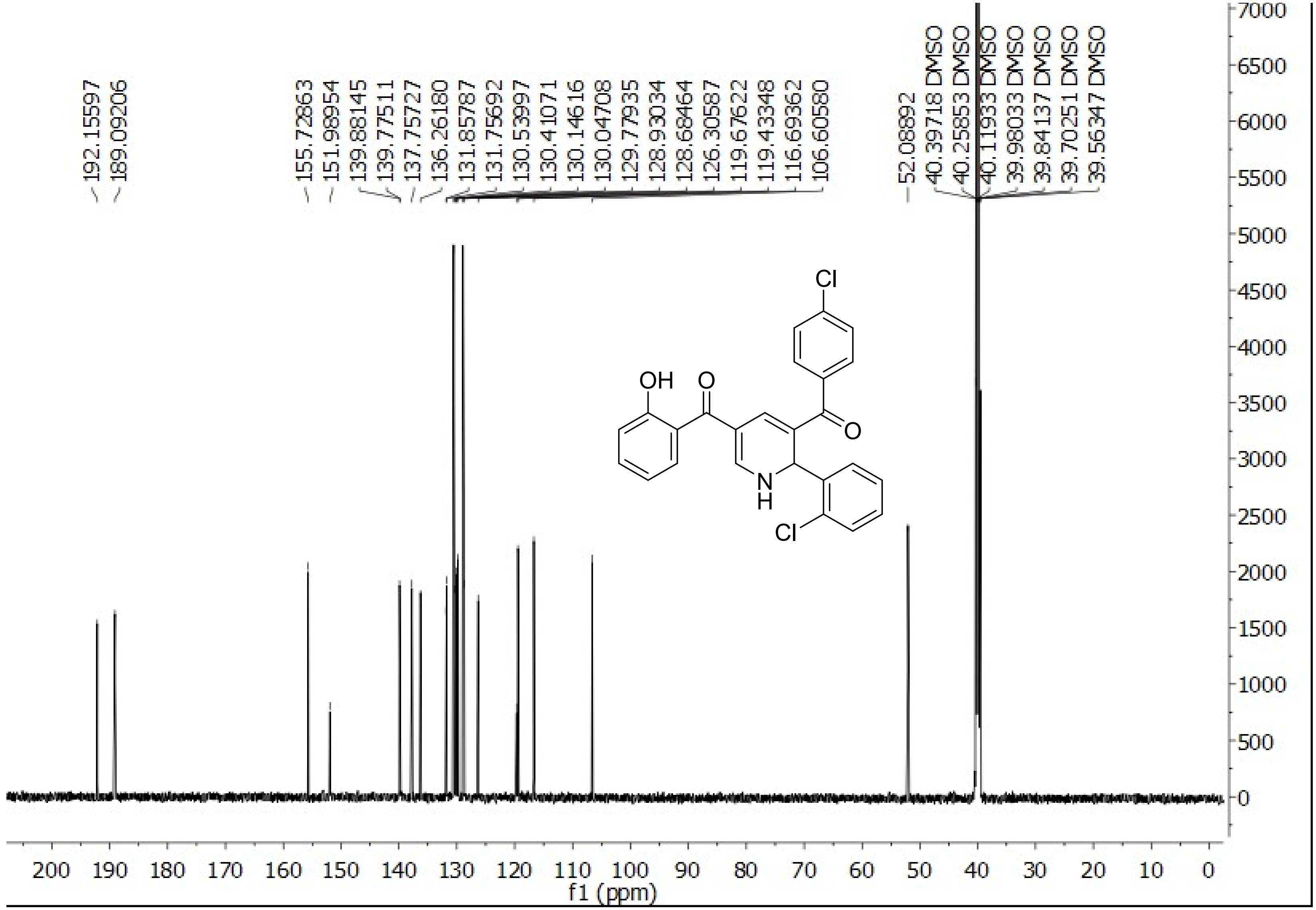

$^{13}C\{^1H\}$ NMR spectrum of **3l** (150 MHz, DMSO-d_6)

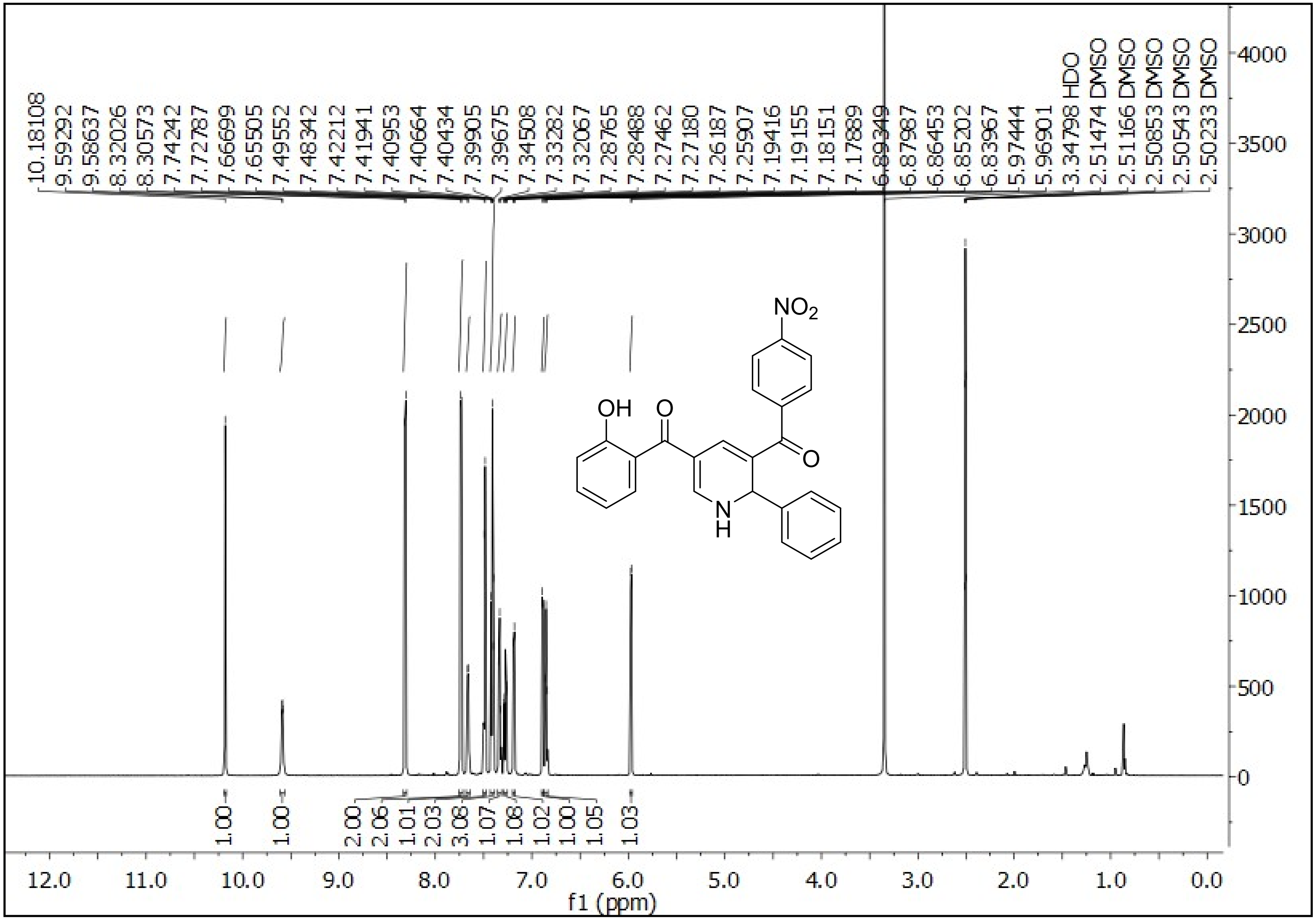

^{1}H NMR spectrum of **3m** (600 MHz, DMSO-d_6)

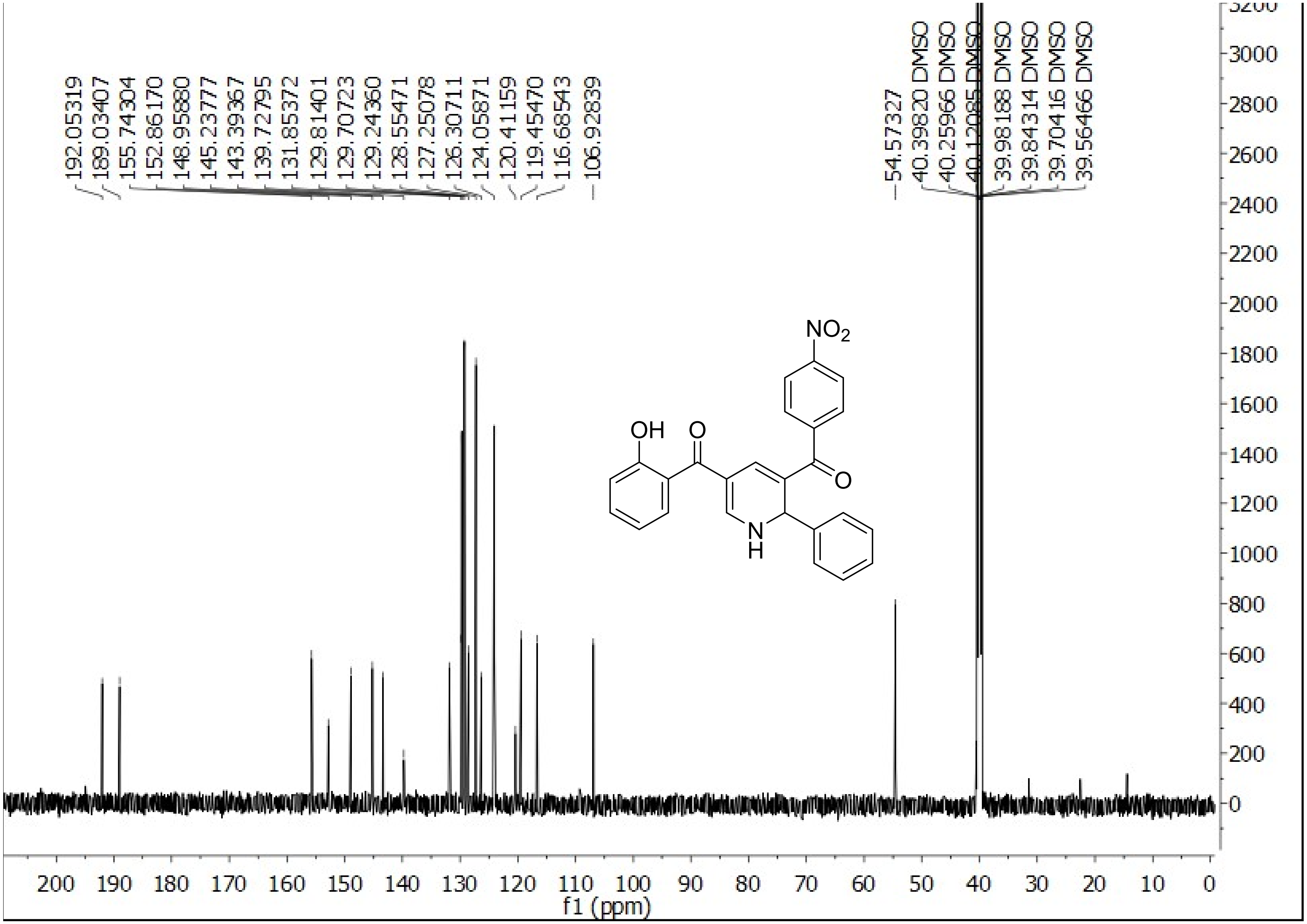

$^{13}C\{^1H\}$ NMR spectrum of **3m** (150 MHz, DMSO-d_6)

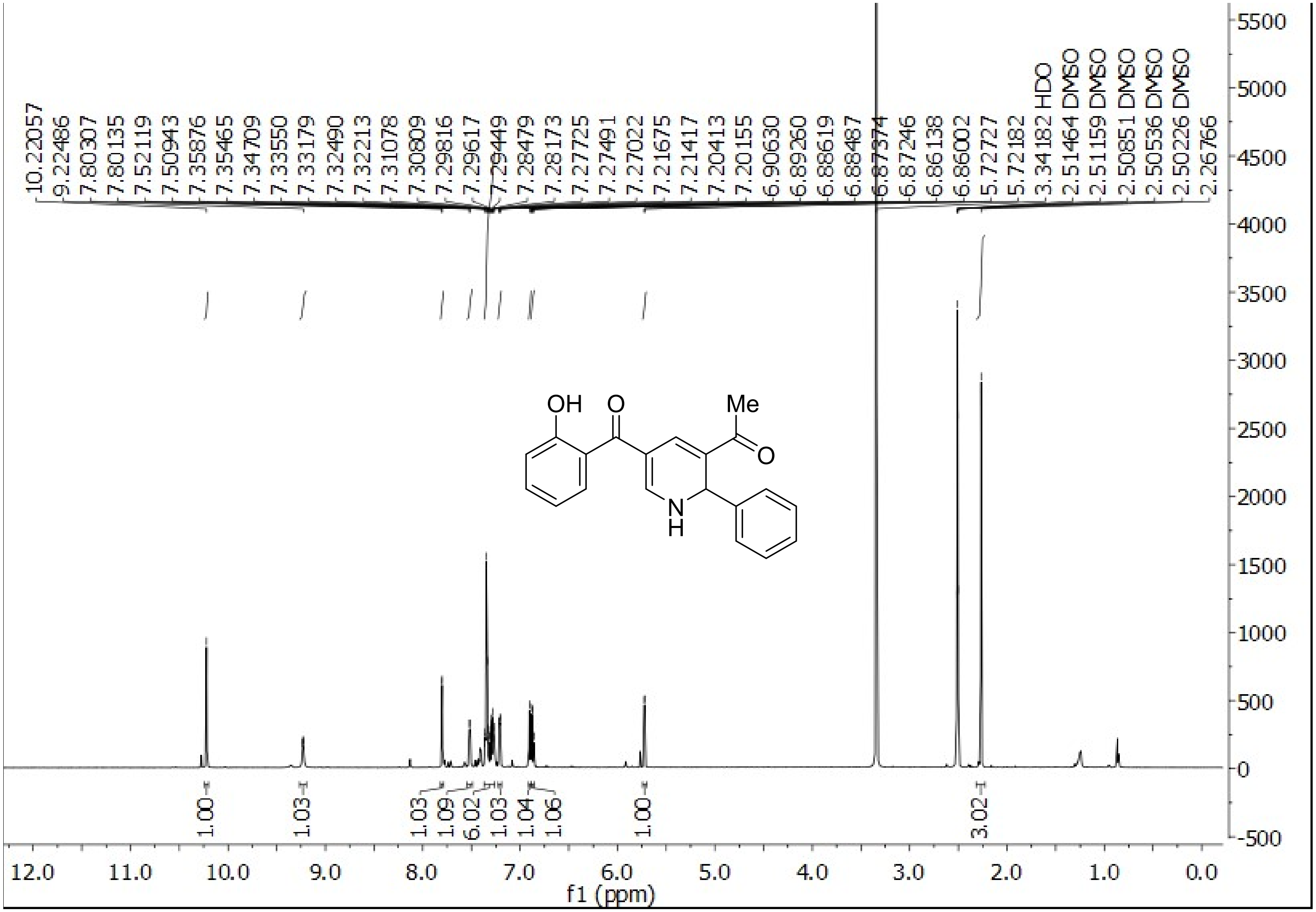

^{1}H NMR spectrum of **3n** (600 MHz, DMSO-d_6)

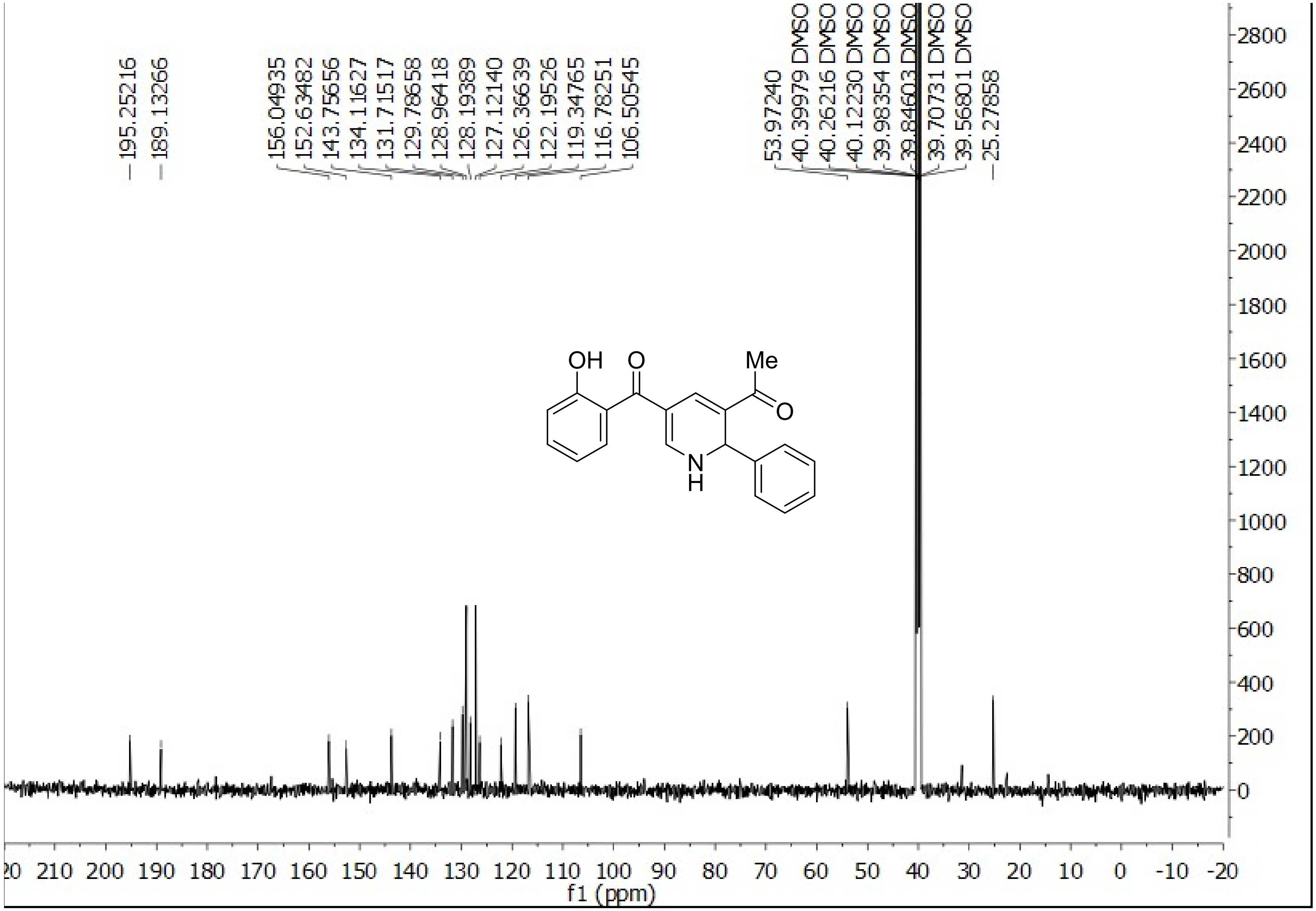

$^{13}C\{^1H\}$ NMR spectrum of **3n** (150 MHz, DMSO-d_6)

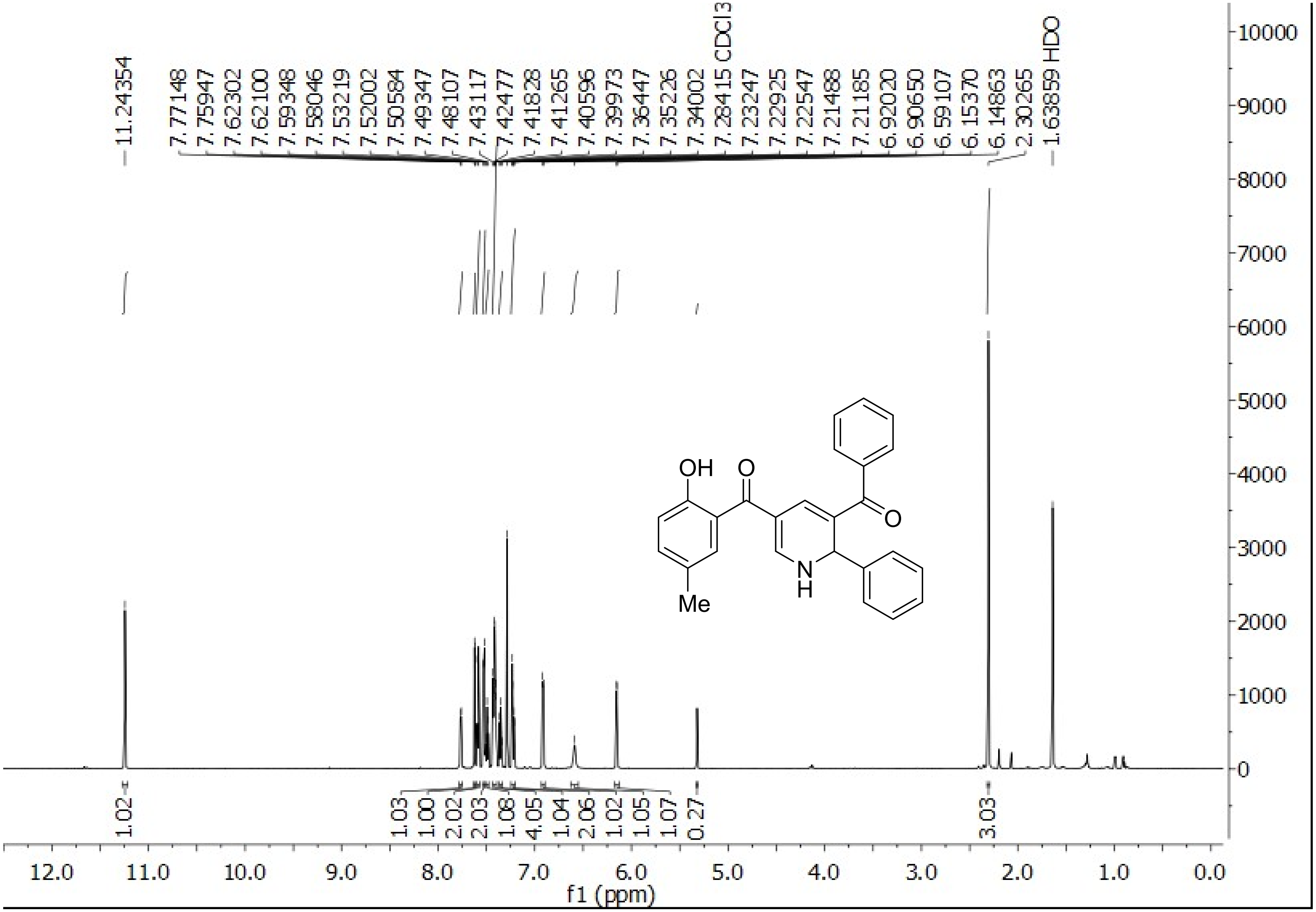

^{1}H NMR spectrum of **3o** (600 MHz, $CDCl_3$)

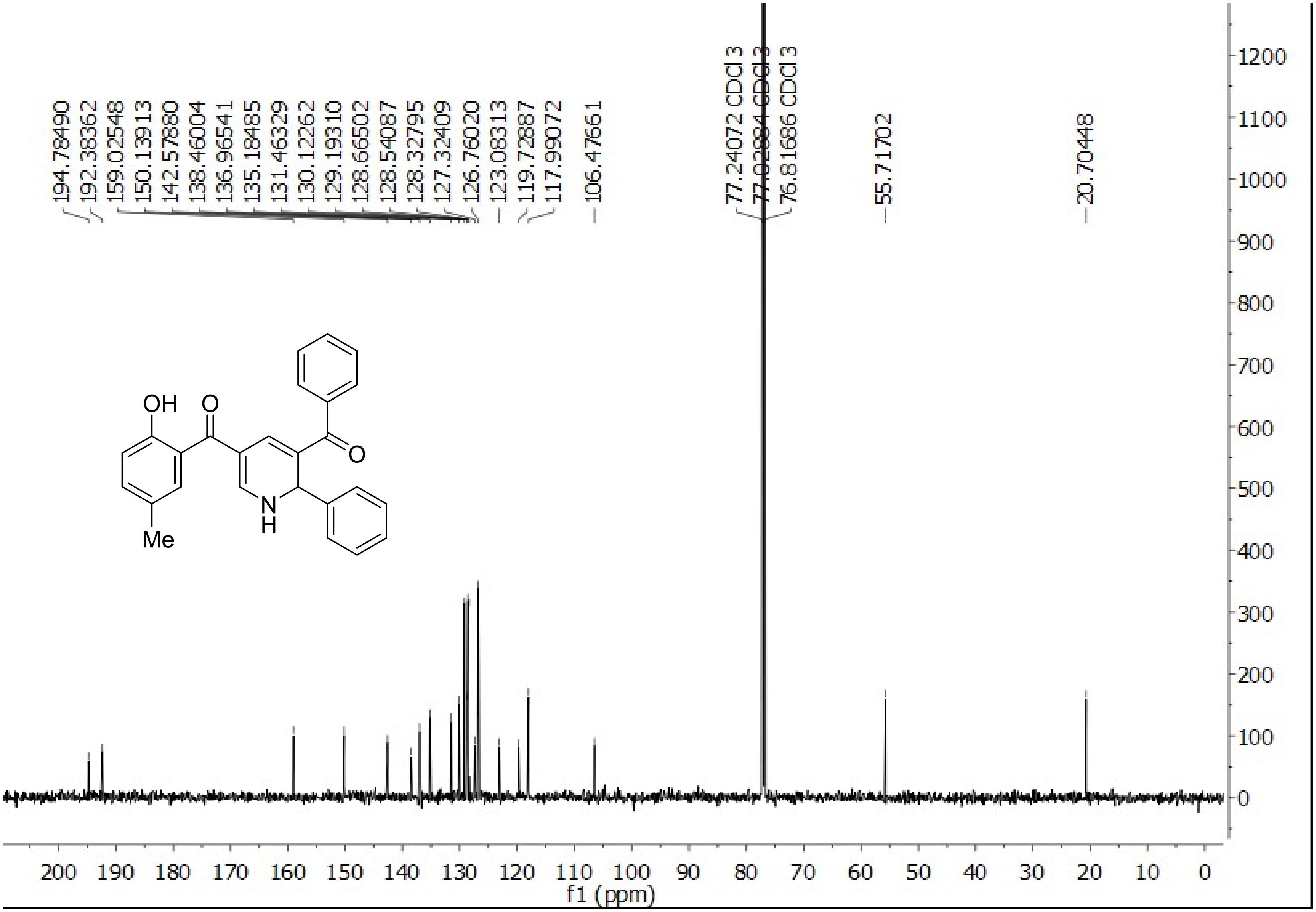

$^{13}C\{^1H\}$ NMR spectrum of **3o** (150 MHz, $CDCl_3$)

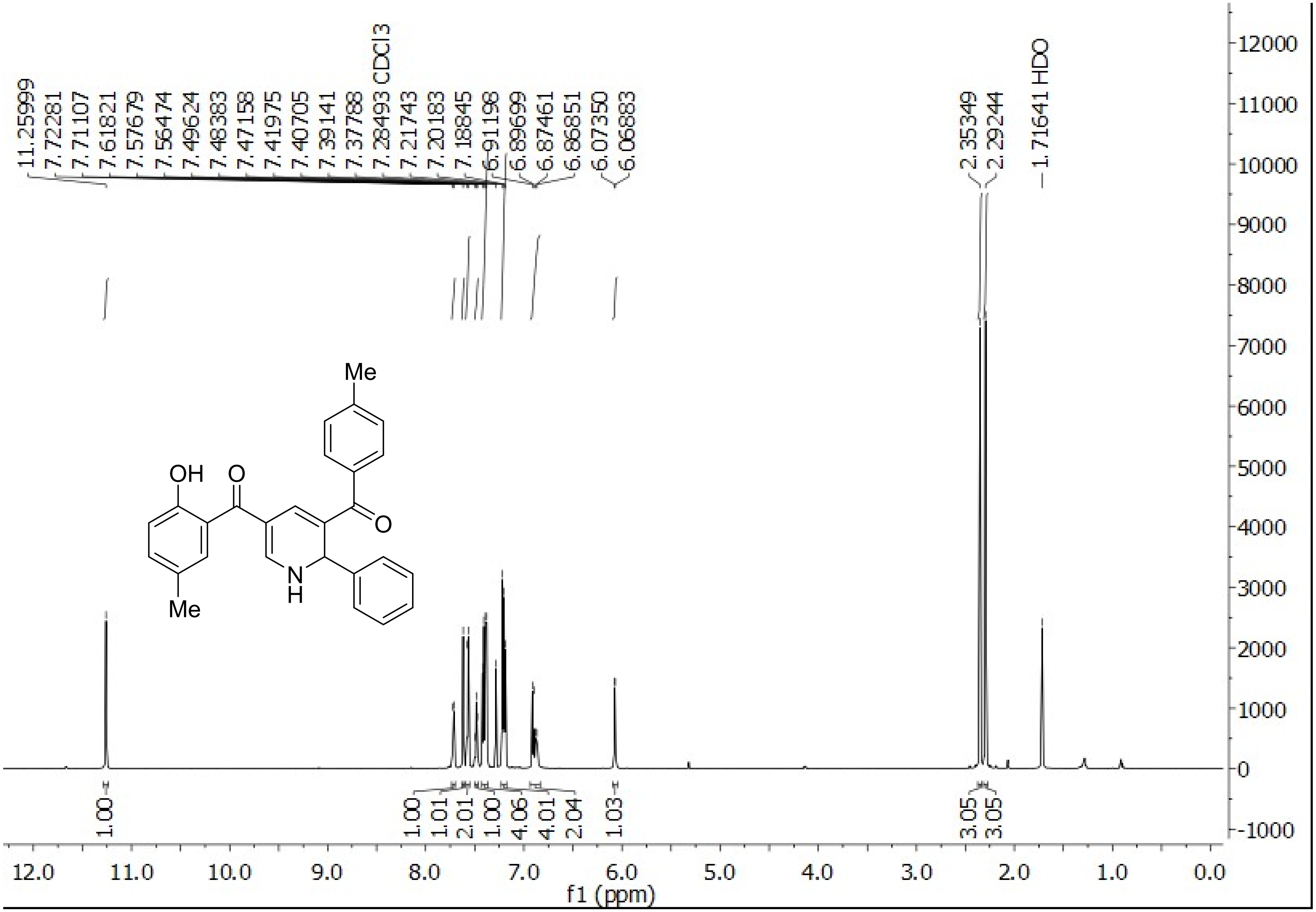

^{1}H NMR spectrum of **3p** (600 MHz, $CDCl_3$)

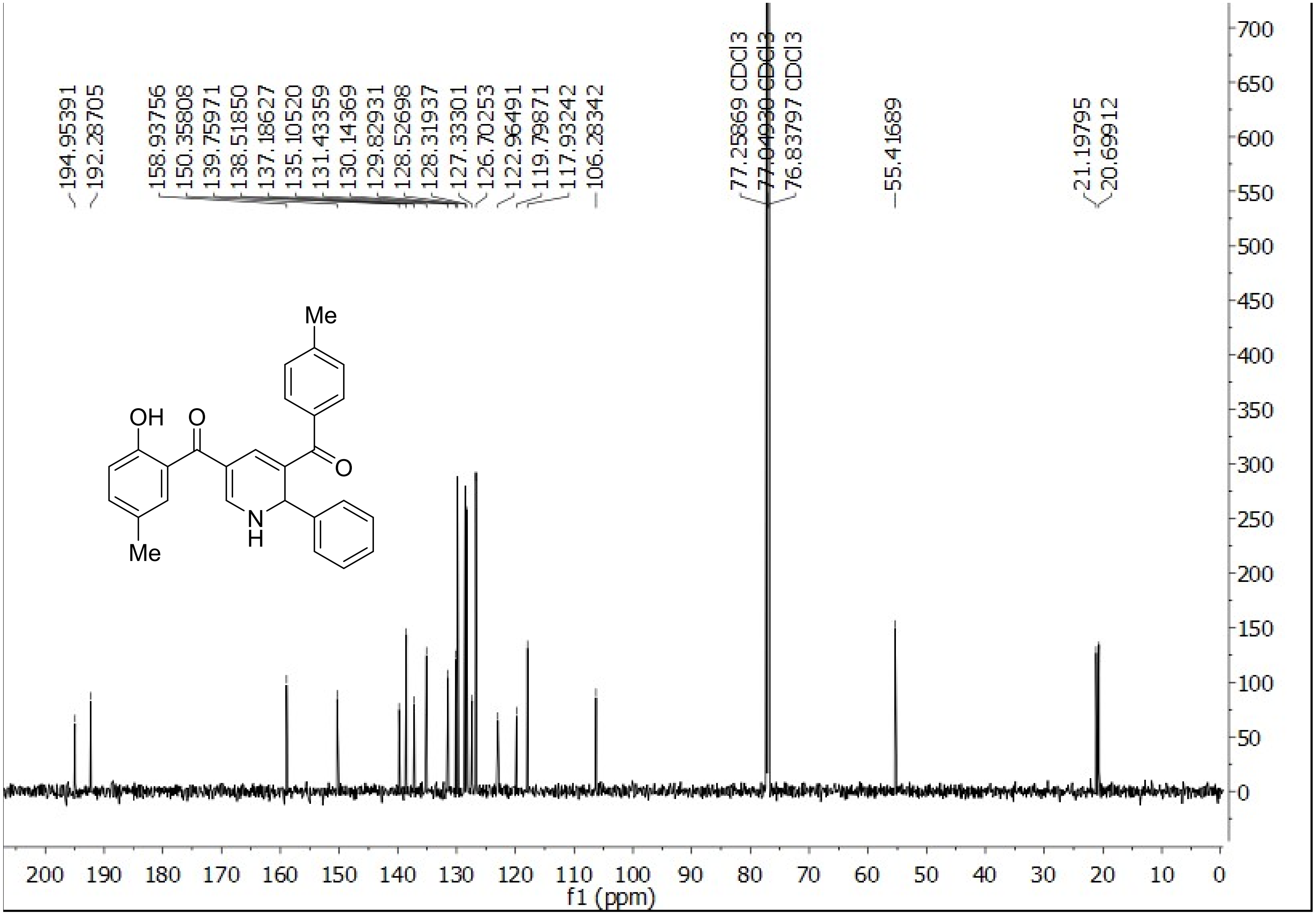

$^{13}C\{^{1}H\}$ NMR spectrum of **3p** (150 MHz, $CDCl_3$)

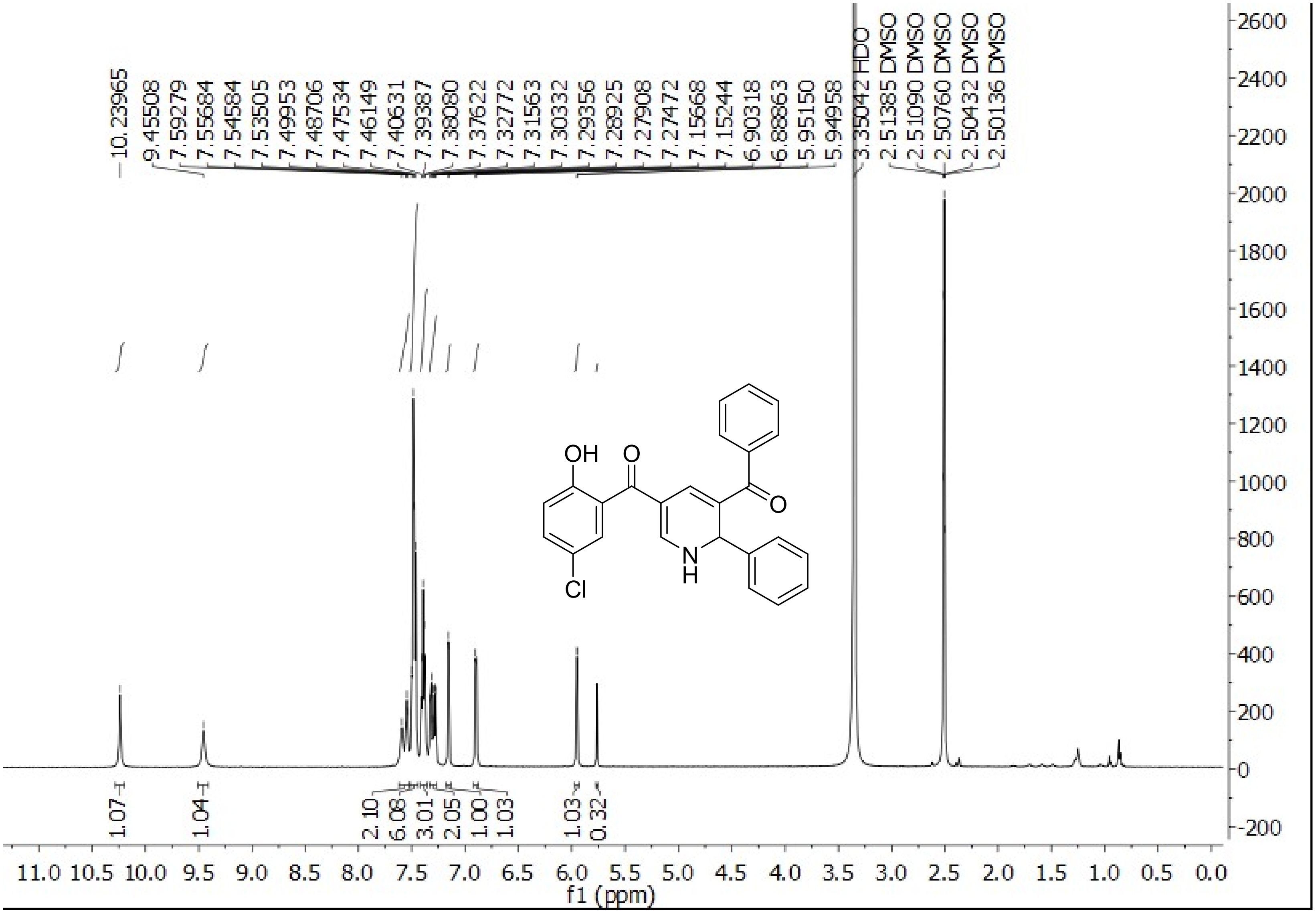

^{1}H NMR spectrum of **3q** (600 MHz, DMSO-d_6)

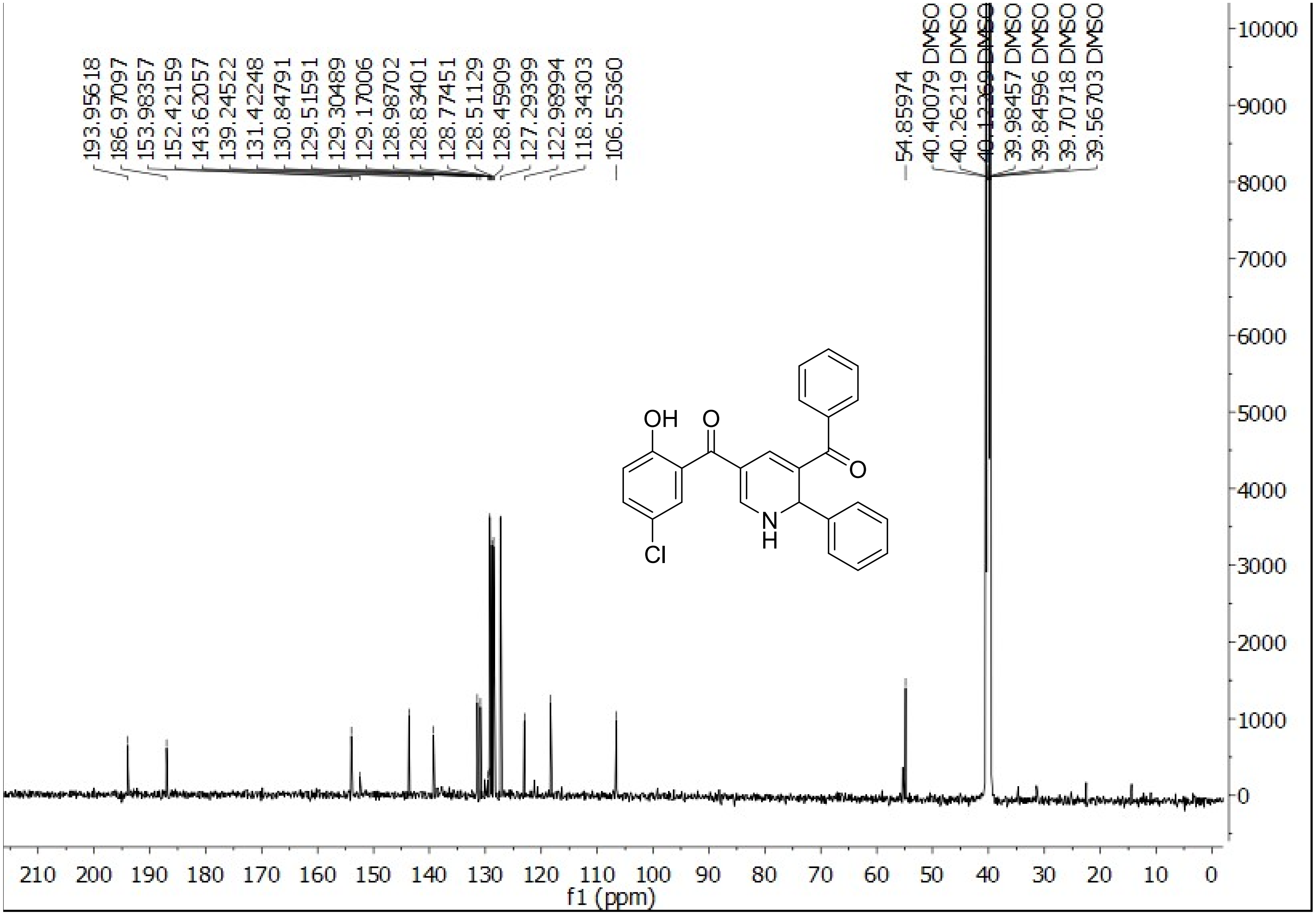

$^{13}C\{^1H\}$ NMR spectrum of **3q** (150 MHz, DMSO-d_6)

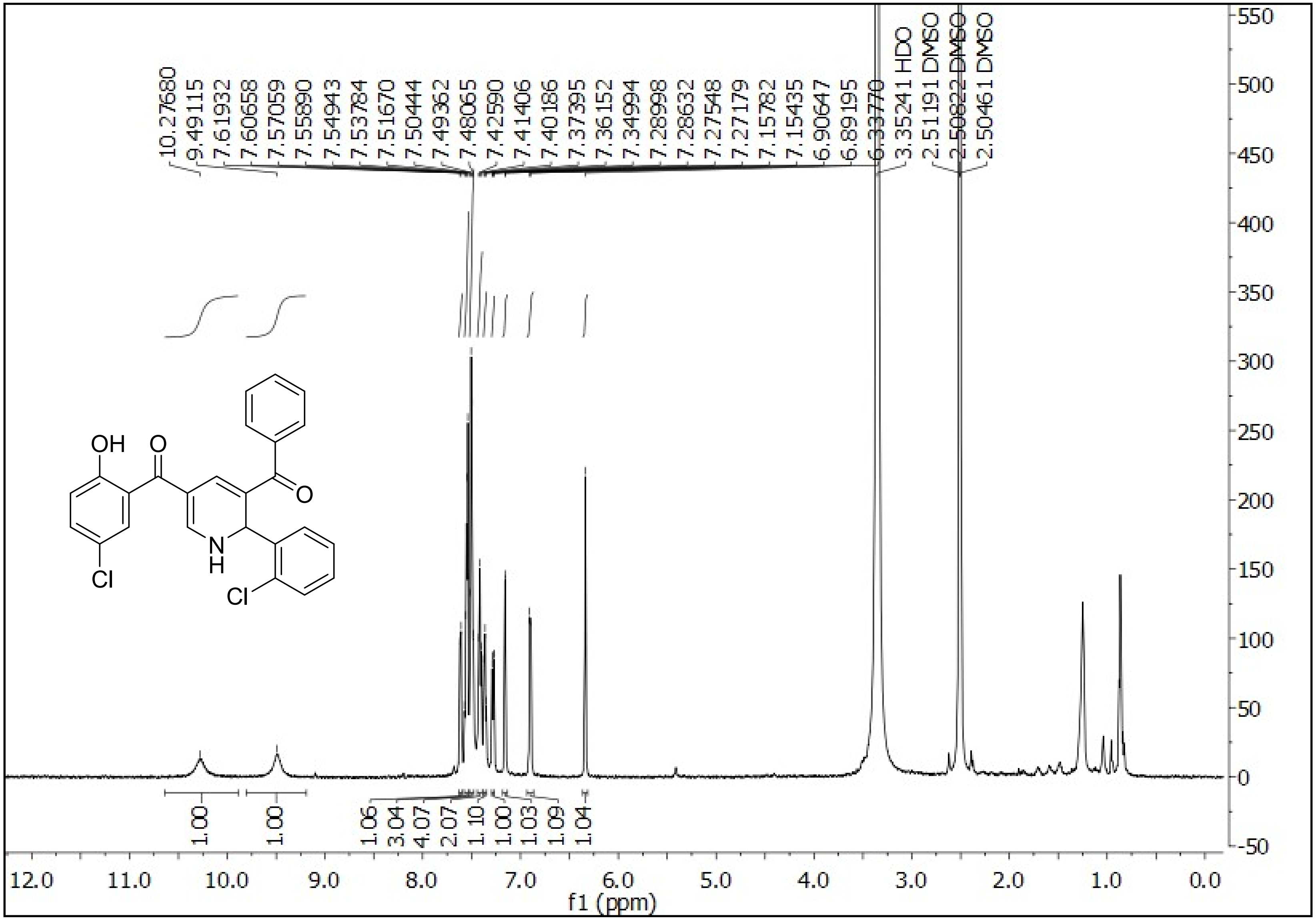

^{1}H NMR spectrum of **3r** (600 MHz, DMSO-d_6)

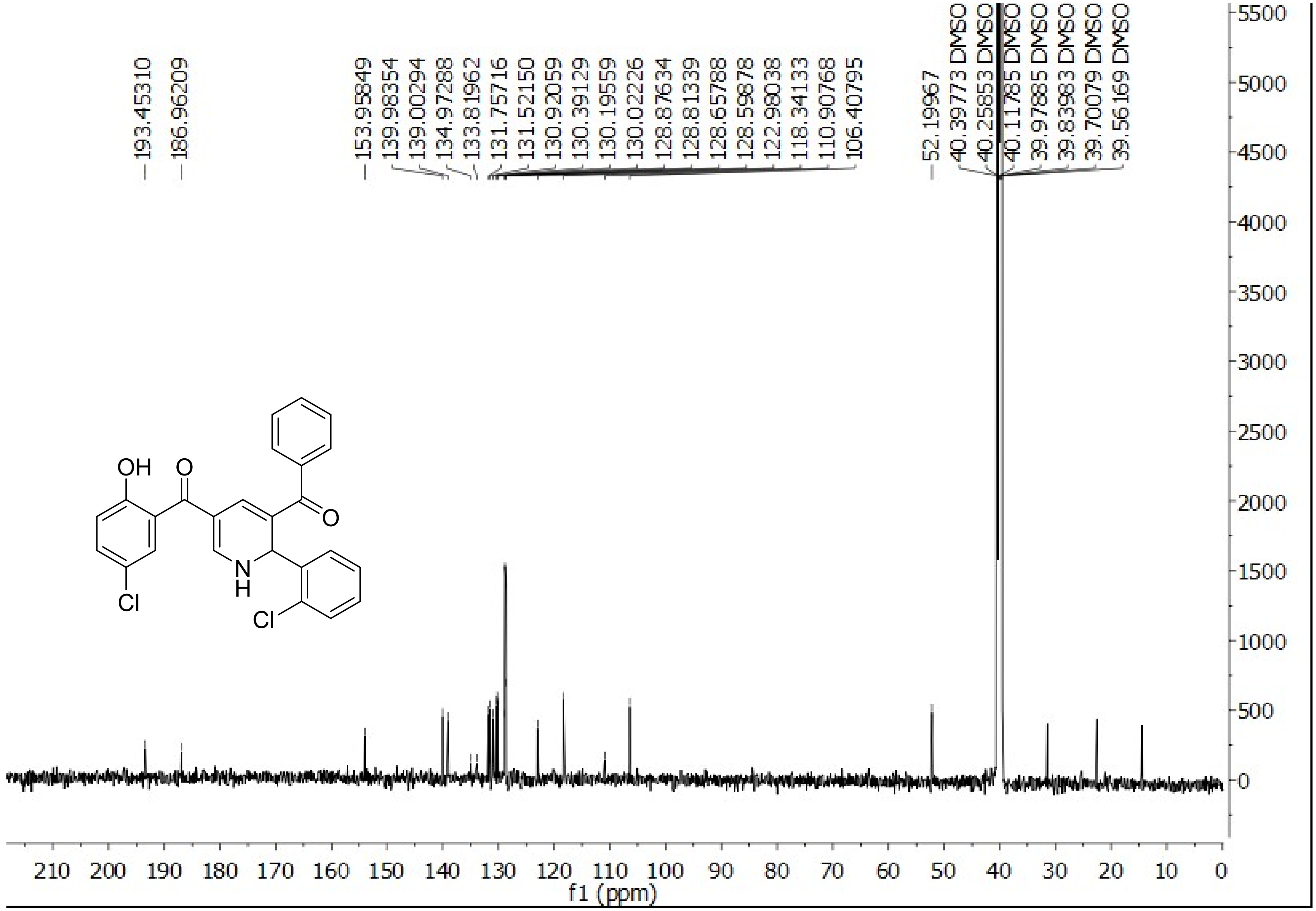

$^{13}C\{^1H\}$ NMR spectrum of **3r** (150 MHz, DMSO-d_6)

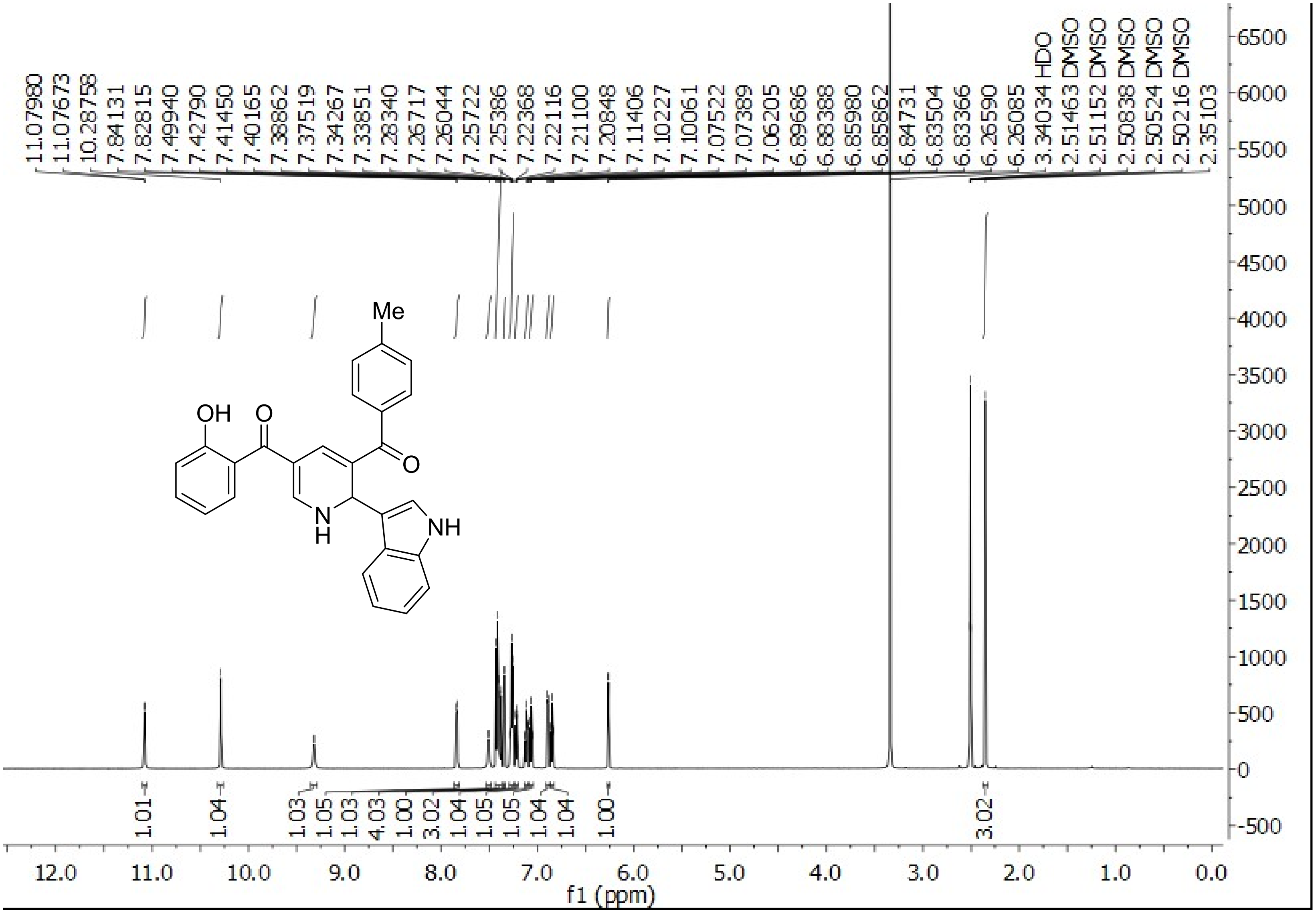

^1H NMR spectrum of **3s** (600 MHz, DMSO-d_6)

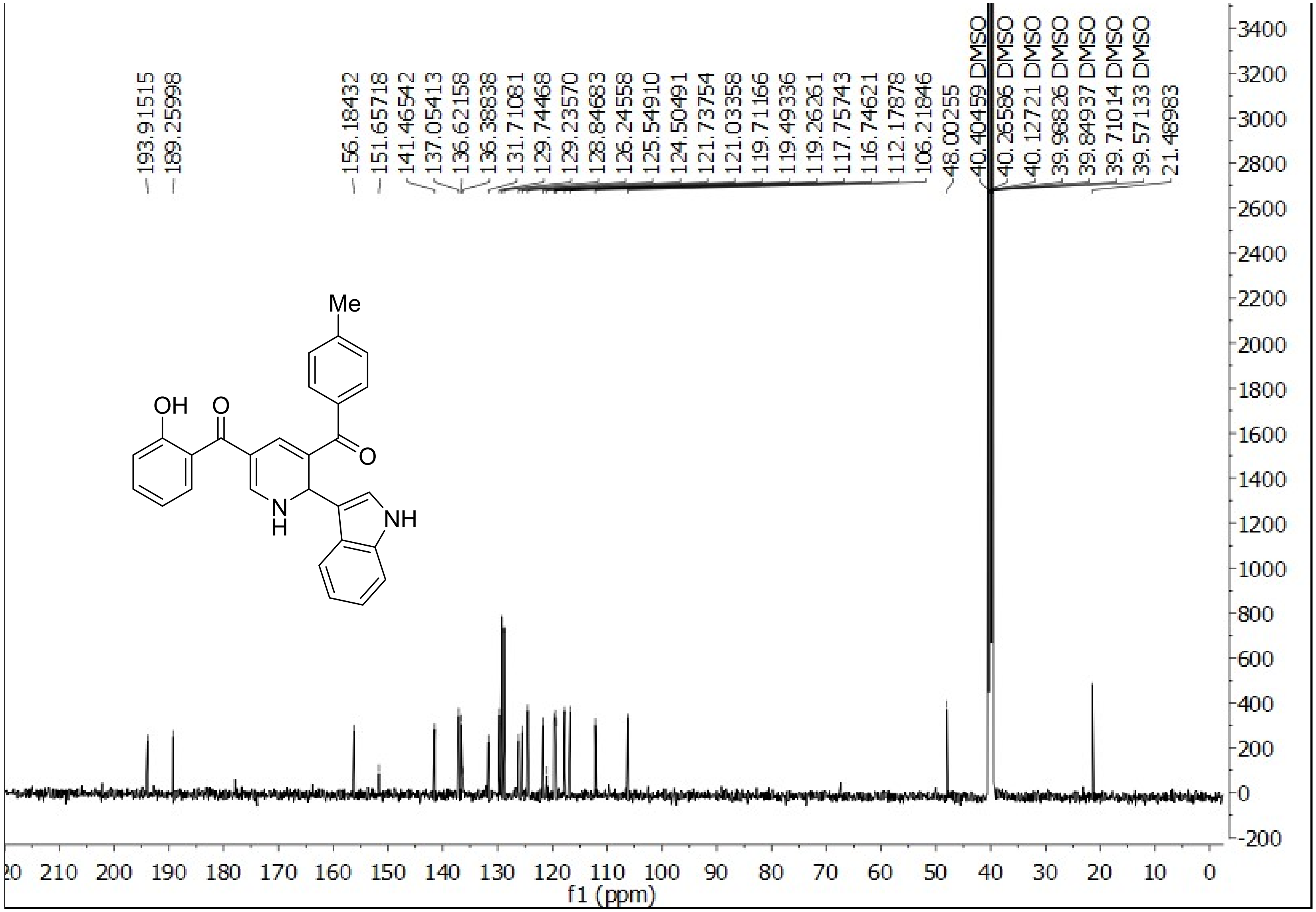

$^{13}C\{^1H\}$ NMR spectrum of **3s** (150 MHz, DMSO-d_6)

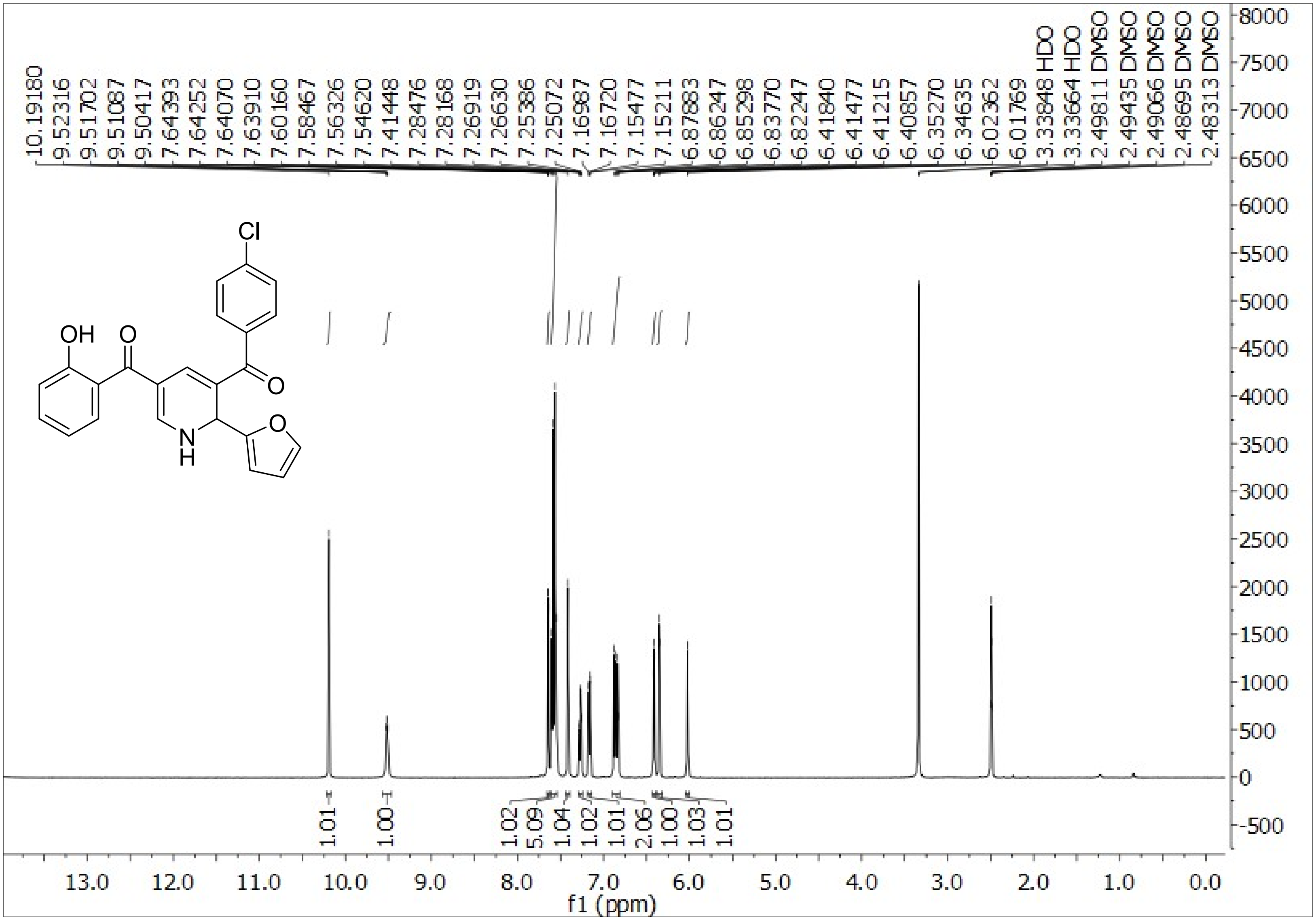

^{1}H NMR spectrum of **3t** (500 MHz, DMSO-d_6)

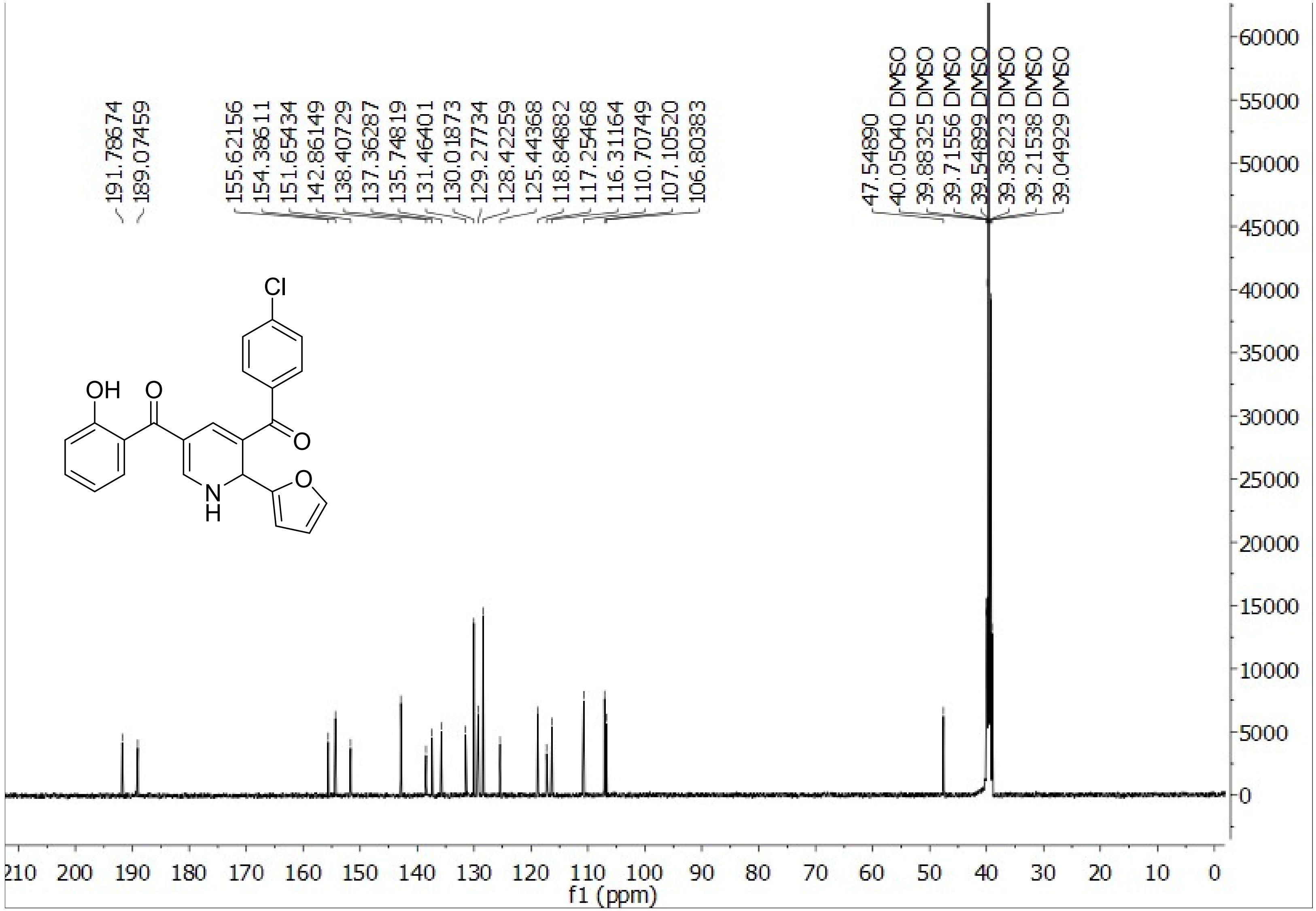

$^{13}C\{^1H\}$ NMR spectrum of **3t** (125 MHz, DMSO-d_6)

The formation method of 3l single crystals

60 mg of compound **3l** was dissolved in 25 mL of dichloromethane in a 50 mL laboratory glass beaker, and then 25 mL of *n*-hexane was added. The beaker was covered with an aluminum foil and several holes were made in the aluminum foil. Crystals of compound **3l** were formed at the bottom of the beaker after 5 days.

Crystal structure description of compound 3l

The X-ray diffraction measurement was made on a STOE IPDS-II diffractometer with graphite monochromated Mo-Kα radiation. For both enantiomers of **3l** (**3l-R** and **3l-S**) plate yellow crystal was chosen using a polarizing microscope and was mounted on a glass fiber which was used for data collection. Cell constants and orientation matrices for data collection were obtained by least-squares refinement of diffraction data from 3845 and 3842 unique reflections for **3l-R** and **3l-S**, respectively. Data were collected to a maximum 2θ value of 50° in a series of ω scans in 1° oscillations and integrated using the Stoe X-AREA[2] software package. The data were corrected for Lorentz and Polarizing effects. The structure was solved by direct methods[3] and subsequent difference Fourier maps and then refined on F^2 by a full-matrix least-squares procedure using anisotropic displacement parameters[4]. All hydrogen atoms attached to carbon were added in idealized positions. Hydrogen atoms of N–H and O–H were found in difference Fourier maps. The atomic factors were taken from the International Tables for X-ray Crystallography[5]. All refinements were performed using the X-STEP32 crystallographic software package[6]. CCDC No. 2364260 and 2364261 contains crystallographic data for this paper. These data can be obtained free of charge from the Cambridge Crystallographic Data Center via www.ccdc.cam.ac.uk/data_request/cif.

*Crystal data for **3l-R**.* $C_{25}H_{17}NO_3Cl_2$, M = 450.30, yellow plate, crystal dimensions: 0.40×0.25×0.20 mm^3; orthorhombic, space group $P2_12_12_1$; a = 9.917(2), b = 13.467(3), c = 16.340(3) Å; V = 2182.2(8) Å^3; T =

298(2) K; Z = 4; D_{calc} = 1.371 g cm^{-3}; μ = 0.325 mm^{-1} (for Mo Kα, λ = 0.71073 Å); F(000) = 928; reflections collected = 6984; reflections independent = 3845 [R_{int} = 0.1411]; ϑ range 1.960 to 24.996; full-matrix least-squares on F^2; parameters = 239; restraints = 7; R_1 = 0.0949; wR_2 = 0.1749 [I>2sigma(I)]; GooF = S = 0.931; largest difference in peak and hole, $\Delta\rho_{max}$ and $\Delta\rho_{min}$ = 0.311 and -0.288 e.Å^3.

*Crystal data for **3l-S**.* $C_{25}H_{17}NO_3Cl_2$, M = 450.30, yellow plate, crystal dimensions: 0.40×0.20×0.20 mm^3; orthorhombic, space group $P2_12_12_1$; a = 9.894(2), b = 13.487(3), c = 16.351(3) Å; V = 2181.9(8) Å^3; T = 298(2) K; Z = 4; D_{calc} = 1.371 g cm^{-3}; μ = 0.325 mm^{-1} (for Mo Kα, λ = 0.71073 Å); F(000) = 928; reflections collected = 6805; reflections independent = 3842 [R_{int} = 0.1116]; ϑ range 1.957 to 24.990; full-matrix least-squares on F^2; parameters = 251; restraints = 2; R_1 = 0.0862; wR_2 = 0.1567 [I>2sigma(I)]; GooF = S = 0.942; largest difference in peak and hole, $\Delta\rho_{max}$ and $\Delta\rho_{min}$ = 0.380 and -0.319 e.Å^3.

References

1. M. Giardinetti, N. I. Jessen, M. L. Christensen, and K. A. Jorgensen, *Chem. Commun.*, 2019, **55**, 202.
2. Stoe & Cie, X–AREA: Program for the Acquisition and Analysis of Data, Version 1.30; Stoe & Cie GmbH: Darmatadt, Germany, 2005.
3. Sheldrick, G. M. SHELX97. Program for Crystal Structure Solution. University of Göttingen, Germany, 1997.
4. Sheldrick, G. M. SHELX97. Program for Crystal Structure Refinement. University of Göttingen, Germany, 1997.
5. International Tables for X-ray Crystallography, Vol C, Kluwer Academic Publisher, Doordrecht, The Netherlands,1995.
6. Stoe & Cie, X-STEP32: Crystallographic Package, Version 1.07b; Stoe & Cie GmbH: Darmstadt, Germany, 2000.
